# Nudge-based misinformation interventions are effective in information environments with low misinformation prevalence

**DOI:** 10.1038/s41598-024-62286-7

**Published:** 2024-05-20

**Authors:** Lucy H. Butler, Toby Prike, Ullrich K. H. Ecker

**Affiliations:** 1 School of Psychological Science (M304), University of Western Australia, Perth, 6009 Australia; 2 Network Science Institute, Northeastern University, Boston, USA; 3 School of Psychology, University of Adelaide, Adelaide, Australia; 4 Public Policy Institute, University of Western Australia, Perth, Australia

**Keywords:** Psychology, Human behaviour

## Abstract

Nudge-based misinformation interventions are presented as cheap and effective ways to reduce the spread of misinformation online. However, despite online information environments typically containing relatively low volumes of misinformation, most studies testing the effectiveness of nudge interventions present equal proportions of true and false information. As the effectiveness of nudges can be highly context-dependent, it is imperative to validate the effectiveness of nudge-based interventions in environments with more realistic proportions of misinformation. The current study (*N* = 1387) assessed the effectiveness of a combined accuracy and social-norm nudge in simulated social-media environments with varying proportions of misinformation (50%, 20%, and 12.5%) relative to true and non-news-based (i.e., “social”) information. The nudge intervention was effective at improving sharing discernment in conditions with lower proportions of misinformation, providing ecologically valid support for the use of nudge-based interventions to counter misinformation propagation on social media.

## Introduction

Misinformation—defined here as false or misleading information presented as accurate, regardless of intent—has the capacity to negatively impact people’s beliefs and behaviors^1^. Behaviors driven or justified in part by misinformation, such as vaccination refusal, can not only detrimentally impact an individual (e.g., increasing the risk of contracting potentially life-threatening illnesses), but also others (e.g., increasing the potential spread of viruses to vulnerable populations) and society more broadly (e.g., placing increased pressure on the health-care system^2–4^). Due to the threats posed by misinformation, developing and implementing interventions that reduce people’s susceptibility to misinformation has become a key focus of both research and policy^1,5,6^.

To date, several psychologically-based misinformation interventions have received sound empirical support (for a synthesis of currently recommended interventions, see Kozyreva et al.^7^). For example, providing corrective information that directly counters a piece of misinformation (i.e., “debunking”) can significantly and meaningfully reduce belief in the targeted false information^8^. However, although targeted interventions can be highly effective, the advent of social media has made detecting and directly counteracting misinformation increasingly difficult, and in many cases impossible^9^. As such, in recent years there has been an increased focus on developing and implementing generalized misinformation interventions that are easily scalable to social-media environments^7,10,11^. Many of these interventions are based on nudge theory, which posits that small changes in choice architecture in the information environment can meaningfully impact decision-making processes^12^. In the realm of misinformation, nudge-based interventions typically attempt to reduce misinformation sharing that may occur due to inattentiveness to information veracity^13^ by priming people to consider (1) the accuracy of encountered information (i.e., accuracy nudges^14,15^) or (2) the attitudes or behaviors of others as they pertain to misinformation sharing (i.e., social-norm nudges^16,17^). Nudge-based misinformation interventions are proposed to be effective because they draw people’s attention to the importance of veracity, subsequently increasing the weight placed on veracity as a criterion during decision-making processes (in line with the limited-attention utility model^13^).

In experimental settings, nudge-based misinformation interventions have generally shown to have a beneficial, though small, impact on engagement behavior through either directly reducing intent to share false information or improving “sharing discernment” (i.e., increasing the proportion of true relative to false information participants report they would share^10,15–18^). However, despite these positive findings, studies assessing the effectiveness of nudge-based misinformation interventions do not always appropriately consider the structure of the social-media information environment. Specifically, (1) the proportion of false information is often artificially high (e.g., 50% of the claims presented), (2) participants are often exposed to only verifiable (i.e., objectively true or false) information, typically in the form of news headlines, and (3) participants are often required to actively appraise whether or not they would engage with each item (e.g., headline). In contrast, the quantity of misinformation people are exposed to on real-world social-media platforms is typically small compared to the amount of true or non-verifiable (e.g., personal or opinion-based) information^19–21^, and the volume of information people are exposed to on social media exceeds what they are able or inclined to critically appraise^13,22–24^.

Research outside of the field of misinformation has shown that nudges can be highly context-specific and susceptible to decay^12,25^. As such, it is unclear whether the effect of nudge-based interventions observed in typical experimental settings would be observed in more realistic information environments. In fact, exploratory analyses by Roozenbeek et al.^18^ suggest accuracy nudges may only be effective on the few posts immediately succeeding the nudge. If this is the case, nudge-based misinformation interventions may be less effective than believed, particularly when misinformation makes up a small proportion of content in the information environment. As the implementation of interventions, especially those which shift responsibility to the individual consumer, potentially have detrimental practical consequences if ineffective^26^, the question of whether the effects of nudge-based interventions translate to more realistic information environments requires direct investigation.

Recent studies have shown that the information composition of the information environment, specifically the relative proportion of true to false information, can significantly influence participants’ susceptibility to misinformation^27,28^. Notably, Orchinik et al.^27^ found that participants were more likely to erroneously classify false (true) information as true (false) when exposed to predominantly true (false) information in the experimental information environment, suggesting that participants responses were biased in the direction of the “veracity base rate” (see also^28^). The effectiveness of media-literacy interventions has also been shown to vary based on the information composition of the environment; one study found that interventions designed to enhance either scepticism or trust were most effective with equal proportions of true and false information in the environment, whereas a combined approach aimed to enhance both scepticism and trust was most effective in a condition with 75% true information (i.e., a proportion similar to real-world environments)^29^. These findings suggest that the information composition of the environment may impact both peoples’ susceptibility to misinformation and the effectiveness of misinformation interventions. However, despite the theoretical basis of nudge-based interventions being rooted in the composition of the social-media information environment, research is yet to assess the effectiveness of nudge-based misinformation interventions across environments with different content compositions.

We are aware of one recent study that assessed the efficacy of an accuracy-nudge intervention in a setting which included non-news-based posts in addition to true and false headlines^30^. Participants were presented with 72 posts, of which 48 were social posts (50% political, 50% apolitical), 12 were true headlines, and 12 were false headlines. The researchers found accuracy prompts led to a small improvement in sharing discernment, however, no improvement in liking discernment (in fact, liking of false posts was numerically greater than control in all accuracy-nudge conditions, in some cases statistically significantly so). Additionally, the accuracy nudge neither significantly reduced engagement with (or sharing of) false information, nor significantly increased engagement with (or sharing of) true information compared to the control condition. This suggests that nudging may have only limited positive impact on sharing discernment in environments with lower volumes of misinformation. However, in this study the proportion of each information type (i.e., true, false, and social) was kept constant across all conditions. As such, it is unclear whether the effectiveness of nudge interventions varies depending on the proportion of misinformation relative to true and non-news information.

Accordingly, the overarching aim of the current study was to assess the effectiveness of a scalable nudge-based intervention (specifically, a combined accuracy-prompt and social-norm intervention) in environments with varied proportions of misinformation. Across conditions, false headlines made up either 50% (40 false headlines, 40 true headlines), 20% (10 false headlines, 40 true headlines), or 12.5% (10 false headlines, 40 true headlines, 30 social posts) of total posts. To further increase external validity, posts were also presented in a mock feed using a realistic social-media simulator^31^, and participants were informed that they could scroll past posts without engaging (i.e., as with real social-media platforms, participants were not required to actively attend to or engage with posts).

We specified the following pre-registered hypotheses: It was hypothesized that (1) there would be a beneficial effect of the nudge intervention, such that the nudge would significantly improve engagement (i.e., both sharing and liking) discernment. Further, and central to the current research question, it was hypothesized that (2) the effectiveness of the nudge intervention would depend on the misinformation proportion. As this is the first time this question has been empirically assessed, we did not explicitly pre-register a directional hypothesis. However, due to the context-dependent nature of nudges, and prior research suggesting the effectiveness of nudge-based misinformation interventions may decay relatively quickly^18^, if the effectiveness of the nudge intervention does differ across conditions with varying proportions of misinformation it was predicted that the nudge intervention would be significantly more effective when the proportion of false headlines was high (i.e., 50%) than in the conditions with a lower proportion of false headlines (i.e., 20% and 12.5%).

## Method

This study was pre-registered at https://osf.io/ch7n9. This study had a 2 × 3 between-subjects design with factors nudge intervention (present, absent) and misinformation proportion (50%, 20%, 12.5%). Each participant was therefore randomly assigned to one of six possible conditions. Given the study included both true and false headlines, the design was technically a 2 (presence of nudge) × 3 (misinformation proportion) × 2 (headline veracity) between-within-subjects design. The primary dependent variables were sharing and liking discernment (i.e., difference in sharing and liking of true and false headlines). Discernment was chosen as the primary outcome variable to be consistent with prior research^14–16,18,32^. However, because the effectiveness of nudge interventions is often framed in terms of reducing engagement with (particularly sharing of) false information, we also looked at the impact of the nudge on false and true headlines in isolation. Note that we slightly deviate from the pre-registered analysis plan where we specified a composite variable of sharing and liking behavior (i.e., engagement behavior) as the primary outcome variable. The justification for this deviation is provided in the Results section, and results for combined engagement behavior are provided in Supplement B.

### Participants

An a-priori power analysis (using G*Power^33^) suggested a minimum sample size of 1269 (approximately 212 participants per condition) to detect a small effect of *f* = 0.10 at α = 0.05 and 1−β = 0.90. To ensure adequate sample size following anticipated exclusions, we thus aimed to sample 250 participants per cell (1500 participants total). A total of 1501 U.S.-based participants with at least one social media account (e.g., Facebook, Instagram, TikTok) were recruited via Prolific. Due to a misinterpretation of instructions, 56 participants terminated the experiment before completing the belief-rating questions at the end of the experiment. Given these participants completed the primary task, and consented to their data being used, their data was retained for the primary analyses. Participants were excluded in accordance with the following pre-registered criteria: (1) Self-reported English proficiency “fair” or “poor” (*n* = 0); (2) self-reported lack of effort (*n* = 2); (3) completion time for the simulated feed portion of the study < 3 min for the 12.5% and 50% misinformation conditions or < 2 min for the 20% misinformation condition (*n* = 21); (4) post dwell time in the simulated feed equal to 0 ms for more than 10% of posts (*n* = 68); (5) median dwell time across all posts < 2000 ms (*n* = 65); (6) an identical response to > 80% of the belief ratings (*n* = 22). An additional six participants were excluded for completing the simulated feed portion of the study multiple times. Accounting for participants who met multiple exclusion criteria, 114 participants were excluded, resulting in a final sample size of *N* = 1387 (1335 of which completed the belief ratings; *M*_age_ = 41.39, *SD*_age_ = 14.14; age range 18–85; 736 females, 621 males, 26 non-binary individuals, 2 transgender men, 1 transgender individual, 1 participant who reported no gender). The number of participants in each condition is displayed in Table 1. We note there was a significant main effect of misinformation proportion on attrition post-exclusion criteria being applied, χ^2^(2) = 8.89, *p* = 0.012, though follow-up contrasts revealed no significant differences in level of attrition across the pairs of misinformation proportion conditions (*p* ≥ 0.082). Given the lack of a significant effect of attrition in the follow-up contrasts, results presented in-text are run on the dataset post-exclusion criteria being applied as per the pre-registration. However, main analyses were also conducted on the complete dataset (i.e., prior to the implementation of exclusion criteria) and the pattern of results for both liking and sharing behavior are equivalent (see Supplement D, Tables D7 and D13).

**Table 1:** Number of participants split by nudge and misinformation proportion conditions.

Nudge condition	Misinformation proportion	*n*
No nudge	12.5%	221
	20%	250
	50%	238
Nudge	12.5%	223
	20%	223
	50%	232

### Materials

#### Nudge intervention

The nudge comprised a combined accuracy and social-norm prompt. The prompt was conceptually modelled off prior research^15–17^; however, the exact content of the accuracy prompt slightly differed from that used in most accuracy-prompt research in that the accuracy prompt was made explicit. This alteration was made to maximize statistical power for observing a differential effect of the misinformation-proportion conditions, given effect sizes observed in accuracy-prompt research are typically small to very small^15,34^. Specifically, participants in the nudge conditions received the following information: *“It is important to consider the accuracy of posts when engaging with content online. In fact, it is widely accepted that spreading misinformation is wrong and can have a variety of negative outcomes for both individuals and societies. Indeed, a recent study found that more than 80% of U.S. adults think it’s very important to only share accurate content online. This was true for both Democrats and Republicans. As such, please consider the accuracy of the headline on the following page.”* Participants were then presented with a single false headline (“Newborn becomes first to be named an emoji”; See online Appendix A, Figure A1), which was identical across participants, and were asked to rate the headline’s accuracy on an 11-point scale from 0 (“Certainly false”) to 10 (“Certainly true”).

**Figure 1:**
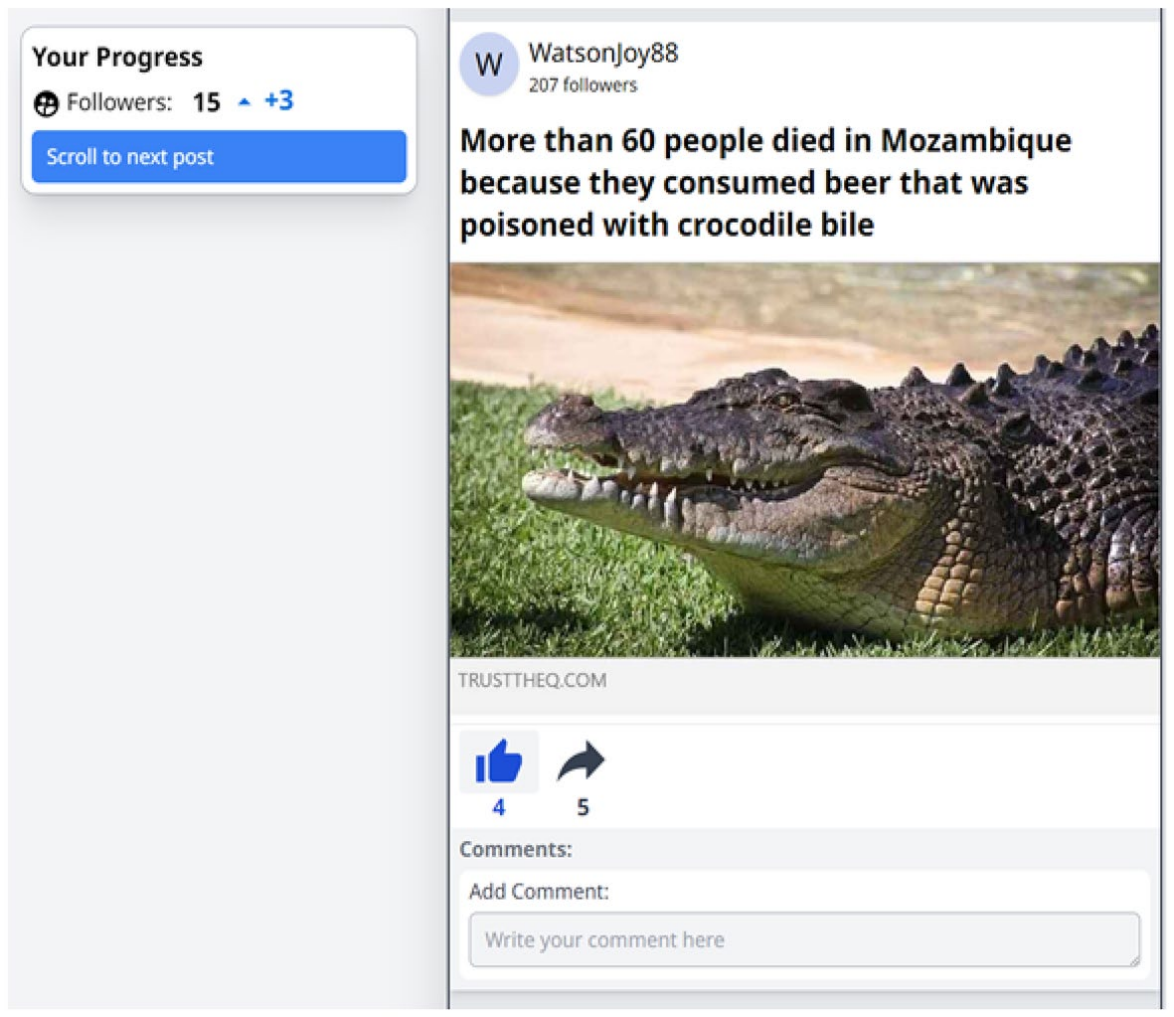
An Example False Post. Note, this example is from the 20% misinformation condition. In this instance, the participant engaged with the post by “liking” it. The participant’s current follower count is 15 (including a change of + 3 based on an engagement with a previous post). Image used in post taken from: Saltwater crocodile [Photograph], by L. Jones, 2012, Flickr, https://www.flickr.com/photos/ljcjones/7636290144/CCBY-SA2.0DEED.

#### Posts

Dependent on condition, participants were presented with either (1) 40 true news headlines and 40 false news headlines (50% misinformation condition), (2) 40 true news headlines and 10 false news headlines (20% misinformation condition), or (3) 40 true news headlines, 10 false news headlines, and 30 social (i.e., non-falsifiable) posts (12.5% misinformation condition). Note that, given the reduced number of posts presented in the 20% misinformation condition, social posts were included in the 12.5% misinformation condition to match the total number of posts in the 50% misinformation condition without introducing additional true information.

We initially selected 40 true and 40 false headlines from a pool of 60 false and 60 true headlines. False headlines were sampled from a range of sources such as^35^, Snopes.com, and Politifact.com and true headlines were sampled from a range of reputable news sources such as NPR, BBC, and CNN. All true headlines were cross verified with at least one other news source to validate its veracity. In two pilot ratings, all 120 headlines from the initial pool were rated on believability and political leaning by a sample of *N* = 50 Prolific workers (24 male, 25 female, one non-binary; *M*_age_ = 38.96, *SD*_age_ = 12.69), and on shareability and “currentness” by a separate sample of *N* = 52 Prolific workers (26 male, 25 female, one non-binary; *M*_age_ = 41.10, *SD*_age_ = 12.72; see Supplement A for full details). Selected true and false headlines were approximately matched on political leaning, shareability, and currentness; believability was substantially lower for false than true headlines. Ten false headlines were selected to be the target headlines used in all conditions (and thus the main analyses), maintaining comparable believability, political leaning, shareability, and currentness to the full set of false headlines used in the 50% misinformation condition.

Each headline was paired with either the image used in the original headline, or an image relevant to the headline when the original image was not available (e.g., due to copyright reasons or content removal). Headlines were also paired with either a mainstream (for true headlines; e.g., BBC) or non-reputable (for false headlines; e.g., REALRAWNEWS.COM) real-world news source. For the target false headlines, a unique non-reputable source was used for each headline (i.e., 10 sources total). In the 50% misinformation condition, three additional false headlines were paired with each non-reputable source, resulting in four false headlines being paired with each non-reputable source. For the true headlines, four headlines were paired with each mainstream source (i.e., 10 sources total). Pairing of headlines to news sources was fixed across participants. See Fig. 1 for an example post.

Social posts used in the 12.5% misinformation condition were generated by the authors and paired with a relevant royalty-free image. Posts were designed to be non-controversial and mimic typical user-generated content people may encounter on social media (e.g., buy and sell, holiday posts, similar to^30^); however, social posts were not pilot tested.

For each participant, each post was randomly paired with a unique post source (i.e., the source that ostensibly shared the post into the feed, additional to the source of the news headline) with a generic username (e.g., k_hannam), and each post source had a randomly determined number of followers (sampled from a positively skewed distribution with *M* = 0, *SD* = 500, *skewness* = 10, truncated at 0). Some additional parameters were also determined probabilistically: Posts were associated with a small number of prior likes and shares (ostensibly from other users), and participants’ follower counts changed dynamically depending on their engagement with posts (e.g., liking, sharing). Parameters of the normal distributions from which values were randomly sampled are given in Table 2. To avoid potential confounds that may arise from behavioral feedback, parameters were kept constant across post type (false, true, and social), and misinformation-proportion and nudge conditions; however, the specific values were randomized across participants.

**Table 2:** Normal-distribution parameters used to determine the quantity of prior engagements associated with each post and the impact of post engagements on participant follower counts.

	Quantity of prior engagements		Changes to followers	
	*M*	*SD*	*M*	*SD*
Likes	5	10	+ 2	2
Shares	1	3	+ 4	2

Distributions were truncated at 0 for prior-engagement quantities but not changes to followers (which were therefore sometimes negative).

### Procedure

Participants were initially provided with an ethics-approved information sheet and provided informed consent and basic demographic information. Participants were then shown instructions on how to interact with the simulated social-media feed, and were informed that they did not have to interact with posts if they did not wish to. They were then randomly assigned to one of the six conditions. In the nudge conditions, participants were initially presented with the combined accuracy and social-norm nudge. Because engagement is, to a degree, incentivised on social media^31^ before beginning the task all participants also received the prompt *“Remember: Engage as you would on social media and try to maximize your follower count!”*; however, participant’s final follower count had no real-world (e.g., monetary) consequence*.*Participants were then presented with all posts, in a randomized order, in a feed format (i.e., all posts were displayed on a single page, requiring participants to scroll down the page to view subsequent posts). Participants had the option to like and/or share, as well as comment, on each post. Participants could also not engage with a post by scrolling past it. Engagement was recorded, and follower counts were updated (where pertinent) once participants scrolled past the post (i.e., it was no longer visible on screen). Once recorded, participants could not change how they interacted with a post; however, participants could “scroll back” and engage with posts they had previously not interacted with.

After completing the primary component of the study (i.e., the social-media simulation), participants were presented with a subset of the headlines again (without the news source or image), and were asked to rate their belief in each headline on an 11-point scale from 0 (“Certainly false”) to 10 (“Certainly true”). To maintain the true:false proportions used in the main task, participants in the 12.5% and 20% misinformation conditions were presented with all 10 false and 40 true headlines presented in the social-media simulation. Participants in the 50% misinformation condition were presented with a subset of 25 false (always including the 10 target false headlines) and 25 true headlines in one of 16 counterbalanced combinations. All headlines were displayed for a minimum of 3 s. Participants were then asked a single question regarding their political orientation (specifically, “*Where would you position yourself politically from strongly liberal to strongly conservative?”*); responses were recorded on a 7-point Likert scale from 0 (“Strongly liberal”) to 6 (“Strongly conservative”). Upon completion, participants were asked whether their data should be discarded due to lack of effort and were provided a debriefing, which explicitly stated the purpose of the study as well as the veracity of all the presented true and false headlines. Median completion time was approximately 20 min and participants were compensated £2.20 (approx. US$2.75) for their time.

### Ethics approval and consent to participate

All procedures were approved by the University of Western Australia’s Human Research Ethics Office (Ethics ID: 2019/RA/4/20/6423) and complied with all relevant guidelines and the Declaration of Helsinki. All participants provided informed consent prior to participating.

## Results

### Analytic approach

All data analyses and visualizations were performed in R version 4.3.2^36^. Main analyses were conducted using the *glmer* function of the *lme4* package^37^. Because we were interested in overall main effects and interactions, rather than only main effects and interactions relative to the reference group, we conducted analyses of deviance^38^ on the fitted models using the *Anova* function of the *car* package^39^. Post-hoc comparisons were conducted using *emmeans*^40^, and all data visualizations were created using *ggplot2*^41^. Data and the R script, Rmarkdown file, and supplementary information for all analyses is available at https://osf.io/nztuk/.

We initially pre-registered “engagement” as the primary outcome variable, whereby engagement would be coded as an ordinal factor with four levels (0 = no engagement, 1 = like, 2 = share, 3 = like and share). However, the pre-registered cumulative-link mixed effect models required to assess engagement behavior failed to converge with both participant and item random effects included in the model, resulting in a reliance on suboptimal models for analyses^42^. Accordingly, based on reviewer feedback, we deviated from the pre-registered analysis plan and treated sharing and liking as two discrete outcome measures (each measured on a binary scale), and analyzed the effects of the nudge intervention and misinformation proportion on both sharing and liking behavior using logistic mixed effects models. Analyses of “engagement behavior”—treated both as an ordinal variable and a binary variable (0 = did not engage; 1 = either liked or shared or both)—are presented in full in Supplements B and D; results of these analyses are generally consistent with the analyses of sharing behavior presented below, unless noted otherwise.

Prior to statistical analysis, the factors nudge (present, absent) and headline veracity (true, false) were centred, and misinformation proportion (12.5%, 20%, 50%) was factor-coded. The maximal random-effect structure justified by the design was included^42^; specifications of random-effect structures for each model are available in Supplement D. Main analyses presented here focus only on sharing and liking of the true and false headlines; analyses of participant engagement with the social posts (featured only in the 12.5% misinformation condition) are presented in Supplement C. Furthermore, belief and political orientation were included as secondary variables; results are not included in the main text, but see Supplement C for an overview of all supplementary analyses.

### Impact of nudge intervention and misinformation proportion on sharing discernment

**Figure 2:**
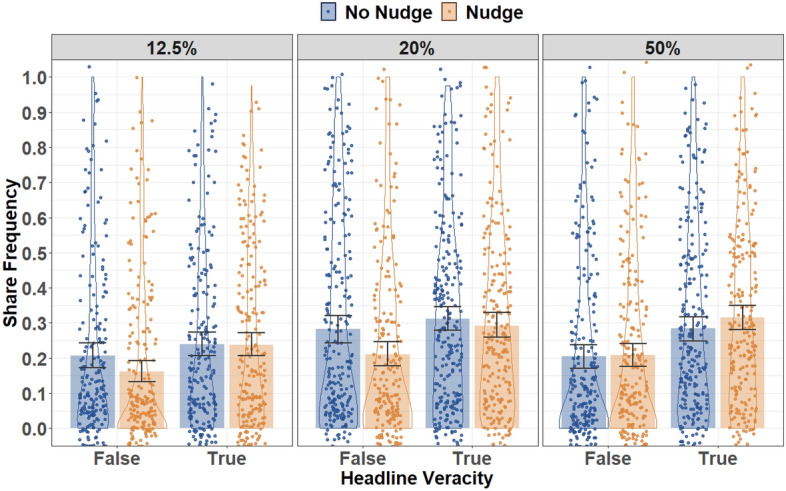
Sharing Frequency for False and True Headlines Across Nudge and Misinformation Proportion Conditions. Note that 12.5%, 20%, and 50% Refer to the Corresponding Misinformation-Proportion Conditions. Bars Show Condition Means; Error Bars Represent 95% Confidence Intervals; Jittered Dots Represent Individual Participant Means; Violins Provide Distributional Information.

We first assessed the impact of the nudge intervention on sharing discernment across misinformation-proportion conditions. Sharing frequency across conditions (nudge condition, misinformation proportion) and headline veracity is displayed in Fig. 2; results of the ANODEs for sharing behavior are displayed in Table 3. Further information on participants’ engagement behavior at the headline level is available in Supplement A (Tables A5 and A6).

**Table 3:** ANODE (Type 3) results for sharing behavior.

Fixed effects	χ^2^	*df*	*p*
Misinformation proportion	19.19	2	< .001
Nudge	1.90	1	.168
Headline veracity	24.93	1	< .001
Misinformation proportion × nudge	3.50	2	.174
Misinformation proportion × headline veracity	8.96	2	.011
Nudge × headline veracity	7.15	1	.007
Misinformation proportion × nudge × headline veracity	1.51	2	.470

Consistent with the first hypothesis, the nudge improved sharing discernment (i.e., a nudge × headline veracity interaction); that is, the nudge condition was associated with increased sharing of true relative to false headlines. However, contrary to the second hypothesis, there was no significant three-way misinformation proportion × nudge × headline-veracity interaction, suggesting that the impact of the nudge intervention on sharing discernment did not significantly differ across misinformation-proportion conditions. There was, however, a significant misinformation-proportion × headline-veracity interaction, suggesting participants’ engagement with true and false posts did vary across misinformation-proportion conditions.

To deconstruct the nudge × headline-veracity interaction, and thus directly assess whether the nudge intervention significantly reduced sharing of false posts or significantly increased sharing of true posts (or neither), we ran pre-registered follow-up analyses isolated to false and true headlines (collapsed across misinformation-proportion conditions). Participants in the nudge condition shared significantly fewer false posts than those in the no-nudge condition; *OR* = 1.44, *SE* = 0.22, *z* = 2.42, *p* = 0.016. By contrast, there was no significant difference in sharing of true headlines across the nudge and no-nudge conditions, *OR* = 0.95, *SE* = 0.12, *z* = 0.43, *p* = 0.667.

Although there was no significant three-way interaction, for completeness we also assessed the impact of the nudge on sharing discernment isolated to each misinformation-proportion condition. The nudge intervention improved sharing discernment in both the 12.5% and 20% misinformation conditions; χ^2^(1) = 7.04, *p* = 0.008 and χ^2^(1) = 10.92, *p* = 0.001 respectively. By contrast, there was no significant effect of the nudge intervention on sharing discernment in the 50% misinformation condition; χ^2^(1) = 3.49, *p* = 0.062, however, again be aware that the effect of the nudge in the 50% misinformation condition did not significantly differ from the other two conditions. Pre-registered analyses isolated to false and true headlines revealed that the nudge was associated with a marginally significant reduction in false-headline sharing in both the 12.5% and 20% misinformation conditions; *OR* = 1.76, *SE* = 0.49, *z* = 2.05, *p* = 0.040 and *OR* = 1.70, *SE* = 0.40, *z* = 2.22, *p* = 0.026 respectively. There was no statistically significant effect of the nudge intervention on true headline sharing in either condition (*p*s > 0.666).

Finally, to deconstruct the significant misinformation-proportion × headline-veracity interaction on sharing behavior, we ran post-hoc analyses, Holm-Bonferroni corrected, assessing the impact of misinformation proportion on sharing of false and true headlines, collapsed across nudge conditions. Sharing of false headlines was significantly lower in the 12.5% misinformation condition than the 20% misinformation condition, *OR* = 0.48, *SE* = 0.09, *z* =  − 3.92, *p* < 0.001. Neither the 12.5% nor the 20% conditions differed significantly from the 50% condition in false-headline sharing (*p*s > 0.067). Sharing of true headlines was also significantly lower in the 12.5% condition than either the 20% (*OR* = 0.54, *SE* = 0.08, *z* =  − 4.11, *p* < 0.001) or the 50% (*OR* = 0.56, *SE* = 0.08, *z* =  − 3.83, *p* < 0.001) conditions. There was no significant difference in true headline sharing across the 20% and 50% misinformation conditions (*p* = 0.785).

### Impact of nudge intervention and misinformation proportion on liking discernment

**Figure 3:**
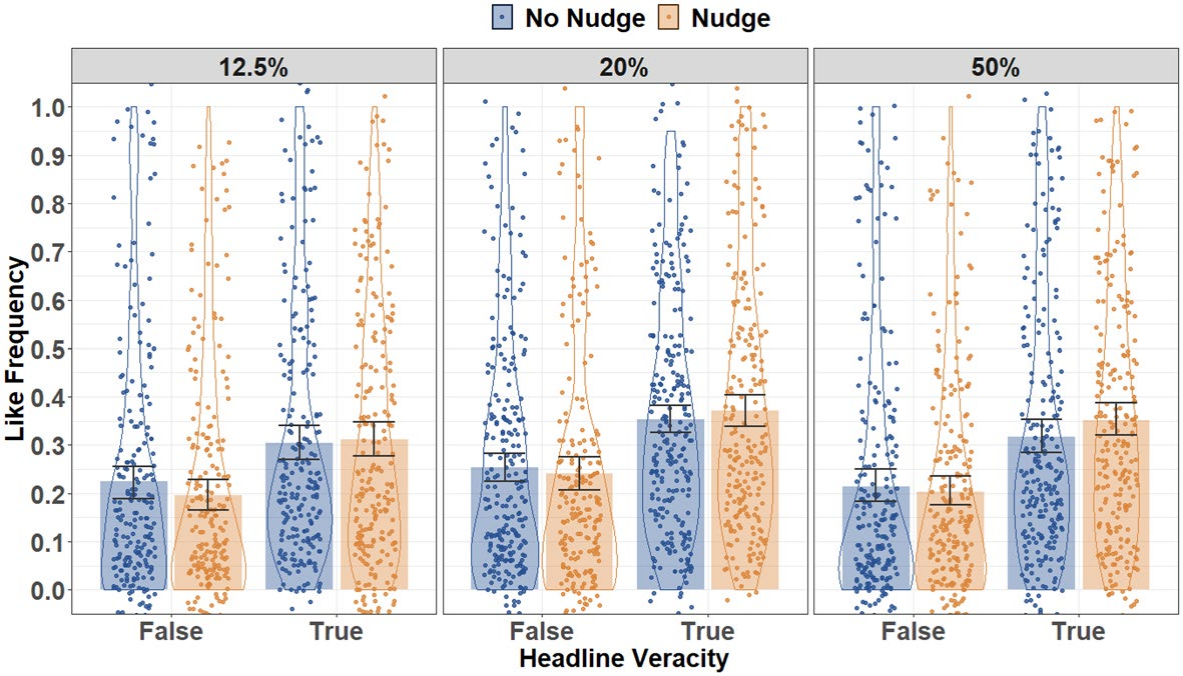
Liking Frequency for False and True Headlines Across Nudge and Misinformation Proportion Conditions. Error Bars denote 95% Confidence Intervals.

We then assessed the impact of the nudge intervention on liking discernment across misinformation-proportion conditions. Liking frequency across conditions (nudge condition, misinformation proportion) and headline veracity is displayed in Fig. 3; results of the ANODEs for liking behavior is displayed in Table 4.

**Table 4:** ANODE (Type 3) results for liking behavior.

Fixed effects	*χ*^*2*^	*df*	*p*
Misinformation proportion	12.60	2	.002
Nudge	0.46	1	.500
Headline veracity	22.15	1	< .001
Misinformation proportion × nudge	1.05	2	.592
Misinformation proportion × headline veracity	3.32	2	.190
Nudge × headline veracity	3.50	1	.061
Misinformation proportion × nudge × headline veracity	0.07	2	.968

Contrary to the first hypothesis and the results of sharing discernment, there was no significant effect of the nudge intervention on liking discernment (i.e., a nudge × headline-veracity interaction). There was additionally no significant three-way misinformation-proportion × nudge × headline-veracity interaction, suggesting the impact of the nudge intervention on liking behavior did not vary across misinformation-proportion conditions. As such, the current results do not support the second hypothesis. However, there were significant main effects of headline veracity, with participants liking true headlines significantly more than false headlines, and misinformation proportion, suggesting participants’ overall level of engagement varied across misinformation-proportion conditions.

To deconstruct the significant effect of misinformation proportion on liking behavior, post-hoc analyses were run on liking of headlines, collapsed across nudge condition and headline veracity. Liking of headlines was significantly higher in the 20% misinformation condition than either the 12.5% (*OR* = 0.65, *SE* = 0.08, *z* =  − 3.43, *p* = 0.002) or the 50% (*OR* = 1.34, *SE* = 0.17, *z* = 2.37, *p* = 0.036) conditions. We note, however, that exploratory follow-up analyses suggested these differences in liking of headlines may have been partially driven by the reduced number of headlines in the 20% misinformation condition (see Supplement C, Tables C10 and C11).

### Does the effectiveness of the nudge intervention decay over time?

**Figure 4:**
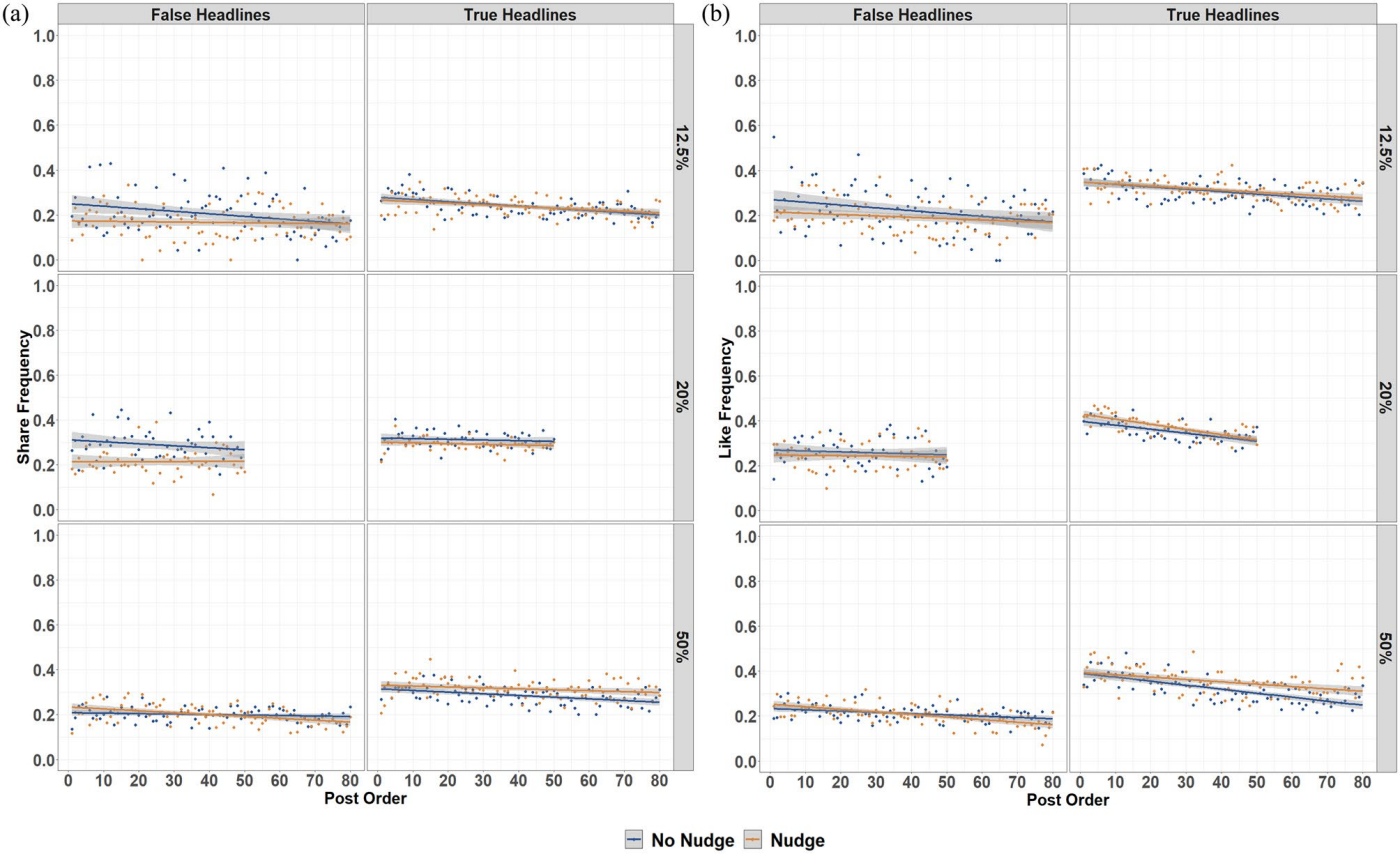
Frequency of Sharing (**a**) and Liking (**b**) of False (Left Panel) and True (Right Panel) Headlines over the Course of the Experiment. *Note* 12.5%, 20%, and 50% refer to the corresponding misinformation-proportion conditions. Shaded areas represent standard error. Jitters represents mean engagement in the no nudge (blue) and nudge (orange) conditions at each post position (as a proxy for timepoint).

Although not pre-registered, we ran exploratory analyses to assess whether the effect of the nudge intervention on sharing discernment decayed over the course of the study. To do so, we included headline-display order in the model as a continuous predictor (as a proxy for time), and analyses were conducted separately for each misinformation-proportion condition. There was no nudge × post-order, or nudge × headline-veracity × post-order interactions on sharing behavior (all *p*s ≥ 0.138; see Supplement C, Tables C21–C24 for full results). As such, there is no strong statistical evidence for (linear) decay over the course of the study. However, we note that (1) we are likely underpowered to be able to test for an effect of order, and (2) the difference in sharing of false headlines between the nudge and no-nudge conditions did appear to numerically decrease over time in the 12.5% and 20% misinformation conditions (see Fig. 4; though the reverse pattern seems to arise for engagement with true information in the 50% misinformation condition).

## Discussion

The current study provides support for the use of nudge-based misinformation interventions across information environments with varied, particularly reduced, proportions of misinformation. Contrary to our predictions, the results suggest the nudge-based intervention was at least equally effective at improving sharing discernment in environments with lower misinformation prevalence compared to a “balanced” environment with 50% misinformation used in most research. Indeed, although there was no significant difference in sharing discernment across misinformation-proportion conditions (i.e., no significant three-way interaction), when analyses were isolated to each of the misinformation-proportion conditions, the nudge significantly improved sharing discernment in the 12.5% and 20% conditions, but not the 50% condition (though the results were not significantly different from the other two conditions, and we note this effect was statistically significant for the combined outcome variable of engagement discernment, both when treated as an ordinal [as pre-registered] and a binomial factor). Thus, findings suggest that the effectiveness of nudge interventions is, at a minimum, not hindered in environments with low proportions of misinformation, and may in fact be slightly enhanced under such conditions. As real-world social-media environments typically contain a relatively low proportion of false information^19^, these findings therefore provide ecologically valid support for the use of large-scale nudge-based misinformation interventions on social media.

Though somewhat speculative, one potential explanation for somewhat smaller (though not significantly different) effect of the nudge in the 50% misinformation condition is that the comparatively high proportion of false posts in this condition may have, to a degree, implicitly primed participant to consider accuracy during their decision-making processes. This would be consistent with the limited-attention utility model proposed by Pennycook and colleagues^13,15^, as well as recent work showing people’s susceptibility to misinformation is lower in environments with a high proportion of misinformation, likely due to people’s intuitive responses being influenced by the veracity base rate of the environment^27^. If high proportions of false information do, to any extent, prime accuracy, using stimuli sets with a 50% true:false ratio could thus potentially lower the observed effectiveness of nudge interventions in experimental settings. However, additional research is needed to both directly test this possibility and tease apart any comparative effect of implicit, context-driven accuracy primes and explicit nudges on sharing behavior.

Notably, follow-up contrasts deconstructing the significant effect of the nudge intervention on sharing discernment revealed that exposure to the nudge led to a significant, though admittedly small, reduction in sharing of false headlines without meaningfully impacting sharing of true headlines. Thus, the positive nudge effect occurred primarily through reducing engagement with misinformation, providing additional support for the notion that sharing of misinformation on social media is likely driven at least in part by inattentiveness to accuracy^14^.

In contrast to sharing discernment, the nudge intervention had no significant effect on liking discernment. Though exploratory analyses revealed that the difference in the nudge effect on sharing and liking was, in itself, nonsignificant (note, however, that the current study was likely underpowered to test for such a difference). These findings suggests that nudge interventions primarily influence sharing behavior, a pattern generally consistent with both past research (e.g.,^30,43^) and the desired intent of the intervention. Specifically, nudge-based interventions are designed to reduce the overall propagation of misinformation, rather than any form of positive engagement. Thus, a minimal impact on liking behavior is likely relatively inconsequential, especially considering the beneficial effect of the intervention on sharing behavior and the numerical pattern of results for liking behavior being in the hypothesised direction. Furthermore, it is possible that in some instances the nudge intervention shifted participants’ engagement with false information from sharing to liking (and from liking to not engaging) rather than from sharing to not engaging, which may have reduced the observed direct effect of the intervention on liking behavior. Regardless, the nudge intervention led to both a net improvement in overall engagement quality (further supported by supplementary analyses combining liking and sharing behavior into a single composite variable, as per the pre-registration; see Supplement B) and directly improved the quality of engagement intended to propagate information. As such, on the whole the current findings provide direct support for nudge-based misinformation interventions as a mechanism to improve the average quality of information spread online.

Beyond the impact of the nudge intervention, misinformation proportion and the number and type of posts seemingly had broader effects on participants’ engagement with both true and false headlines. Specifically, sharing of false posts was significantly higher in the 20% than in the 12.5% misinformation condition, and supplementary analyses suggested this was not simply driven by the reduced number of headlines in the 20% condition (nor participants sharing more at the beginning of the study; see Supplement C, Tables C8 and C9). Sharing of false headlines was not significantly different across the 12.5% and 50% or 20% and 50% misinformation conditions when averaged across nudge conditions (*p*s ≥ 0.068, though we note that tendency to generally engage with [i.e., like or share] false headlines was higher in the 20% than 50% misinformation condition, *p* = 0.042; see Supplement D, Table D15). Sharing of true headlines was also higher in both the 20% and 50% misinformation conditions compared to the 12.5% condition, but was similar across the 20% and 50% conditions. Comparatively, participants liked both true and false posts significantly more in the 20% misinformation condition than either the 12.5% or 50% misinformation conditions; however, this effect appeared to be primarily driven by the reduced number of headlines (50 vs. 80) in the 20% misinformation condition, with people liking more at the start than the end of the study (see Supplement C, Tables C10 and C11). As such, we focus the subsequent discussion on what may have driven the reduced sharing of news content in the 12.5% misinformation condition only.

The 12.5% condition was the only condition to include social posts alongside the news posts, and liking of social posts was notably higher than engagement with either true or false news headlines (see Supplement C, Tables C27–C30). As the reduced level of sharing in the 12.5% condition compared to the 20% condition was relatively equivalent across true and false headlines, and sharing of true headlines was also lower in the 12.5% than the 50% misinformation condition, it appears that people may simply prefer engaging with social content over sharing news-based content. This suggests that although people may be somewhat inattentive to veracity on social-media platforms, they may also be somewhat disinterested in or unwilling to actively share news-based posts on such platforms^44,45^. People’s likelihood of sharing news information may thus generally be lower than suggested by studies that only use news-based stimuli—a factor that should be taken into consideration in subsequent research on online sharing.

Some limitations of the current study should also be addressed in future research. First, while the procedure mirrored prior research, the temporal distance between the provision of the nudge intervention and participants engaging with news headlines was brief, and we therefore cannot make claims about the enduring effectiveness of the intervention. In fact, when assessing the nudge effect across headline display order (as a proxy for time), there was minimal numeric difference in sharing between the nudge and no-nudge conditions by the end of the task in a 12.5% misinformation environment. However, the reduction in nudge effectiveness was not statistically significant, and the effect appeared to last longer than the effect observed in Roozenbeek et al.^18^. Furthermore, a recent field experiment run on social media platforms Facebook and X found no significant decay in the effectiveness of nudge interventions when participants were nudged repeatedly (three unique nudges per day) over a week period^46^. These findings suggest the effectiveness of nudges may not be as fleeting as suggested by Roozenbeek et al., but future research should directly assess how long the effectiveness of nudge-based interventions is maintained.

Second, the effectiveness of the nudge intervention was assessed on news headlines only. While headlines are a common focus of misinformation research, we cannot generalize to other forms of content^26^. Misinformation comes in many forms, and can be more ambiguous and subtle than the false headlines used here^24,26^. As such misinformation and misleading content has the potential to do notable harm (e.g.,^4^), it would be beneficial to assess whether the current results generalize across a broader range of content types^26^.

Third, the behavioral feedback provided to participants in the current study was kept consistent for true and false posts. Though this was done to avoid a potential confounding impact on engagement behavior, future research should attempt to assess if, and how, different incentive structures present in social-media environments impact the effectiveness of nudge interventions.

Finally, despite testing the effectiveness of the nudge intervention in conditions with reduced misinformation, the proportions of misinformation used in this study remained somewhat higher than may be the case in some social-media environments (^19^, but see^21^). Although having even less false information would limit statistical power, the effectiveness of nudges may be reduced in these circumstances. Nonetheless, the current pattern of results suggests that nudge-based interventions are unlikely to negatively impact engagement behavior even in information environments with negligible misinformation. Furthermore, and crucially, within social-media environments that do contain a small but non-negligible proportion of misinformation, nudges appear to lead to beneficial changes in engagement behavior.

Cumulatively, the current findings suggest that even when misinformation volume is low, large-scale nudge-based interventions are likely to lead to an immediate, small improvement in engagement, particularly sharing, behavior. Although additional research is required to assess the enduring effectiveness of the intervention over time and misinformation types, these findings provide theoretical support for the limited-attention utility model^13^, and suggest nudge-based interventions are generally well suited to the online social-media environments they are intended to be implemented in.

### Supplementary Information

Supplementary Information 1.

Supplementary Information 2.

Supplementary Information 3.

Supplementary Information 4.

Supplementary Information 5.

## Data Availability

Data and the R script, Rmarkdown file, Supplementary information and materials from the empirical studies are available at on the Open Science Framework at https://osf.io/nztuk/. The study was preregistered at https://osf.io/ch7n9.
