# Supplementary material for: Nudge-based misinformation interventions are effective in information environments with low misinformation prevalence
Source: Sci Rep. 2024 May 20;14:11495. doi: 10.1038/s41598-024-62286-7 (PMC11106285; doi:10.1038/s41598-024-62286-7)
Supplement: Supplementary file 1 — Supplementary Information 1. [file 41598_2024_62286_MOESM1_ESM.pdf]

Supplementary materials for *"Nudge-Based Misinformation Interventions are Effective in Information Environments with Low Misinformation Prevalence"*

Supplement A – Material Validation Studies

Two material-validation pilot surveys were administered through Qualtrics survey software (Qualtrics, Provo, UT) to obtain ratings for an initial pool of 120 claims (60 false, 60 true). In the first material-validation survey, 52 participants (26 male, 25 female, one non-binary; M_{age} = 41.10, SD_{age} = 12.72) rated all 120 claims on (1) currentness (*"Could this be a current headline?")* on a 5-point scale from 0 ("Definitely no") to 4 ("Definitely yes"), and (2) shareability ("*How likely would it be for people to share this headline online?"*) on a 7-point scale from 0 ("Very unlikely") to 6 ("Very likely"). In the second material validation survey, a separate sample of 50 participants (24 male, 25 female, one non-binary; M_{age} = 38.96, SD_{age} = 12.69) rated all 120 claims on (1) believability (*"How believable is the headline?"*) on an 11-point scale from 0 ("Not believable at all") to 10 ("Very believable"), and (2) political leaning (*"Assuming the above headline is entirely accurate, how favorable would it be to Democrats versus Republicans?"*) on a 7-point scale from 0 ("Very favorable to Democrats") to 6 ("Very favorable to Republicans).

Claim Selection

Ratings for all 60 false headlines are displayed in Table A1, and ratings for all 60 true headlines are displayed in Table A2. To select the set of headlines used in the main study, headlines were excluded if they were highly politically congruent with either left-wing or right-wing people (<2.5 or >4 on the 0-6 political leaning scale) or if they were unlikely to be current (<1.3 on the 0-4 current scale). One additional false headline (*"Asteroid bigger than Big Ben to pass Earth's atmosphere in new year"*) was excluded due to its veracity being somewhat ambiguous. This resulted in a total of 41 true headlines and 38 false headlines. To obtain exactly 40 true and 40 false headlines, an additional true headline (*"Jeffrey Epstein's*

estate reaches a $105 million settlement with the U.S. Virgin Islands") was removed due to its similarity to another true headline (*"Financier buys Jeffrey Epstein's private islands, with plans to create a resort"*). Additionally, one false headline (*In Australia, a "17% increase in heart attack deaths" in the first eight months of 2022 is linked to COVID-19 vaccination*) with a political leaning mean 4.12, and a second false headline (*McDonald's is the world's largest purchaser of cow eyeballs*) with a currentness mean rating of 1.00 were included as filler false headlines in the 50% misinformation conditions. See Tables A3 for summary statistics for the pilot ratings of the 40 true and false headlines, and the 10 target false headlines, used in the main study. The headline used for the accuracy nudge is displayed in Figure A1.

Figure A1

False Headline Used for Accuracy Prompt

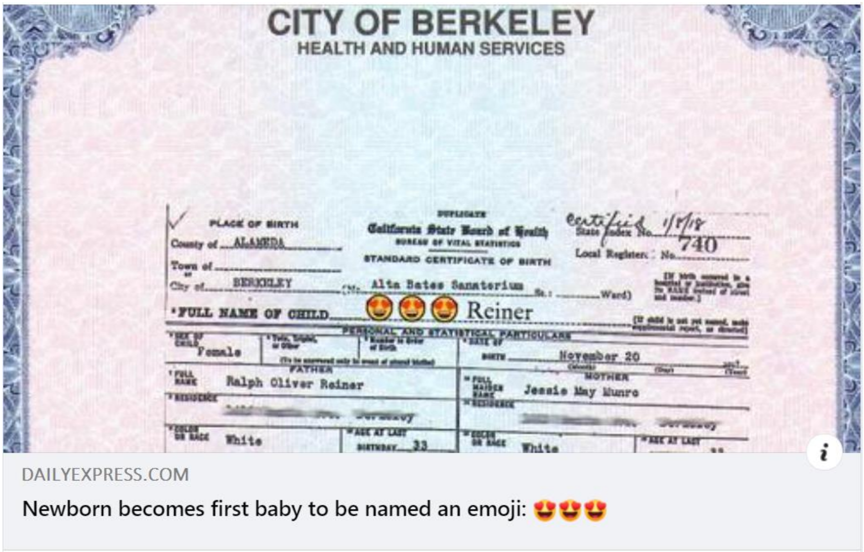

Note. Image taken from https://prettycoolsite.com/california-newborn-baby-name-emoji-smiley-heart/

Table A1

Currentness, shareability, believability, and political leaning ratings of false headlines

False Claims	Currentness		Shareability		Believability		Political Leaning		Exclusion Criteria
	M	*SD*	*M*	*SD*	*M*	*SD*	*M*	*SD*	
^Joe Biden lookalike tried to get free meals at McDonald's while impersonating president	1.85	1.32	3.60	2.11	5.08	3.19	3.60	1.23	
Bill Clinton brought his teenage daughter to Epstein Island	1.73	1.44	3.69	2.21	4.68	3.24	4.12	1.60	Pol > 4.00
Bill Gates faces trial in India for illegally testing tribal children with vaccines	1.33	1.42	3.40	2.25	2.50	3.21	4.28	1.39	Pol > 4.00
Report: Vaccine related deaths may rival recorded Covid-19 deaths	1.96	1.39	3.71	2.07	3.16	3.62	4.36	1.50	Pol > 4.00
Court orders Tiger Woods to take 137 paternity tests	1.27	1.22	3.35	2.11	2.66	3.21	3.00	0.57	Current < 1.30
^Death row inmate demands to be served a live cat as his last meal	1.46	1.23	3.46	2.32	3.66	3.40	3.24	0.85	
^Evidence surfaces that the FBI planned and executed January 6 Capitol riot	1.85	1.47	4.56	1.76	3.58	3.78	3.78	1.90	
*Male stripper shot 5 times after showing up at a gang hideout by mistake in a cop costume	2.23	1.13	3.81	1.86	4.92	3.16	3.14	0.93	
*New Jersey brother and sister allowed to marry after 10-year-long court battle	1.85	1.26	3.87	1.94	4.40	2.81	3.08	1.07	

White nationalists granted permit for mock hanging outside Afro-American museum	1.23	1.29	3.67	2.28	2.88	3.17	3.00	1.50	Current < 1.30
BREAKING: Hillary Clinton found dead under suspicious circumstances	1.08	1.48	3.52	2.47	2.32	3.30	4.08	1.55	Pol > 4.00, Current < 1.30
^Delta force seizes deep state stronghold	1.44	1.11	2.88	1.96	3.30	3.32	3.68	1.36	
*WATCH: Alex Jones physically stops vehicle after witnessing migrant children being stuffed into the luggage compartment	1.92	1.45	3.67	2.08	2.72	2.96	3.86	1.34	
Astra-Zeneca manufacture "COVID-19 vaccine" in July of 2018 before the disease was even discovered or named	1.90	1.36	3.98	2.08	3.18	3.30	4.12	1.30	Pol > 4.00
WOW! Biden caught fake driving -- someone else is steering vehicle -- it was all a stunt!	1.44	1.33	3.21	2.08	2.68	2.96	4.76	1.29	Pol > 4.00
^Joe Biden signs executive order to change "White House" to something less racially offensive	1.38	1.55	3.56	2.44	2.62	3.19	2.88	2.00	
UK Pathologist warns spike proteins in vaccines will cause all men to lose their reproductive capacity	1.50	1.31	3.31	2.11	2.94	3.15	4.10	1.22	Pol > 4.00
U.N. health experts admit toxic vaccine ingredients are harming children worldwide	2.21	1.30	4.27	1.88	3.90	3.29	4.22	1.47	Pol > 4.00
Ilhan Omar spits on the tomb of the unknown soldier	1.58	1.33	3.60	2.06	2.96	3.12	4.10	1.40	Pol > 4.00

*Portland bans urinals in public buildings - out of respect to the city's "shared values"	1.90	1.29	3.27	1.97	4.18	3.42	3.32	1.39	
Leaked visitor logs reveal Schiff's 78 visits to Epstein Isle	2.10	1.21	3.60	1.97	5.62	3.12	3.70	1.66	
Military Tribunal sentences Sonia Sotomayor to death	1.23	1.29	2.87	2.11	2.38	3.08	3.76	1.25	Current < 1.30
^National guard terrorizes New Mexico school children	1.83	1.15	3.85	1.98	3.80	3.17	2.68	1.39	
*Putin beheads bioweapon engineers in Ukraine	2.02	1.35	3.85	2.01	4.24	3.44	3.08	1.44	
^Putin bombs Biden-owned villa in Ukraine	1.42	1.24	3.81	2.17	2.52	3.08	3.94	1.58	
Chocolate milk is made from “regular” milk rejected for containing too much cow’s blood.	1.29	1.38	2.81	2.16	1.90	2.68	3.20	0.97	Current < 1.30
*More than 60 people died in Mozambique because they consumed beer that was poisoned with crocodile bile.	1.85	1.18	3.23	1.84	4.14	3.34	2.98	0.62	
^Experts Claim 'Super Volcano' in Yellowstone Could Erupt Within Weeks	2.06	1.23	3.71	1.92	5.04	3.40	2.92	0.70	
*Michigan house passes human microchipping legislation	1.90	1.32	3.71	2.12	3.64	3.36	3.38	1.55	
^Maine House Democrats vote to allow female genital mutilation	1.62	1.44	3.75	2.06	2.60	3.16	3.80	1.76	

^12 white female bodies in garage freezer tagged "Black Lives Matter"	1.63	1.31	3.87	2.12	2.18	2.87	3.96	1.69	
Billionaire founder of Corona beer brewery makes EVERYONE in his village a MILLIONAIRE in his will	1.23	1.21	3.13	2.18	2.58	3.04	2.88	1.02	Current < 1.30
^Cher had her lowest pair of ribs surgically removed to achieve an ultra-small waist.	1.81	1.31	3.27	2.06	3.98	3.21	3.00	0.64	
^Adding acetone to gasoline improves gas mileage	1.63	1.10	2.81	1.85	2.94	2.72	3.00	0.70	
JUST IN: TRUMP paid woman for SEX in Cancun	2.48	1.41	4.48	1.97	6.18	3.25	1.46	1.57	Pol < 2.50
^McDonald's is the world's largest purchaser of cow eyeballs	1.00	1.12	3.02	2.23	2.14	3.18	3.26	0.80	Current < 1.30
~California new born becomes the first child with an emoji for a name	2.31	1.28	3.87	2.14	5.70	3.63	3.00	1.07	Used as prompt
^Woman arrested for defecating on boss' desk after winning the lottery	2.10	1.16	3.87	2.08	5.86	3.33	3.00	0.53	
^Milk-Bone dog treats cause cancer in one of every two dogs that eat them.	1.83	1.31	3.75	2.13	3.20	3.17	3.06	0.47	
Nancy Pelosi's son arrested for murder	1.52	1.39	4.06	2.26	3.24	3.35	4.96	1.24	Pol > 4.00
The Obama Foundation stored classified presidential records in an empty furniture warehouse.	2.06	1.41	4.00	2.07	3.36	3.21	4.76	1.38	Pol > 4.00

Asteroid bigger than Big Ben to pass Earth's atmosphere in new year	2.33	1.10	3.63	1.89	6.36	2.80	2.86	0.57	*Excluded due to veracity ambiguity*
*Why a whiff of rosemary DOES help you remember: Sniffing herb can increase memory by 75%	2.17	0.96	3.38	1.86	4.36	3.06	2.96	0.70	
*Schools in the UK no longer teach about the Holocaust for fear of offending Muslim students.	1.85	1.39	3.69	2.04	3.88	3.32	3.26	1.24	
*Trump's old science advisor denies Apollo moon landings ever happened	1.98	1.26	3.54	2.02	4.70	3.67	2.56	1.93	
^Saudi Arabia: Panel of scientists admits women are mammals, yet "not human"	1.44	1.32	3.21	2.45	2.52	2.85	3.30	1.28	
^BREAKING ALERT! KIM JONG-UN JUST ORDERED THE EVACUATION OF PYONGYANG	1.81	1.25	3.60	1.98	3.32	3.13	3.24	1.02	
^In a simulation, an AI-enabled drone operated by the U.S. Air Force killed its operators and “started taking out communication towers.”	1.81	1.30	3.77	1.99	3.74	3.13	3.22	1.17	
JUST IN: A MASSIVE CARAVAN of illegal aliens is forcing its way into the US!	2.60	1.33	4.37	2.09	3.96	3.82	4.54	1.47	Pol > 4.00
^Babysitter on crystal meth eats 3-month-old toddler	1.31	1.28	3.96	2.08	2.56	2.98	3.14	0.86	

^Elon Musk is offering people the opportunity to make $1 million "in 90 days while sleeping in."	1.52	1.42	3.58	2.28	3.14	3.06	3.42	0.93	
^Pouring soda on raw pork will cause parasites to emerge	1.60	1.14	3.58	1.93	3.52	3.49	3.06	0.51	
^Vladimir Putin orders destruction of all COVID-19 vaccines in Russia.	1.58	1.24	3.67	2.08	3.52	2.96	3.84	1.20	
^In Australia, a "17% increase in heart attack deaths" in the first eight months of 2022 is linked to COVID-19 vaccination	2.38	1.29	4.27	1.91	4.26	3.56	4.12	1.39	Pol > 4.00
^Clinton aide found dead, tied to tree in apparent murder	1.54	1.32	3.48	2.11	4.18	3.63	4.00	1.34	
^The dog dewormer fenbendazole can cure cancer in humans	1.56	1.24	3.42	1.96	2.82	3.29	3.14	0.70	
^Elon Musk To Buy Disney World	1.81	1.57	3.83	2.30	3.36	3.57	3.44	0.97	
^Target Is Offering 'Tuck-Friendly' Bathing Suits for Kids	2.50	1.34	3.62	2.34	5.78	3.52	3.14	1.75	
^Elon Musk Creates Cannabidiol Gummies that Reverse Dementia	1.33	1.38	3.83	1.93	2.14	2.96	3.16	0.98	
^'Transgender' Toddlers as Young as 2 Undergoing Mutilation/Sterilization by NC Medical System	1.73	1.51	3.77	2.38	4.04	3.36	3.80	1.69	

Note. *Denotes target false claims, ^denotes filler false claims, and ~denotes prompt headline used in the main study

Table A2

Currentness, shareability, believability, and political leaning ratings of true headlines

Claims	Currentness		Shareability		Believability		Political Leaning		Exclusion Criteria
	M	*SD*	*M*	*SD*	*M*	*SD*	*M*	*SD*	
$14 million jury award for protesters harmed in Denver protests could resonate around U.S.	2.46	1.06	3.60	1.72	6.50	2.65	2.24	1.35	Pol < 2.50
Alabama man with Molotov cocktails, guns on Jan. 6 gets 46-month sentence	3.04	0.86	3.92	1.69	7.42	2.67	2.00	1.51	Pol < 2.50
Florida man sentenced to 18 months in prison for Pelosi, AOC death threats	2.94	0.98	3.92	1.93	7.10	2.53	2.22	1.63	Pol < 2.50
*Four U.S. senators cite Microsoft-Activision deal concern in Federal Trade Commission letter	2.62	0.95	3.02	1.74	7.16	2.27	2.94	0.98	
Harris hails $4.7 billion corporate pledge for D.C.-area minority firms	2.65	0.93	3.21	1.70	6.44	2.43	1.88	1.26	Pol < 2.50
*House passes bill approving Capitol statues for Ginsburg, O'Connor	2.02	1.16	2.83	1.97	5.82	3.01	2.70	1.62	
*IRS, Department of Homeland Security contracted firm that sells location data harvested from dating apps	2.44	1.00	3.58	2.04	5.30	3.26	3.52	0.95	
*Mafia hitman escapes from federal custody in Florida	2.50	0.96	3.73	1.75	6.18	2.86	3.18	0.94	

Pentagon links leadership failures to violence, harassment, at military bases	2.42	1.05	2.90	1.77	6.20	2.78	2.48	1.15	Pol < 2.50
*Romney suggests cutting retirement benefits for younger Americans	2.54	1.13	3.48	1.97	7.00	2.29	3.66	1.79	
*Senators call for investigations into Hertz following CBS News reports of hundreds of customers allegedly being falsely arrested	2.10	1.16	3.19	1.92	5.16	3.32	3.02	0.68	
*Soldiers among those charged with interstate gun-trafficking	2.40	1.18	3.42	1.96	6.74	2.59	2.50	1.23	
Supreme court to consider California rules regarding treatment of pigs	2.12	1.11	2.71	1.72	5.92	2.82	2.48	1.11	Pol < 2.50
*This isn't about attention: Anti-death penalty activist marries Oklahoma death row inmate	2.35	1.23	3.40	2.05	6.06	3.01	2.56	1.09	
US college grad helps rescue Ukrainian refugees	2.92	0.79	3.60	1.72	7.72	2.01	2.34	1.29	Pol < 2.50
While red states restrict abortion, blue states are voting to protect access	3.19	1.14	4.31	1.80	7.68	2.78	2.10	1.58	Pol < 2.50
U.S. passports now offer 'X' gender marker	2.62	1.27	3.90	2.04	5.88	3.42	2.38	1.72	Pol < 2.50
*Woman who had ovary frozen in childhood gives birth	1.98	1.20	3.19	1.99	4.78	3.18	2.82	0.63	
*New Yale Research: Coronavirus can 'hijack' brain cells to replicate itself	1.90	1.24	3.73	1.97	3.36	3.34	3.32	1.27	

*$400 million of ransomware revenue went to Russia-linked groups in 2021	2.60	1.03	3.60	1.71	6.66	3.00	2.90	1.34	
*Climate change: Big banks pump cash into coal industry in spite of net zero pledges, research finds	2.62	0.97	3.48	1.65	5.78	2.70	3.12	1.59	
*Earth has an extra companion, a Trojan asteroid that may hang around for 4,000 years	2.31	1.06	2.94	1.95	5.14	2.96	2.96	0.64	
*Ethiopian schoolboy generates electricity from biogas for his village	2.25	1.06	2.92	1.82	6.46	2.67	2.82	1.10	
*Exceedingly rare fossil of giant flying reptile discovered on Scottish island	2.38	1.09	3.23	1.79	6.08	3.05	2.88	0.72	
First woman to be 'cured' of HIV with stem cell blood cancer treatment	2.50	1.16	4.10	1.94	6.18	3.23	2.48	1.09	Pol < 2.50
*Supermassive black hole found hiding in ring of cosmic dust	2.50	1.06	3.25	1.79	6.28	3.01	2.72	0.73	
Zoo cryogenically freezes genetic samples in a battle to save dozens of species from extinction	2.73	1.01	3.44	1.69	7.20	2.39	2.44	1.07	Pol < 2.50
At Trump's big-city hotels, business dropped as his political star rose, internal documents show	2.71	0.98	3.73	1.78	6.16	2.57	2.08	1.48	Pol < 2.50
*Americans are using Apple AirTags to track loved ones with dementia, report says	2.96	1.01	3.92	1.72	7.40	2.56	2.90	0.71	

*Feeling stressed? New research out of the UK suggests it's very likely your dog can smell it	2.94	0.83	3.90	1.67	7.18	3.13	2.94	0.59	
*SYDNEY, AUSTRALIA: School children suffer serious burns after outdoor science experiment goes wrong	2.44	1.02	3.48	1.75	6.60	2.53	3.14	0.78	
A man who burned a cross to intimidate his Black neighbors pleads guilty to hate crime	2.65	1.15	4.04	1.88	8.20	2.18	2.12	1.64	Pol < 2.50
*Northern long-eared bat, devastated by a fungus, is now listed as endangered	2.67	0.86	2.60	1.73	7.50	2.33	2.86	0.76	
Jeffrey Epstein's estate reaches a $105 million settlement with the U.S. Virgin Islands	2.50	1.00	3.60	1.84	6.52	3.00	3.04	1.03	*Removed due to similarity*
*US man, 79, beaten to death in Mexico while delivering donations to the poor	2.63	1.03	3.88	1.75	6.36	3.06	3.62	1.23	
*Canada expels Chinese diplomat for alleged intimidation of lawmaker	2.37	1.01	3.02	1.99	6.22	2.44	2.90	1.22	
*N.Y. Rep. George Santos pleads not guilty to federal fraud charges	2.90	1.12	3.67	1.90	7.52	2.78	2.92	1.44	
A California panel has called for billions in reparations for descendants of slaves	2.65	1.41	4.04	2.03	7.06	2.76	1.92	1.65	Pol < 2.50
George Santos confessed to stealing checks in Brazil in a deal to drop charges	2.38	1.14	3.73	1.79	5.72	3.01	2.44	1.54	Pol < 2.50
*DNA evidence reveals family man in Australia was teenage killer who escaped Nebraska jail	2.29	1.21	3.52	2.00	5.80	3.13	3.18	0.63	

*The Supreme Court outlawed split juries, but hundreds remain in prison anyway	2.04	1.24	3.15	1.90	5.18	3.26	3.02	1.24	
Chief Standing Bear, Native American civil rights icon, is honored on a postal stamp	2.62	0.93	2.65	1.70	7.60	2.09	2.22	1.17	Pol < 2.50
*Jury finds Lori Vallow Daybell guilty of murdering 2 of her children	2.69	1.15	4.19	1.53	7.14	2.91	3.14	0.70	
*Norfolk Southern railcars derail in Pennsylvania, but with no hazardous chemicals	2.56	1.13	2.92	2.02	6.54	2.88	3.02	0.82	
More gay and bisexual men will now be able to donate blood under finalized FDA rules	2.75	1.10	3.58	1.84	7.44	2.67	1.94	1.48	Pol < 2.50
*Peloton is recalling nearly 2.2 million bikes due to a seat hazard	2.85	1.04	3.77	1.86	7.16	2.54	2.98	0.47	
*8 people killed after vehicle drives into group at bus stop in Texas border city	3.06	0.85	4.42	1.60	8.12	1.88	3.24	1.06	
*Financier buys Jeffrey Epstein's private islands, with plans to create a resort	2.38	1.17	3.48	2.02	6.04	3.06	3.42	1.13	
*Dozens are reported dead from a fire deep in a gold mine in Peru	2.60	0.96	3.37	1.69	7.26	2.46	3.02	0.43	
*Jury finds that Ed Sheeran didn't copy Marvin Gaye classic 'Let's Get It On'	3.02	1.09	3.90	1.84	7.8	2.57	2.86	0.53	
*Woman survives five days in Australian wilderness with wine, lollipops	2.19	1.10	4.12	1.70	5.70	2.87	3.02	0.55	

*Man arrested after stealing 5-ton military vehicle and leading police on highway chase in Maryland	2.54	1.04	4.25	1.74	6.70	2.42	3.02	0.82	
*Colorado mountain lion euthanized after swatting an 11-year-old girl in ‘rare’ attack	2.52	1.00	4.06	1.76	7.46	2.36	3.14	0.53	
CDC sets first target for indoor air ventilation to prevent spread of Covid-19	2.38	1.07	3.33	1.68	5.82	2.77	2.36	1.10	Pol < 2.50
*FDA greenlights a new type of drug for menopausal hot flashes	2.69	0.85	3.29	1.74	7.28	2.57	2.76	0.85	
*A recalled Gerber powdered baby formula was distributed to some US retailers after the initial recall notice, company says	2.71	1.13	4.17	1.69	7.00	2.74	3.12	0.69	
*Illinois' 'assault weapons' ban could be on the chopping block — at least temporarily	2.60	1.05	3.83	1.53	6.88	2.26	3.54	1.66	
Biden tells Howard grads 'White supremacy' is the 'most dangerous terrorist threat' to the United States	2.48	1.31	4.21	1.81	7.22	2.49	1.90	1.64	Pol < 2.50
*North Carolina's governor vetoed a 12-week abortion ban, setting up an override fight	3.00	0.79	4.17	1.58	7.00	2.60	2.50	1.81	
*A Texas woman was killed by her boyfriend after getting an abortion, police say	3.00	0.82	4.35	1.61	7.76	1.89	2.96	1.62	

Note. * Denotes true headlines used in the main study

Table A3

Average ratings for true and false headlines used in the main study

	Current (0-4)		Shareability (0-6)		Believability (0-10)		Political Leaning (0-6)	
	M	*SD*	*M*	*SD*	*M*	*SD*	*M*	*SD*
False Headlines (10 target headlines)	1.97	0.14	3.60	0.23	4.12	0.61	3.16	0.34
False Headlines (All 40 headlines)	1.76	0.31	3.62	0.33	3.71	0.99	3.33	0.39
True Headlines (All 40 Headlines)	2.53	0.30	3.56	0.46	6.49	0.97	3.02	0.28

Table A4

Engagement Frequency (% option was chosen) for true and false headlines across the misinformation proportion and nudge conditions.

			Frequency (%)			
Post Veracity	Nudge Condition	Misinformation Proportion	No Engagement	Like only	Share only	Like and share
False	No Nudge	12.5%	67.83	11.45	9.77	10.95
		20%	60.28	11.36	14.32	14.04
		50%	69.20	10.81	9.74	10.25
	Nudge	12.5%	72.20	11.61	8.21	7.98
		20%	65.92	12.96	9.96	11.17
		50%	70.46	9.53	8.83	11.19
True	No Nudge	12.5%	59.06	17.02	10.48	13.44
		20%	51.54	17.31	13.21	17.94
		50%	56.73	14.87	11.49	16.90
	Nudge	12.5%	59.26	16.98	9.59	14.17
		20%	51.31	19.48	11.51	17.69
		50%	53.08	15.46	11.76	19.70

Table A5

Engagement (liking, sharing) with True and False Posts at the Item Level (F = False, T = True). Images can be found at https://osf.io/nztuk/ under the Misinformation Game templates tab

	Like Frequency		Share Frequency	
Claim	M	SD	M	SD
F1. Joe Biden lookalike tried to get free meals at McDonald's while impersonating president	0.35	0.48	0.27	0.44
F2. Death row inmate demands to be served a live cat as his last meal	0.15	0.36	0.19	0.39
F3. Evidence surfaces that the FBI planned and executed January 6 Capitol riot	0.20	0.40	0.18	0.39
F4. Male stripper shot 5 times after showing up at a gang hideout by mistake in a cop costume (Target)	0.23	0.42	0.23	0.42
F5. New Jersey brother and sister allowed to marry after 10-year-long court battle (Target)	0.12	0.33	0.18	0.38
F6. Delta force seizes deep state stronghold	0.19	0.39	0.10	0.30
F7. WATCH: Alex Jones physically stops vehicle after witnessing migrant children being stuffed into the luggage compartment (Target)	0.29	0.45	0.20	0.40
F8. Joe Biden signs executive order to change "White House" to something less racially offensive	0.19	0.39	0.17	0.38
F9. Portland bans urinals in public buildings - out of respect to the city's "shared values" (Target)	0.17	0.38	0.16	0.37
F10. Leaked visitor logs reveal Schiff's 78 visits to Epstein Isle	0.20	0.40	0.19	0.39
F11. National guard terrorizes New Mexico school children	0.16	0.37	0.20	0.40
F12. Putin beheads bioweapon engineers in Ukraine (Target)	0.14	0.35	0.16	0.37
F13. Putin bombs Biden-owned villa in Ukraine	0.15	0.36	0.15	0.36

F14. More than 60 people died in Mozambique because they consumed beer that was poisoned with crocodile bile (Target)	0.22	0.41	0.22	0.41
F15. Experts Claim 'Super Volcano' in Yellowstone Could Erupt Within Weeks	0.31	0.46	0.30	0.46
F16. Michigan house passes human microchipping legislation (Target)	0.18	0.38	0.24	0.43
F17. Maine House Democrats vote to allow female genital mutilation	0.11	0.31	0.15	0.36
F18. 12 white female bodies in garage freezer tagged "Black Lives Matter"	0.15	0.36	0.19	0.40
F19. Cher had her lowest pair of ribs surgically removed to achieve an ultra-small waist	0.17	0.38	0.15	0.36
F20. Adding acetone to gasoline improves gas mileage	0.26	0.44	0.19	0.39
F21. McDonald's is the world's largest purchaser of cow eyeballs	0.17	0.38	0.19	0.39
F22. Woman arrested for defecating on boss' desk after winning the lottery	0.39	0.49	0.32	0.47
F23. Milk-Bone dog treats cause cancer in one of every two dogs that eat them	0.22	0.42	0.29	0.45
F24. In Australia, a “17% increase in heart attack deaths” in the first eight months of 2022 is linked to COVID-19 vaccination	0.22	0.42	0.22	0.41
F25. Why a whiff of rosemary DOES help you remember: Sniffing herb can increase memory by 75% (Target)	0.52	0.50	0.34	0.47
F26. Schools in the UK no longer teach about the Holocaust for fear of offending Muslim students (Target)	0.16	0.37	0.22	0.41
F27. Trump's old science advisor denies Apollo moon landings ever happened (Target)	0.20	0.40	0.20	0.40
F28. Saudi Arabia: Panel of scientists admits women are mammals, yet "not human"	0.11	0.32	0.17	0.38
F29. BREAKING ALERT! KIM JONG-UN JUST ORDERED THE EVACUATION OF PYONGYANG	0.16	0.36	0.14	0.35

F30. In a simulation, an AI-enabled drone operated by the U.S. Air Force killed its operators and “started taking out communication towers”	0.21	0.41	0.23	0.42
F31. Babysitter on crystal meth eats 3-month-old toddler	0.15	0.36	0.19	0.39
F32. Elon Musk is offering people the opportunity to make $1 million “in 90 days while sleeping in”	0.40	0.49	0.27	0.45
F33. Pouring soda on raw pork will cause parasites to emerge	0.20	0.40	0.21	0.41
F34. Vladimir Putin orders destruction of all COVID-19 vaccines in Russia	0.19	0.39	0.17	0.38
F35. Clinton aide found dead, tied to tree in apparent murder	0.17	0.37	0.19	0.40
F36. The dog dewormer fenbendazole can cure cancer in humans	0.22	0.41	0.16	0.37
F37. Elon Musk To Buy Disney World	0.24	0.43	0.21	0.41
F38. Target Is Offering 'Tuck-Friendly' Bathing Suits for Kids	0.21	0.41	0.20	0.40
F39. Elon Musk Creates Cannabidiol Gummies that Reverse Dementia	0.27	0.45	0.18	0.39
F40. ‘Transgender’ Toddlers as Young as 2 Undergoing Mutilation/Sterilization by NC Medical System	0.11	0.32	0.16	0.36
T1. Four U.S. senators cite Microsoft-Activision deal concern in Federal Trade Commission letter	0.30	0.46	0.22	0.41
T2. House passes bill approving Capitol statues for Ginsburg, O'Connor	0.35	0.48	0.21	0.41
T3. IRS, Department of Homeland Security contracted firm that sells location data harvested from dating apps	0.27	0.44	0.32	0.47
T4. Mafia hitman escapes from federal custody in Florida	0.26	0.44	0.26	0.44
T5. Romney suggests cutting retirement benefits for younger Americans	0.17	0.38	0.29	0.45
T6. Senators call for investigations into Hertz following CBS News reports of hundreds of customers allegedly being falsely arrested	0.29	0.46	0.22	0.42

T7. Soldiers among those charged with interstate gun-trafficking	0.23	0.42	0.21	0.41
T8. This isn't about attention: Anti-death penalty activist marries Oklahoma death row inmate	0.21	0.41	0.17	0.38
T9. Woman who had ovary frozen in childhood gives birth	0.43	0.49	0.24	0.42
T10. New Yale Research: Coronavirus can 'hijack' brain cells to replicate itself	0.29	0.46	0.32	0.47
T11. $400 million of ransomware revenue went to Russia-linked groups in 2021	0.26	0.44	0.26	0.44
T12. Climate change: Big banks pump cash into coal industry in spite of net zero pledges, research finds	0.30	0.46	0.31	0.46
T13. Earth has an extra companion, a Trojan asteroid that may hang around for 4,000 years	0.50	0.50	0.34	0.47
T14. Ethiopian schoolboy generates electricity from biogas for his village	0.59	0.49	0.39	0.49
T15. Exceedingly rare fossil of giant flying reptile discovered on Scottish island	0.53	0.50	0.34	0.47
T16. Supermassive black hole found hiding in ring of cosmic dust	0.57	0.50	0.39	0.49
T17. Americans are using Apple AirTags to track loved ones with dementia, report says	0.60	0.49	0.41	0.49
T18. Feeling stressed? New research out of the UK suggests it's very likely your dog can smell it	0.63	0.48	0.42	0.49
T19. SYDNEY, AUSTRALIA: School children suffer serious burns after outdoor science experiment goes wrong	0.23	0.42	0.26	0.44
T20. Northern long-eared bat, devastated by a fungus, is now listed as endangered	0.33	0.47	0.35	0.48
T21. US man, 79, beaten to death in Mexico while delivering donations to the poor	0.21	0.41	0.26	0.44
T22. Canada expels Chinese diplomat for alleged intimidation of lawmaker	0.24	0.43	0.15	0.36
T23. N.Y. Rep. George Santos pleads not guilty to federal fraud charges	0.21	0.41	0.19	0.39

T24. DNA evidence reveals family man in Australia was teenage killer who escaped Nebraska jail	0.30	0.46	0.26	0.44
T25. The Supreme Court outlawed split juries, but hundreds remain in prison anyway	0.24	0.43	0.23	0.42
T26. Jury finds Lori Vallow Daybell guilty of murdering 2 of her children	0.36	0.48	0.28	0.45
T27. Norfolk Southern railcars derail in Pennsylvania, but with no hazardous chemicals	0.27	0.45	0.24	0.43
T28. Peloton is recalling nearly 2.2 million bikes due to a seat hazard	0.32	0.47	0.36	0.48
T29. 8 people killed after vehicle drives into group at bus stop in Texas border city	0.21	0.41	0.26	0.44
T30. Financier buys Jeffrey Epstein's private islands, with plans to create a resort	0.26	0.44	0.23	0.42
T31. Dozens are reported dead from a fire deep in a gold mine in Peru	0.20	0.40	0.26	0.44
T32. Jury finds that Ed Sheeran didn't copy Marvin Gaye classic 'Let's Get It On'	0.47	0.50	0.24	0.43
T33. Woman survives five days in Australian wilderness with wine, lollipops	0.56	0.50	0.36	0.48
T34. A recalled Gerber powdered baby formula was distributed to some US retailers after the initial recall notice, company says	0.25	0.43	0.37	0.48
T35. Colorado mountain lion euthanized after swatting an 11-year-old girl in ‘rare’ attack	0.23	0.42	0.25	0.43
T36. FDA greenlights a new type of drug for menopausal hot flashes	0.50	0.50	0.30	0.46
T37. Man arrested after stealing 5-ton military vehicle and leading police on highway chase in Maryland	0.36	0.48	0.28	0.45
T38. Illinois' 'assault weapons' ban could be on the chopping block — at least temporarily	0.28	0.45	0.25	0.43
T39. North Carolina's governor vetoed a 12-week abortion ban, setting up an override fight	0.38	0.49	0.29	0.45
T40. A Texas woman was killed by her boyfriend after getting an abortion, police say	0.19	0.39	0.24	0.43

Table A6

Engagement (liking, sharing) with social posts in the 12.5% misinformation condition. Images can be found at https://osf.io/nztuk/ under the Misinformation Game templates tab

	Like Frequency		Share Frequency	
Claim	M	SD	M	SD
1. Got a new recipe for a homemade apple pie. Can't wait to try it - the kitchen smells amazing!	.69	.46	.25	.43
2. Waking up to a beautiful day and a cup of coffee. Have a great day, everyone!	.69	.46	.19	.39
3. Just finished my morning jog. Feels good to stay active and keep those joints moving.	.64	.48	.13	.34
4. Travel bug bites again! Just booked my next adventure to Bali. So excited!! 🌴 ✈️	.61	.49	.15	.36
5. Can't resist a good sale! Scored some amazing deals at my favorite thrift shop. Time to revamp my wardrobe!	.57	.50	.15	.36
6. Took a break from technology and went camping last weekend. So good to unwind by the campfire.	.74	.44	.20	.40
7. For Sale: Brand new iPhone 12 Pro Max! Unlocked and in pristine condition. Comes with original box and accessories. Asking $600. Comment or PM if interested!	.23	.42	.19	.39
8. Affordable used bike! It's a reliable bike in good condition, selling due to upgrade. $60 ONO, DM if interested	.39	.49	.24	.43
9. Just added a new member to my plant family! Let's hope I can keep it alive!	.65	.48	.14	.35
10. Craving some adventure and new experiences. Planning my next hiking trip to explore breathtaking trails. 🥾 ⛰️	.66	.47	.16	.37
11. Celebrating milestones and accomplishments with gratitude and excitement. Cheers to hard work paying off and new opportunities on the horizon! 🎉 🥂 #Success #NewBeginnings	.56	.50	.12	.33
12. Indulging in some self-care Sunday vibes with a pampering spa session. Taking time to relax, recharge, and prioritize my well-being. 💆 ✨	.58	.49	.17	.38
13. Introducing the newest member of our family! Meet our furry bundle of joy!	.77	.42	.21	.41

14. Enjoying a picturesque evening at the beach, surrounded by good company. Grateful for these priceless moments shared with dear friends.	.68	.47	.18	.39
15. Movie night at home with my favorite snacks and a cozy blanket. Ready to binge-watch my favorite series and unwind.	.63	.48	.11	.32
16. Spent the afternoon exploring The British Museum...so many inspiring exhibits to discover!	.57	.49	.17	.38
17. Cleanse your timeline with this cute puppy photo!	.69	.46	.38	.48
18. Had a culinary adventure at Savoria Bistro tonight! 🌟 The fusion flavors blew my mind, and I'm still savoring every bite. 🍽️	.61	.49	.17	.38
19. Why does it always rain on my days off? It's like the universe has a personal vendetta against my plans. 🌧️ 😒	.41	.49	.14	.35
20. Just when I thought my day couldn't get any worse, I spilled coffee all over my laptop. 😡 😡 😡	.27	.45	.09	.29
21. Some people have zero concept of personal space on public transportation. Excuse me, but your backpack is not entitled to its own seat!	.39	.49	.15	.36
22. Just became a proud homeowner! 🎉 🏡 Excited to start this new chapter and turn my house into a cozy haven. 💖 ✨	.64	.48	.15	.35
23. Too good 😂 😂 😂	.63	.48	.28	.45
24. Looking for advice on buying my kid their first car—what are some key factors to consider and any tips to ensure a safe and reliable choice?	.29	.45	.17	.37
25. Can't believe I finally saw my favorite band in person...I have only been waiting for like EVER!	.62	.49	.14	.35
26. Just returned from an unforgettable trip - Italy, you have stolen a piece of my heart!	.69	.46	.19	.39
27. FREE: Round table, need gone ASAP! Pick up only	.38	.49	.27	.44
28. Proud parent moment! My son scored his first goal in soccer today!! ⚽ 🎉	.63	.48	.12	.32
29. Wishing a very happy birthday to Sarah as she turns 30 today! May this milestone year be filled with joy, laughter, and exciting adventures! 🎉 🎂 🎈	.60	.49	.14	.35
30. My two favorite people at the beach for the first time together! Super cute, first time of many!	.67	.47	.13	.34
