# Supplementary material for: Nudge-based misinformation interventions are effective in information environments with low misinformation prevalence
Source: Sci Rep. 2024 May 20;14:11495. doi: 10.1038/s41598-024-62286-7 (PMC11106285; doi:10.1038/s41598-024-62286-7)
Supplement: Supplementary file 2 — Supplementary Information 2. [file 41598_2024_62286_MOESM2_ESM.pdf]

Supplementary materials for *"Nudge-Based Misinformation Interventions are Effective in Information Environments with Low Misinformation Prevalence"*

Supplement B – Results Output, Pre-registered Main analyses (Engagement Behavior)

We initially pre-registered to assess the impact of misinformation proportion and presence of the nudge intervention on level of engagement, with engagement treated as an ordinal variable with four levels (0 = did not engage, 1 = like, 2 = share, 3 = share and like)[1], using cumulative-link mixed-effects models. Within the pre-registration we specified that the maximal random-effects structure avoiding convergence errors would be used for each analysis. However, these models failed to converge with both participant and post (i.e., item) included as random intercepts, thus leading to a reliance on sub-optimal random effects structures for the main analyses. Thus, as discussed in the main text, we deviated from this analysis plan and assessed sharing and liking behavior as two discrete variables in the main text. However, for completeness, we present the results for engagement behavior here. Given the convergence failures we present both results including participant random intercept, and models including post random intercept, however, models including (only) participant intercept demonstrated a significantly better fit. Please see https://osf.io/gnasr for Rmarkdown containing full results output for engagement behavior.

ANODE results for full model *with* participant random intercepts

Due to the ordinal nature of engagement behavior, analyses were conducted using cumulative-link mixed-effects models using the *clmm* function of the *ordinal* R package [1]; all data visualizations were created using *ggplot2* [2]. Because we were interested in overall main effects and interactions, rather than only main effects and interactions relative to the reference group, we conducted analyses of deviance [3] on the fitted models using the

[1] Within the preregistration we accidentally omitted that (1) participants could both like and sharing the same post, and (2) that doing so would result in a score of 3 (i.e., an additive value of both liking and sharing).

Anova.clm function of the R package *RVAideMemoire* [4]. Post-hoc comparisons were conducted using *emmeans* [5].

Analyses focused on the 10 false and 40 true headlines presented in all conditions[2]. Prior to statistical analysis, the factors nudge (present, absent) and headline veracity (true, false) were centred, and misinformation proportion (12.5%, 20%, 50%) was factor-coded. As per the pre-registration, the primary dependent variable of engagement was coded as an ordinal factor with four levels (0 = no engagement, 1 = like, 2 = share, 3 = like and share).

We assessed the impact of the nudge on engagement with true and false headlines (i.e., engagement discernment) across the conditions with different misinformation proportions (i.e., 12.5% misinformation; 20% misinformation; 50% misinformation). Results of the ANODE are presented in Table B1, and follow-up contrasts are presented in Tables B2 – B4. Average level of engagement across conditions is presented in Figure B1. Note that for completeness (and to provide direct insight into the effect of the nudge intervention isolated to true and false headlines, as per the pre-registration), despite no three-way misinformation proportion × nudge × headline veracity interaction we ran additional contrasts to deconstruct how people engaged with true and false headlines across misinformation proportions *and* nudge conditions (Table B3). Finally, results isolated to each misinformation proportion condition are presented in Tables B5 – B7.

We initially pre-registered to assess the impact of the nudge intervention across misinformation proportion conditions separately for false and true headlines. However, deconstructing the main analysis provided sufficient insight into the direct effects of the nudge intervention on engagement with false and true headlines. Consequently, additional

[2] Although there was no significant difference in engagement with target and filler headlines in the 50% misinformation condition, maintaining filler headlines in the CLMM analyses produced uninterpretable results. Thus, for CLMM analyses assessing engagement behaviour we have only included the 10 target headlines.

analyses isolated to headline type were not run.

Table B1

ANODE Results for Engagement Behavior
Model: Engage ~ Nudge × Misinformation Proportion × Headline Veracity + (1 | Participant)

Fixed Effects	χ^2	*df*	*p*
**Misinformation proportion**	**19.47**	**2**	**<.001**
Nudge	0.16	1	.692
**Headline veracity**	**944.29**	**1**	**<.001**
Misinformation proportion × Nudge	2.10	2	.350
**Misinformation proportion × Headline veracity**	**25.44**	**2**	**<.001**
**Nudge × Headline veracity**	**50.95**	**1**	**<.001**
Misinformation proportion × Nudge × Headline veracity	0.97	2	.616

Figure B1

Average Engagement with True and False News Headlines Across Misinformation-Proportion and Nudge Conditions. Note that 12.5%, 20%, and 50% Refer to the Corresponding Misinformation-Proportion Conditions. Level of Engagement Ranges from No Engagement (0), to Liking (1), Sharing (2), and Both Liking and Sharing (3). Bars Show Condition Means; Error Bars Represent 95% Confidence Intervals; Jittered Dots Represent Individual Participant Means; Violins Provide Distributional Information.

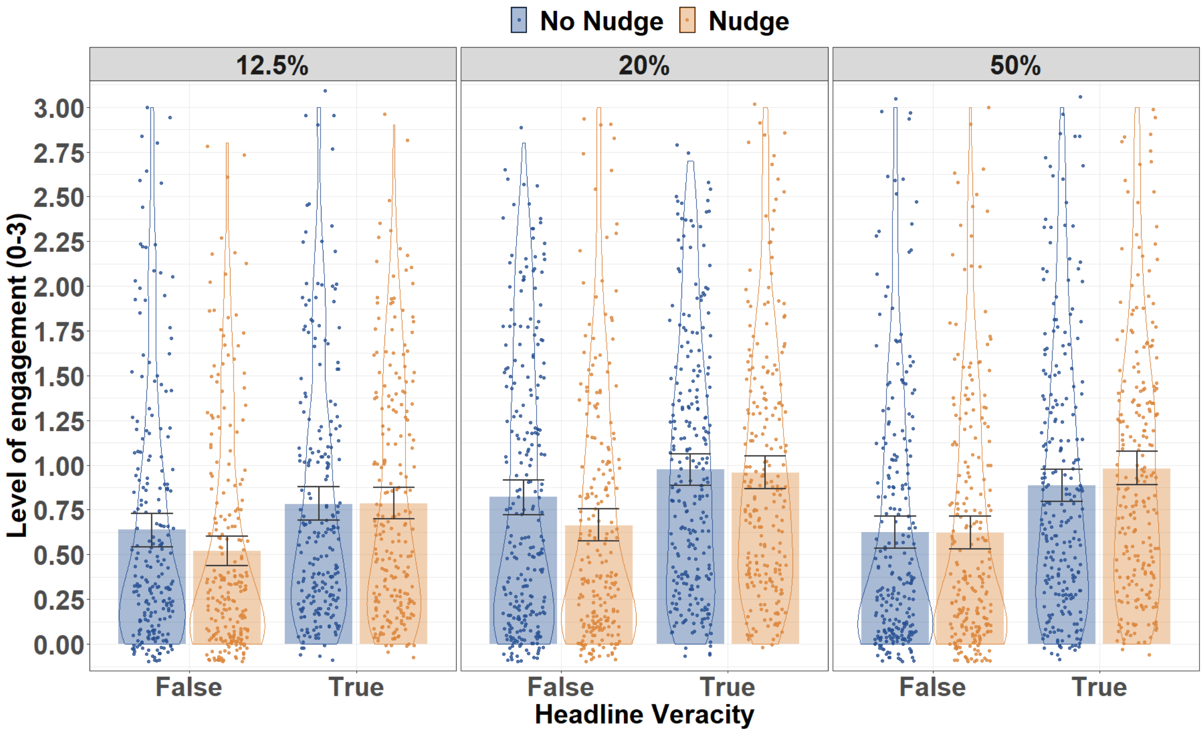

Table B2

Pairwise comparisons assessing impact of misinformation proportion condition on engagement behaviour across true and false headlines

Condition	Misinformation proportion contrast	β	*SE*	*z*	*p*
True	**12.5% – 20%**	**−0.45**	**.11**	**−4.27**	**<.001**
	**12.5% – 50%**	**−0.35**	**.11**	**−3.28**	**.002**
	20% – 50%	0.10	.10	1.00	.319
False	**12.5% – 20%**	**−0.48**	**.12**	**−4.16**	**<.001**
	12.5% – 50%	−0.13	.12	−1.14	.256
	**20% – 50%**	**0.35**	**.11**	**3.07**	**.004**

Table B3

Exploratory pairwise comparisons assessing impact of misinformation proportion condition on engagement behaviour across nudge conditions and true and false headlines

Condition	Misinformation proportion contrast	β	*SE*	*z*	*p*
True, No Nudge	**12.5% – 20%**	**−0.43**	**.15**	**−2.88**	**.012**
	12.5% – 50%	−0.21	.15	−1.41	.279
	20% – 50%	0.21	.15	1.48	.279
False, No Nudge	**12.5% – 20%**	**−0.48**	**.15**	**−3.16**	**.004**
	**12.5% – 50%**	**−0.49**	**.15**	**−3.23**	**.004**
	20% – 50%	<−0.01	.15	−0.04	.967
True, Nudge	**12.5% – 20%**	**−0.47**	**.16**	**−2.96**	**.006**
	12.5% – 50%	0.04	.16	0.23	.821
	**20% – 50%**	**0.51**	**.16**	**3.25**	**.004**
False, Nudge	**12.5% – 20%**	**−0.48**	**.17**	**−2.94**	**.010**
	12.5% – 50%	−0.30	.16	−1.83	.136
	20% – 50%	0.18	.16	1.13	.257

Table B4

Pairwise comparisons assessing impact of nudge condition across headline veracity and misinformation proportion conditions.

Headline Veracity	Misinformation Proportion	β	*SE*	*z*	*p*
False	**12.5%**	**0.34**	**.17**	**2.06**	**.039**
	**20%**	**0.33**	**.16**	**2.08**	**.037**
	50%	<0.01	.16	0.04	.969
True	12.5%	0.03	.15	0.17	.864
	20%	−0.03	.15	−0.18	.854
	50%	−0.25	.15	−1.68	.094

Table B5

ANODE Results for Engagement Behavior in 12.5% misinformation condition. Model: Engagement ~ Nudge Condition × Headline Veracity + (1 | Participant)

Fixed Effects	χ^2	*df*	*p*
Nudge	0.25	1	.618
Headline Veracity	**244.25**	**1**	**<.001**
Nudge × Headline Veracity	**16.49**	**1**	**<.001**

Table B6

ANODE Results for Engagement Behavior in 20% misinformation condition. Model: Engagement ~ Nudge Condition × Headline Veracity + (1 | Participant)

Fixed Effects	χ^2	*df*	*p*
Nudge	0.07	1	.794
Headline Veracity	**257.56**	**1**	**<.001**
Nudge × Headline Veracity	**25.32**	**1**	**<.001**

Table B7

ANODE Results for Engagement Behavior in 50% misinformation condition. Model: Engagement ~ Nudge Condition × Headline Veracity + (1 | Participant)

Fixed Effects	χ^2	*df*	*p*
Nudge	1.90	1	.168
Headline Veracity	**460.53**	**1**	**<.001**
Nudge × Headline Veracity	**10.50**	**1**	**.001**

ANODE results for full model ***without*** participant random intercepts

Results for analyses run on models without participant random intercepts are presented in Tables B8-B10[3]. The pattern of results between models including participant random intercepts and those which include Post, News source, Post order and Post source random intercepts (henceforth referred to as the *alternative model*) are generally comparable, however, the effect of the nudge intervention is somewhat stronger when analysed using the alternative model.

[3] We note that the inclusion of random slopes produced nonsensical estimates of correlation parameters (i.e., −1 and 1) in most analyses. Consequently, only random intercepts have been included in the statistical models [6].

Table B8

ANODE Results for Engagement Behavior
Model: Engagement ~ Misinformation Proportion × Nudge Condition × Headline Veracity + (1 | Post) + (1| News Source) + (1| Post Order) + (1| Source)

Fixed Effects	χ^2	*df*	*p*
Misinformation proportion	**218.69**	**2**	**< .001**
Nudge	0.25	1	.620
Headline Veracity	**7.66**	**1**	**.006**
Misinformation proportion × Nudge	**33.77**	**2**	**<.001**
Misinformation proportion × Headline Veracity	**19.16**	**2**	**<.001**
Nudge × Headline Veracity	**34.37**	**1**	**<.001**
Misinformation proportion × Nudge × Headline Veracity	0.90	2	.638

Table B9

Pairwise Comparisons Assessing Impact of Misinformation Proportion Condition on Engagement Behaviour Across Nudge Conditions and True and False Headlines

Condition	Misinformation proportion contrast	β	*SE*	*z*	*p*
True, No Nudge	12.5% – 20%	**−.28**	**.03**	**−9.56**	**<.001**
	12.5% – 50%	**−.16**	**.03**	**−5.33**	**<.001**
	20% – 50%	**.12**	**.03**	**4.32**	**<.001**
False, No Nudge	12.5% – 20%	**−.33**	**.06**	**−5.44**	**<.001**
	12.5% – 50%	.03	.06	0.53	.597
	20% – 50%	**.36**	**.06**	**6.08**	**<.001**
True, Nudge	12.5% – 20%	**−.25**	**.03**	**−8.53**	**<.001**
	12.5% – 50%	**−.31**	**.03**	**−10.76**	**<.001**
	20% – 50%	**−.06**	**.03**	**−1.98**	**.048**
False, Nudge	12.5% – 20%	**−.27**	**.07**	**−4.13**	**<.001**
	12.5% – 50%	**−.18**	**.07**	**−2.70**	**.014**
	20% – 50%	.09	.06	1.46	.146

Table B10

Pairwise Comparisons Assessing Impact of Misinformation Proportion Condition on Engagement Behaviour Across Nudge Conditions and True and False Headlines

Headline Veracity	Level of Misinformation	β	*SE*	*z*	*p*
False	12.5%	**.23**	**.07**	**3.56**	**<.001**
	20%	**.29**	**.06**	**4.93**	**<.001**
	50%	.02	.06	0.36	.717
True	12.5%	<.01	.03	−0.12	.903
	20%	.02	.03	0.75	.456
	50%	**−.16**	**.03**	**−5.66**	**<.001**

We note that the interaction between misinformation proportion and nudge is statistically significant in the alternative model (this interaction is non-significant in the model including only participant random intercepts). Due to this, we ran three additional 2 (misinformation proportion) × 2 (nudge condition) × 2 (headline veracity) analyses contrasting each misinformation proportion condition pairing (see Tables B11 – B13).

There was a significant effect of nudge across the 12.5% and 20% misinformation conditions, $\chi^2(1) = 9.04$, $p = .003$, qualified by a significant nudge × headline veracity interaction, $\chi^2(1) = 28.77$, $p < .001$, with higher engagement discernment in the nudge than the no nudge conditions. There was no significant nudge × misinformation proportion interaction, $\chi^2(1) = 0.72$, $p = .395$, with the impact of the nudge on engagement behavior relatively equivalent across the 12.5% and 20% misinformation conditions.

There was a fixed effect of nudge across the 12.5% and 50% misinformation conditions, $\chi^2(1) = 6.99$, $p = .008$, qualified by significant nudge × headline veracity, $\chi^2(1) = 17.54$, $p < .001$, and nudge × misinformation proportion interactions, $\chi^2(1) = 19.36$, $p < .001$. There was no fixed effect of nudge across the 20% and 50% misinformation conditions, $\chi^2(1) = 2.27$, $p = .132$, however, there were significant nudge × headline veracity, $\chi^2(1) = 23.28$, $p < .001$, and nudge × misinformation proportion interactions, $\chi^2(1) = 29.62$, $p < .001$. The significant nudge × headline veracity interactions again suggest engagement discernment was higher in the nudge conditions. Additionally, the nudge × misinformation proportion interactions suggest nudge impact varied depending on misinformation proportion. To deconstruct the differential effect of the nudge across misinformation proportion conditions, we isolated the effect of the nudge intervention on engagement discernment at each misinformation proportion (Tables B14 – B16). The nudge significantly improved engagement discernment in all conditions (12.5% misinformation condition; $\chi^2(1) = 10.99$, $p < .001$, 20% misinformation condition; $\chi^2(1) = 17.65$, $p < .001$, and 50% misinformation

conditions; $\chi^2(1) = 6.84$, $p = .009$). However, the pairwise comparisons run on the complete model (see Table C4) revealed the improved engagement discernment in the 12.5% and 20% misinformation conditions was driven by lower engagement with false headlines in the nudge than the no nudge condition, whereas the nudge was associated with no significant change in engagement with true headlines. In contrast, in the 50% misinformation conditions there was no significant difference in engagement with false headlines across the nudge and no nudge conditions. Rather, the improved engagement discernment in the nudge condition was driven by significantly higher engagement with true headlines in the nudge than the no nudge condition.

Table B11

ANODE Results for Engagement Behavior Across 12.5% and 20% Misinformation Conditions
Model: Engagement ~ Misinformation Proportion × Nudge Condition × Headline Veracity + (1 | Post) + (1| News Source) + (1| Post Order) + (1| Source)

Fixed Effects	χ^2	*df*	*p*
Misinformation proportion	**195.42**	**1**	**<.001**
Nudge	**9.04**	**1**	**.003**
Headline Veracity	**5.78**	**1**	**.016**
Misinformation proportion × Nudge	0.72	1	.395
Misinformation proportion × Headline Veracity	0.49	1	.486
Nudge × Headline Veracity	**28.77**	**1**	**<.001**
Misinformation proportion × Nudge × Headline Veracity	0.18	1	.673

Table B12

ANODE Results for Engagement Behavior Across 12.5% and 50% Misinformation Conditions
Model: Engagement ~ Misinformation proportion × Nudge Condition × Headline Veracity + (1 | Post) + (1| News Source) + (1| Post Order) + (1| Source)

Fixed Effects	χ^2	*df*	*p*
Misinformation proportion	**119.89**	**1**	**<.001**
Nudge	**6.99**	**1**	**.008**
Headline Veracity	**9.06**	**1**	**.003**
Misinformation proportion × Nudge	**19.36**	**1**	**<.001**
Misinformation proportion × Headline Veracity	**10.65**	**1**	**.001**
Nudge × Headline Veracity	**17.54**	**1**	**<.001**
Misinformation proportion × Nudge × Headline Veracity	0.32	1	.574

Table B13

ANODE Results for Engagement Behavior Across 20% and 50% Misinformation Conditions
Model: Engagement ~ Misinformation Proportion × Nudge Condition × Headline Veracity + (1 | Post) + (1| News Source) + (1| Post Order) + (1| Source)

Fixed Effects	χ^2	*df*	*p*
Misinformation proportion	**19.33**	**1**	**<.001**
Nudge	2.27	1	.132
Headline Veracity	**8.27**	**1**	**.004**
Misinformation proportion × Nudge	**29.62**	**1**	**<.001**
Misinformation proportion × Headline Veracity	**16.55**	**1**	**<.001**
Nudge × Headline Veracity	**23.28**	**1**	**<.001**
Misinformation proportion × Nudge × Headline Veracity	0.81	1	.368

Table B14

ANODE Results for Engagement Behavior in 12.5% Misinformation Condition
Model: Engagement ~ Nudge Condition × Headline Veracity + (1 | Post) + (1| News Source) + (1| Post Order) + (1| Source)

Fixed Effects	χ^2	*df*	*p*
Nudge	1.94	1	.164
Headline Veracity	**6.22**	**1**	**.013**
Nudge × Headline Veracity	**10.99**	**1**	**<.001**

Table B15

ANODE Results for Engagement Behavior in 20% Misinformation Condition
Model: Engagement ~ Nudge Condition × Headline Veracity + (1 | Post) + (1| News Source) + (1| Post Order) + (1| Source)

Fixed Effects	χ^2	*df*	*p*
Nudge	**7.92**	**1**	**.005**
Headline Veracity	**5.36**	**1**	**.021**
Nudge × Headline Veracity	**17.65**	**1**	**<.001**

Table B16

ANODE Results for Engagement Behavior in 50% Misinformation Condition
Model: Engagement ~ Nudge Condition × Headline Veracity + (1 | Post) + (1| News Source) + (1| Post Order) + (1| Source)

Fixed Effects	χ^2	*df*	*p*
Nudge	**24.00**	**1**	**<.001**
Headline Veracity	**11.80**	**1**	**<.001**
Nudge × Headline Veracity	**6.84**	**1**	**.009**

References

[1] Christensen, R. H. B. Cumulative link models for ordinal regression with the R Package ordinal. *Unsubmitted Manuscript* 1–40 (2018)

[2] Wickham, H. Data Analysis. 189–201 (2016) doi:10.1007/978-3-319-24277-4_9

[3] Langsrud, Ø. ANOVA for unbalanced data: Use Type II instead of Type III sums of squares. *Stat Comput* **13**, (2003)

[4] Herve, M. RVAideMemoire: Testing and plotting procedures for biostatistics. Preprint at (2022)

[5] Lenth, R. V. *et al.* emmeans: Estimated Marginal Means, aka Least-Squares Means. Preprint at (2023)

[6] Matuschek, H., Kliegl, R., Vasishth, S., Baayen, H. & Bates, D. Balancing Type I error and power in linear mixed models. *J Mem Lang* **94**, 305–315 (2017)
