# Supplementary material for: Nudge-based misinformation interventions are effective in information environments with low misinformation prevalence
Source: Sci Rep. 2024 May 20;14:11495. doi: 10.1038/s41598-024-62286-7 (PMC11106285; doi:10.1038/s41598-024-62286-7)
Supplement: Supplementary file 3 — Supplementary Information 3. [file 41598_2024_62286_MOESM3_ESM.pdf]

Supplementary materials for *"Nudge-Based Misinformation Interventions are Effective in Information Environments with Low Misinformation Prevalence"*

Supplement C – Results Output, Supplementary Analyses

Table of Contents

Attrition Check 2

Assumption check 3

Isolating analyses to first 50 posts 4

Belief in true and false headlines across nudge and misinformation proportion 8

Political Orientation 11

The effect of post order on engagement behavior 17

Interaction with social posts in the 12.5% misinformation condition 22

References 25

Attrition Check

Given the relatively high proportion of participants who either (1) started but did not complete the study, or (2) failed at least one of the pre-registered exclusion criteria, we first assess whether there were significant differences in level of attrition, both pre- and post-exclusion criteria being applied (see Table C1 for level of attrition across conditions). Across conditions and their interaction, there were no significant differences in level of attrition pre-exclusion criteria being applied (Table C2), however, there was a significant main effect of misinformation proportion condition on attrition following the application of the exclusion criteria (though follow-up contrasts revealed no significant differences in level of attrition across the pairs of misinformation proportion conditions, see Table C4). Furthermore, there was no significant difference in number of participants excluded across conditions or their interaction (Table C5).

Table C1

Level of Attrition across Nudge and Misinformation Proportion Conditions, both Pre- and Post-Exclusion Criteria being applied

Nudge Condition	Misinformation Proportion	Pre-Exclusion Attrition	Post-Exclusion Attrition
No Nudge	12.5%	40	63
	20%	24	34
	50%	29	46
Nudge	12.5%	39	61
	20%	44	61
	50%	33	52

Table C2

Attrition across conditions and their interaction, pre-exclusion criteria being applied, Type 3 ANODE

Fixed Effects	χ^2	*df*	*p*
Misinformation Proportion	2.11	2	.347
Nudge	2.54	1	.111
Misinformation Proportion × Nudge	3.70	2	.157

Table C3

Attrition across conditions and their interaction, post-exclusion criteria being applied, Type 3 ANODE

Fixed Effects	χ^2	*df*	*p*
**Misinformation Proportion**	**8.89**	**2**	**.012**
Nudge	0.3	1	.857
Misinformation Proportion × Nudge	5.14	2	.076

Table C4

Follow-up contrast (Holm-Bonferroni corrected) contrasting attrition across misinformation proportion conditions

Contrast	*OR*	*SE*	*z*	*p*
12.5% - 20%	1.36	.19	2.21	.082
12.5% - 50%	1.27	.17	1.75	.160
20% - 50%	0.93	.14	−0.48	.628

Table C5

Assessing if there is a significant difference in level of exclusions across conditions and their interaction, Type 3 ANODE

Fixed Effects	χ^2	*df*	*p*
Misinformation Proportion	5.27	2	.072
Nudge	0.02	1	.881
Misinformation Proportion × Nudge	1.38	2	.503

Assumption check

For participants in the 50% misinformation conditions, we initially checked to ensure engagement (overall engagement, sharing, liking) with the 10 target false headlines was not significantly different from engagement with the 30 filler headlines. There was no significant difference in either sharing or liking of target or filler posts (see Tables C6 – C7). Accordingly, for all subsequent analyses all 40 false headlines were retained in the 50% misinformation condition.

Table C6

ANODE (Type 3) Results for Sharing Behavior in 50% Misinformation Condition, Comparing Target and Filler False Posts
Model: Share ~ Nudge Condition × Headline Type (target vs filler) + (1 + Nudge Condition| Post) + (1 + Headline Type| Participant)

Fixed Effects	χ^2	*df*	*p*

Nudge	0.02	1	.901
Headline Type	0.82	1	.364
Nudge × Headline Veracity	0.24	1	.621

Table C7

ANODE (Type 3) Results for Liking Behavior in 50% Misinformation Condition, Comparing Target and Filler False Posts
Model: Like ~ Nudge Condition × Headline Type (target vs filler) + (1 + Nudge Condition| Post) + (1 + Headline Type| Participant)

Fixed Effects	χ^2	*df*	*p*
Nudge	0.10	1	.754
Headline Type	0.17	1	.684
Nudge × Headline Veracity	0.44	1	.510

Isolating analyses to first 50 posts

To assess whether the higher average level of engagement with true and false posts in the 20% misinformation condition was driven by the reduced number of headlines presented in the condition (50 compared to 80) we reran the main analysis isolated to the first 50 posts participants were presented (see Tables C8 and C9 for results for sharing behavior, and Tables C10 and C11 for results for liking behavior). This analysis was not pre-registered and thus is entirely exploratory. The pattern of results for sharing behavior remains consistent with the full dataset. As such, it appears that the different level of sharing of true and false headlines across misinformation proportion conditions was not primarily driven by the reduced number of posts in the 20% misinformation condition. However, although the results for liking behavior were largely consistent with the main analyses, liking of headlines was not significantly different across the 20% and 50% conditions (collapsed across headline types and nudge conditions). This suggests the difference observed in these conditions in the main analyses may have been inflated by the reduced number of headlines in the 20% condition (and participants liking posts more at the start than the end of the simulation). Average sharing and liking of true and false posts across nudge and misinformation proportion conditions are presented in Figures C1 and C2.

Figure C1

Sharing Frequency for False and True Headlines Across Nudge and Misinformation Proportion Conditions isolated to the first 50 posts participants saw.

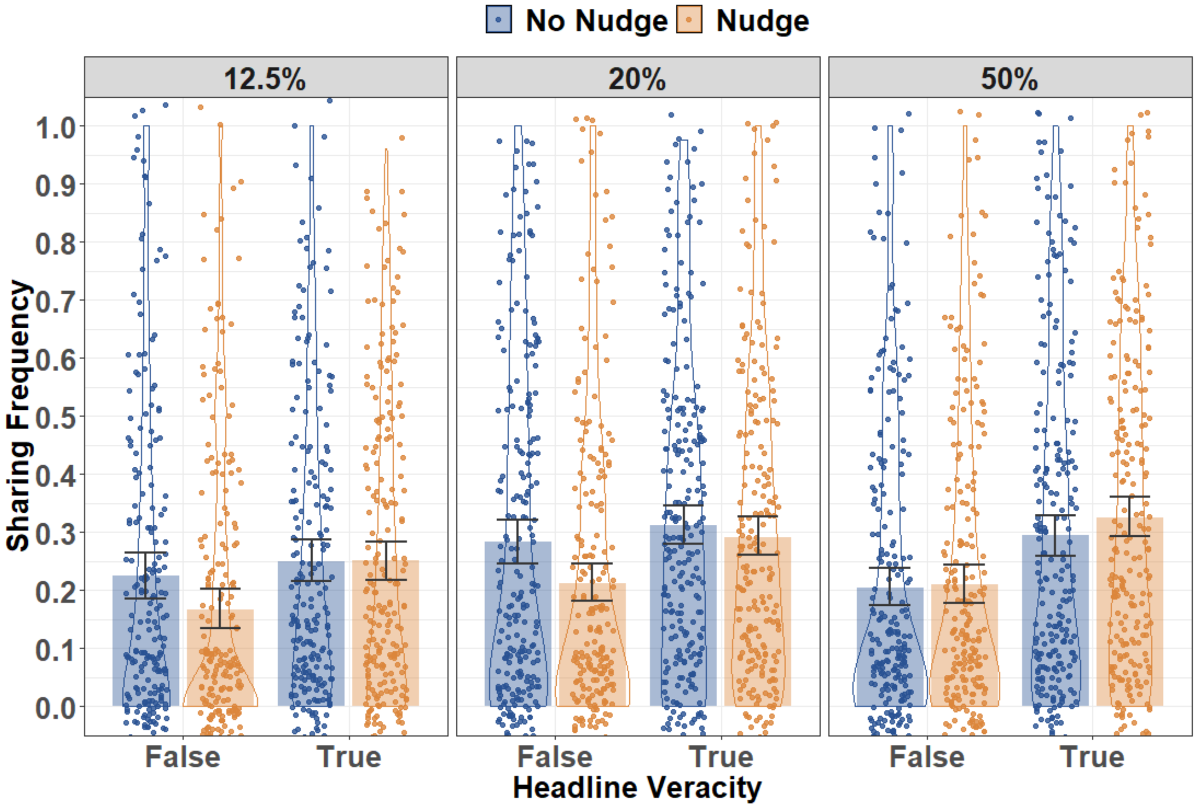

Note. 12.5%, 20%, and 50% Refer to the Corresponding Misinformation-Proportion Conditions. Bars Show Condition Means; Error Bars Represent 95% Confidence Intervals; Jittered Dots Represent Individual Participant Means; Violins Provide Distributional Information

Table C8

ANODE (Type 3) Results for Sharing Behavior, Isolated to the First 50 Posts Participants Saw
Model: Share ~ Misinformation Proportion × Nudge Condition × Headline Veracity + (1 + Headline Veracity | Participant) + (1 +Misinformation Proprtion × Nudge Condition| Post), family = binomial , glmerControl(optimizer = "bobyqa")

Fixed Effects	χ^2	*df*	*p*
Misinformation proportion	**12.13**	**2**	**.002**
Nudge	1.56	1	.212
Headline Veracity	**18.48**	**1**	**<.001**
Misinformation proportion × Nudge	4.15	2	.125
Misinformation proportion × Headline Veracity	**10.13**	**2**	**.006**
Nudge × Headline Veracity	**9.24**	**1**	**.002**
Misinformation proportion × Nudge × Headline Veracity	4.27	2	.118

Table C9

Pairwise Comparisons Assessing Impact of Misinformation Proportion Condition on Sharing Behavior Across True and False Headlines, collapsed across Nudge Conditions Isolated to the First 50 Headlines participants were Presented

Condition	Levels of misinformation contrast	*Odds ratio*	*SE*	*z*	*p*
True	**12.5% – 20%**	**0.62**	**.09**	**−3.22**	**.003**
	**12.5% – 50%**	**0.60**	**.09**	**−3.49**	**.002**
	20% – 50%	0.96	.13	−0.30	.763
False	**12.5% – 20%**	**0.57**	**.11**	**−3.02**	**.008**
	12.5% – 50%	0.78	.15	−1.27	.203
	20% – 50%	1.38	.25	1.80	.144

Figure C2

Liking Frequency for False and True Headlines Across Nudge and Misinformation Proportion Conditions isolated to the first 50 posts participants saw.

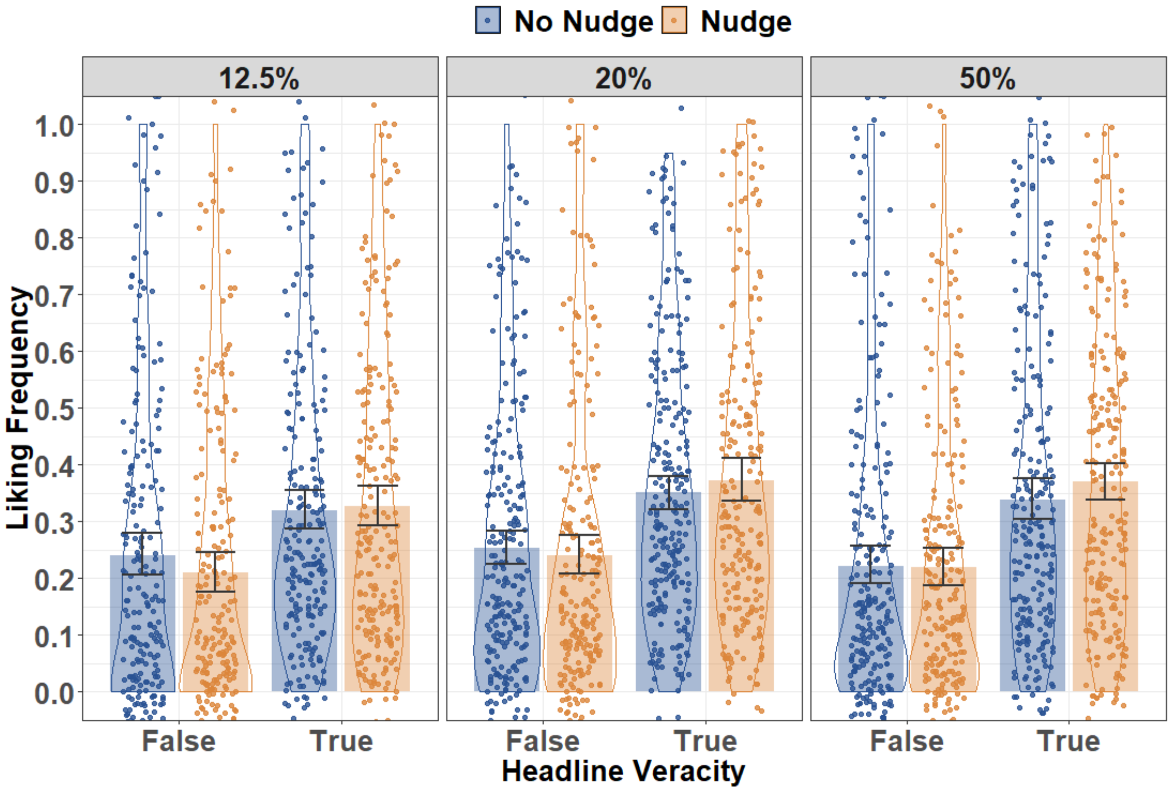

Note. 12.5%, 20%, and 50% Refer to the Corresponding Misinformation-Proportion Conditions. Bars Show Condition Means; Error Bars Represent 95% Confidence Intervals; Jittered Dots Represent Individual Participant Means; Violins Provide Distributional Information

Table C10

ANODE (Type 3) Results for Liking Behavior, Isolated to the First 50 Posts Participants Saw
Model: Like ~ Misinformation Proportion × Nudge Condition × Headline Veracity + (1 + Headline Veracity | Participant) + (1 +Misinformation Proprtion × Nudge Condition| Post), family = binomial , glmerControl(optimizer = "bobyqa")

Fixed Effects	χ^2	*df*	*p*
Misinformation proportion	**6.49**	**2**	**.039**
Nudge	0.35	1	.553
Headline Veracity	**18.49**	**1**	**<.001**
Misinformation proportion × Nudge	0.87	2	.649
Misinformation proportion × Headline Veracity	3.87	2	.144
Nudge × Headline Veracity	2.83	1	.093
Misinformation proportion × Nudge × Headline Veracity	0.14	2	.932

Table C11

Pairwise Comparisons Assessing Impact of Misinformation Proportion Condition on Liking Behavior, Collapsed Across Nudge Conditions and True and False Headlines Isolated to the First 50 Headlines participants were Presented

Levels of misinformation contrast	*Odds ratio*	*SE*	*z*	*p*
**12.5% – 20%**	**0.74**	**.09**	**−2.49**	**.038**
12.5% – 50%	0.87	.11	−1.12	.342
20% – 50%	1.18	.14	1.37	.342

Belief in true and false headlines across nudge and misinformation proportion

Belief in true and false headlines was assessed at the end of the study. As nudge interventions are not specifically intended to impact belief, and because it is possible that how participants engaged with headlines in the engagement task may have impacted their reported belief in the headline, belief in misinformation was primarily included for exploratory purposes. Average belief split by condition and headline type is presented in Figure C3, and ANODE results (based on generalised linear mixed effects model) are presented in Table C12.

Figure C3

Belief in True and False Headlines Across Nudge and Misinformation Proportion Conditions

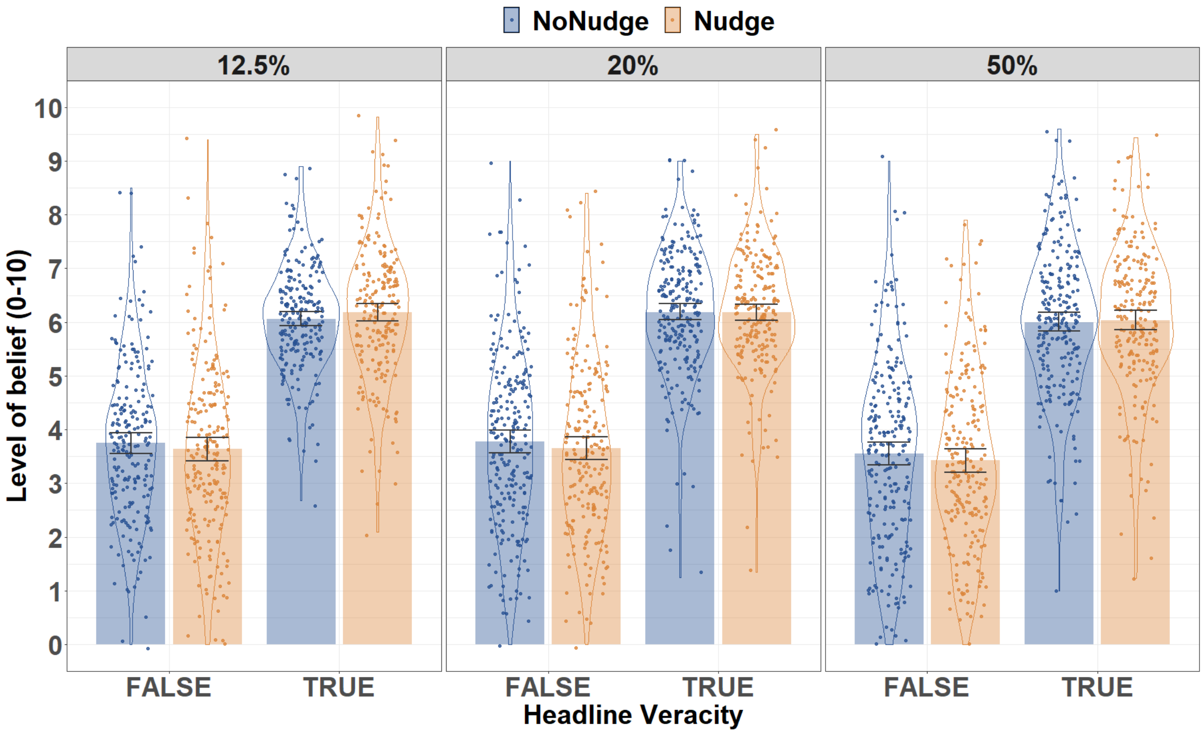

Note. Error bars represent 95% confidence intervals.

Table C12

ANODE (Type 3) Results for Belief in True and False Headlines, Split by Nudge and Misinformation Proportion Conditions
Model: Belief ~ Misinformation Proportion × Nudge Condition × Headline Veracity + (1 + Misinformation Proportion × Nudge |Post) + (1 + Headline Veracity | Participant)

Fixed Effects	χ^2	*df*	*p*
Misinformation proportion	**6.52**	**2**	**.038**
Nudge	0.01	1	.913
Headline Veracity	**212.36**	**1**	**<.001**
Misinformation proportion × Nudge	0.19	2	.909
Misinformation proportion × Headline Veracity	1.33	2	.515
Nudge × Headline Veracity	2.78	1	.095
Misinformation proportion × Nudge × Headline Veracity	0.52	2	.770

There was a significant main effect of headline veracity, with belief in true headlines significantly higher than belief in false headlines. There was additionally a marginally significant effect of misinformation proportion condition, however, follow-up contrasts revealed no significant difference in belief across any of the misinformation proportion conditions, see Table C13.

Table C13

Pairwise Comparisons Assessing Impact of Misinformation Proportion Condition on Belief, Collapsed Across Nudge Conditions and True and False Headlines

Levels of misinformation contrast	β	*SE*	*z*	*p*
12.5% – 20%	−.03	.08	−0.32	.748
12.5% – 50%	.17	.08	2.09	.074
20% – 50%	.20	.08	2.37	.053

For completeness, we correlated belief with the impact of the nudge on sharing and liking behavior (i.e., a difference score created by calculating $M_{\text{Nudge}} - M_{\text{No Nudge}}$ for each post) across the three misinformation proportion conditions (see Figures C4 and C5). Note that for difference in engagement (sharing or liking), any value below 0 indicates that average engagement with the headline was lower in the nudge than in the no nudge condition, and any value above 0 indicates average engagement with the headline was higher in the nudge than

the no nudge condition. As with prior research (e.g., [1,2]), belief was significantly correlated with nudge impact across all three misinformation proportion conditions (see Table C14).

Table C14

Correlations Between Belief in Headlines and Difference in Sharing and Liking Between Nudge and No-Nudge Conditions Across 12.5%, 20%, and 50% Misinformation Conditions

Engagement Type	Levels of misinformation	*r*	*p*
Sharing	12.5%	**.55**	**<.001**
	20%	**.52**	**<.001**
	50%	**.58**	**<.001**
Liking	12.5%	**.52**	**<.001**
	20%	**.45**	**<.001**
	50%	**.46**	**<.001**

Figure C4

Scatterplot (With Best-Fitting Regression Line) Depicting Association Between Average Belief (Averaged Across Nudge Conditions) and Average Difference in Sharing ($M_{\text{nudge}} - M_{\text{no Nudge}}$) With Each False Headline (Blue) and True Headline (Orange) in the Nudge Compared to the No Nudge Condition

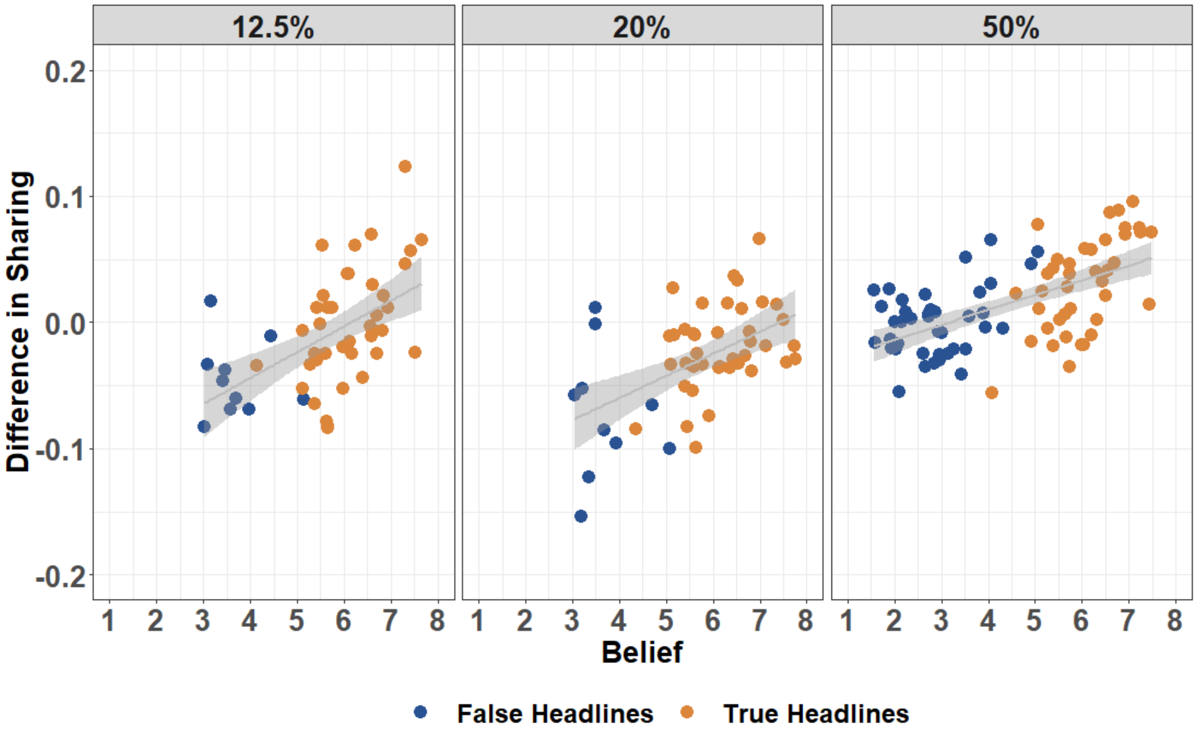

Figure C5

Scatterplot (With Best-Fitting Regression Line) Depicting Association Between Average Belief (Averaged Across Nudge Conditions) and Average Difference in Liking ($M_{nudge} - M_{no\ Nudge}$) With Each False Headline (Blue) and True Headline (Orange) in the Nudge Compared to the No Nudge Condition

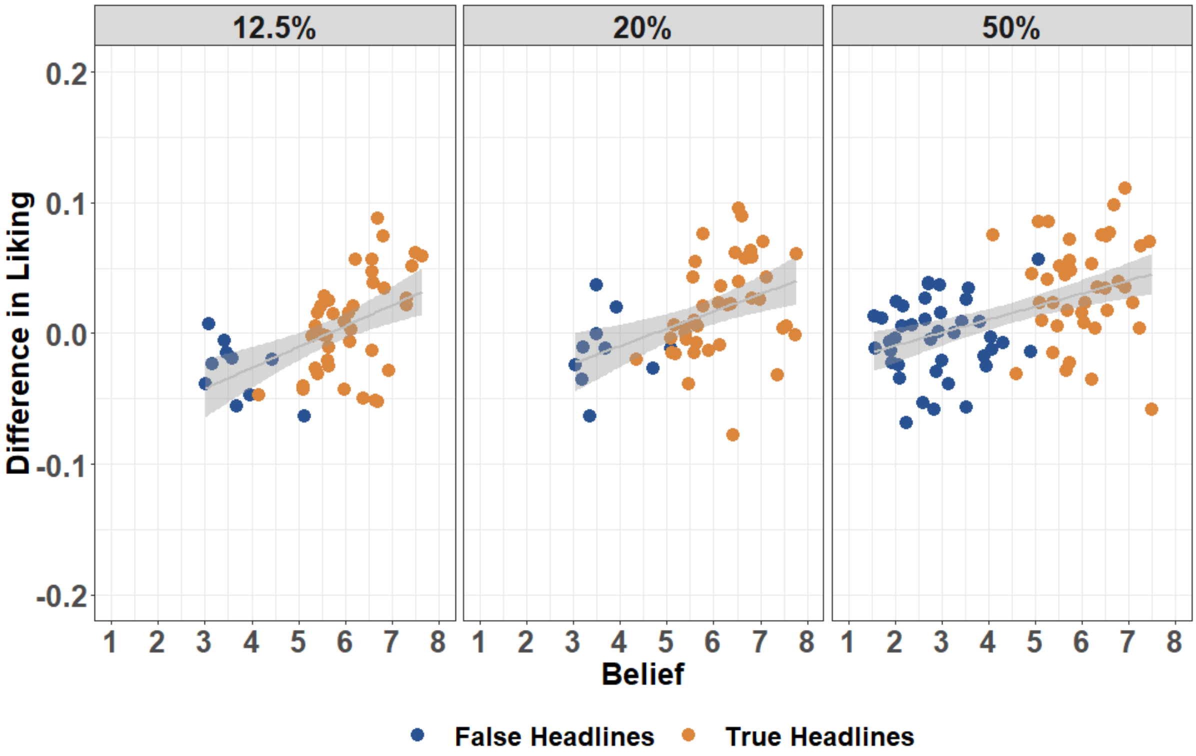

Political Orientation

We then assessed whether the effect of the nudge intervention on sharing and liking behavior significantly differed over the political spectrum (this analysis was initially pre-registered to focus on the impact of political orientation on engagement behavior, and thus we again slightly deviate from the preregistration here). To do so, political orientation (1-7) was treated as a continuous predictor in the model, and analyses were run separately for each misinformation proportion condition given the complexity of the model (see Tables C15 – C17 for results on sharing behavior, and Tables C18 – C20 for results on liking behavior). Results split by political orientation (liberal, conservative) are displayed in Figure C6 for sharing behavior, and Figure C7 for liking behavior. Across all three misinformation

proportion conditions there were no significant political orientation × nudge interactions, or three-way political orientation × nudge × headline veracity interactions across either sharing or liking behavior[1]. We do note that there were significant political orientation × headline veracity interactions across all misinformation proportion conditions and liking and sharing behavior, with greater conservatism associated with more engagement with both false and true headlines, particularly false headlines.

Table C15

ANODE (Type 3) Results for Sharing Behavior including political orientation (1-7) as a continuous factor, isolated to the 12.5% misinformation condition. Model: Sharing ~ Political Orientation × Nudge Condition × Headline Veracity + (1 + Headline Veracity | Participant) + (1 + Nudge × Political Orientation | Post)

Fixed Effects	χ^2	*df*	*p*
Political orientation	0.90	1	.342
Nudge	0.97	1	.325
Headline veracity	**10.23**	**1**	**.001**
Political orientation × Nudge	2.52	1	.112
Political orientation × Headline veracity	**12.09**	**1**	**<.001**
Nudge × Headline veracity	**6.47**	**1**	**.011**
Political orientation × Nudge × Headline veracity	0.64	1	.423

Table C16

ANODE (Type 3) Results for Sharing Behavior including political orientation (1-7) as a continuous factor, isolated to the 20% misinformation condition. Model: Sharing ~ Political Orientation × Nudge Condition × Headline Veracity + (1 + Headline Veracity | Participant) + (1 + Nudge × Political Orientation | Post)

Fixed Effects	χ^2	*df*	*p*
Political orientation	**6.93**	**1**	**.008**
Nudge	1.47	1	.225
Headline veracity	**8.23**	**1**	**.004**
Political orientation × Nudge	0.02	1	.881
Political orientation × Headline veracity	**22.15**	**1**	**<.001**
Nudge × Headline veracity	**10.59**	**1**	**.001**
Political orientation × Nudge × Headline veracity	1.23	1	.267

[1] We do note, however, that the current sample was unbalanced on political orientation. As such, our ability to draw conclusions on the effect of the nudge intervention across the political spectrum is limited.

Table C17

ANODE (Type 3) Results for Sharing Behavior including political orientation (1-7) as a continuous factor, isolated to the 50% misinformation condition. Model: Sharing ~ Political Orientation × Nudge Condition × Headline Veracity + (1 + Headline Veracity | Participant) + (1 + Nudge × Political Orientation | Post)

Fixed Effects	χ^2	*df*	*p*
Political orientation	0.73	1	.394
Nudge	0.51	1	.476
Headline veracity	**63.75**	**1**	**<.001**
Political orientation × Nudge	0.25	1	.619
Political orientation × Headline veracity	**50.48**	**1**	**<.001**
Nudge × Headline veracity	**8.14**	**1**	**.004**
Political orientation × Nudge × Headline veracity	0.24	1	.623

Table C18

ANODE (Type 3) Results for Liking Behavior including political orientation (1-7) as a continuous factor, isolated to the 12.5% misinformation condition. Model: Liking ~ Political Orientation × Nudge Condition × Headline Veracity + (1 + Headline Veracity | Participant) + (1 + Nudge × Political Orientation | Post)

Fixed Effects	χ^2	*df*	*p*
Political orientation	2.47	1	.116
Nudge	0.18	1	.675
Headline veracity	**9.25**	**1**	**.002**
Political orientation × Nudge	0.02	1	.891
Political orientation × Headline veracity	**15.05**	**1**	**<.001**
Nudge × Headline veracity	**5.80**	**1**	**.016**
Political orientation × Nudge × Headline veracity	1.43	1	.231

Table C19

ANODE (Type 3) Results for Liking Behavior including political orientation (1-7) as a continuous factor, isolated to the 20% misinformation condition. Model: Liking ~ Political Orientation × Nudge Condition × Headline Veracity + (1 + Headline Veracity | Participant) + (1 + Nudge × Political Orientation | Post)

Fixed Effects	χ^2	*df*	*p*
Political orientation	**4.26**	**1**	**.039**
Nudge	0.06	1	.801
Headline veracity	**7.81**	**1**	**.005**
Political orientation × Nudge	2.88	1	.090
Political orientation × Headline veracity	**22.03**	**1**	**<.001**
Nudge × Headline veracity	**7.30**	**1**	**.007**
Political orientation × Nudge × Headline veracity	0.50	1	.480

Table C20

ANODE (Type 3) Results for Liking Behavior including political orientation (1-7) as a continuous factor, isolated to the 50% misinformation condition. Model: Liking ~ Political Orientation × Nudge Condition × Headline Veracity + (1 + Headline Veracity | Participant) + (1 + Nudge × Political Orientation | Post)

Fixed Effects	χ^2	*df*	*p*
Political orientation	**9.58**	**1**	**.002**
Nudge	0.10	1	.748
Headline veracity	**42.49**	**1**	**<.001**
Political orientation × Nudge	1.06	1	.304
Political orientation × Headline veracity	**43.66**	**1**	**<.001**
Nudge × Headline veracity	**7.62**	**1**	**.006**
Political orientation × Nudge × Headline veracity	0.05	1	.828

Figure C6

Sharing of true and false headlines split by misinformation proportion and nudge conditions for liberal (left) and conservative (right) participants.

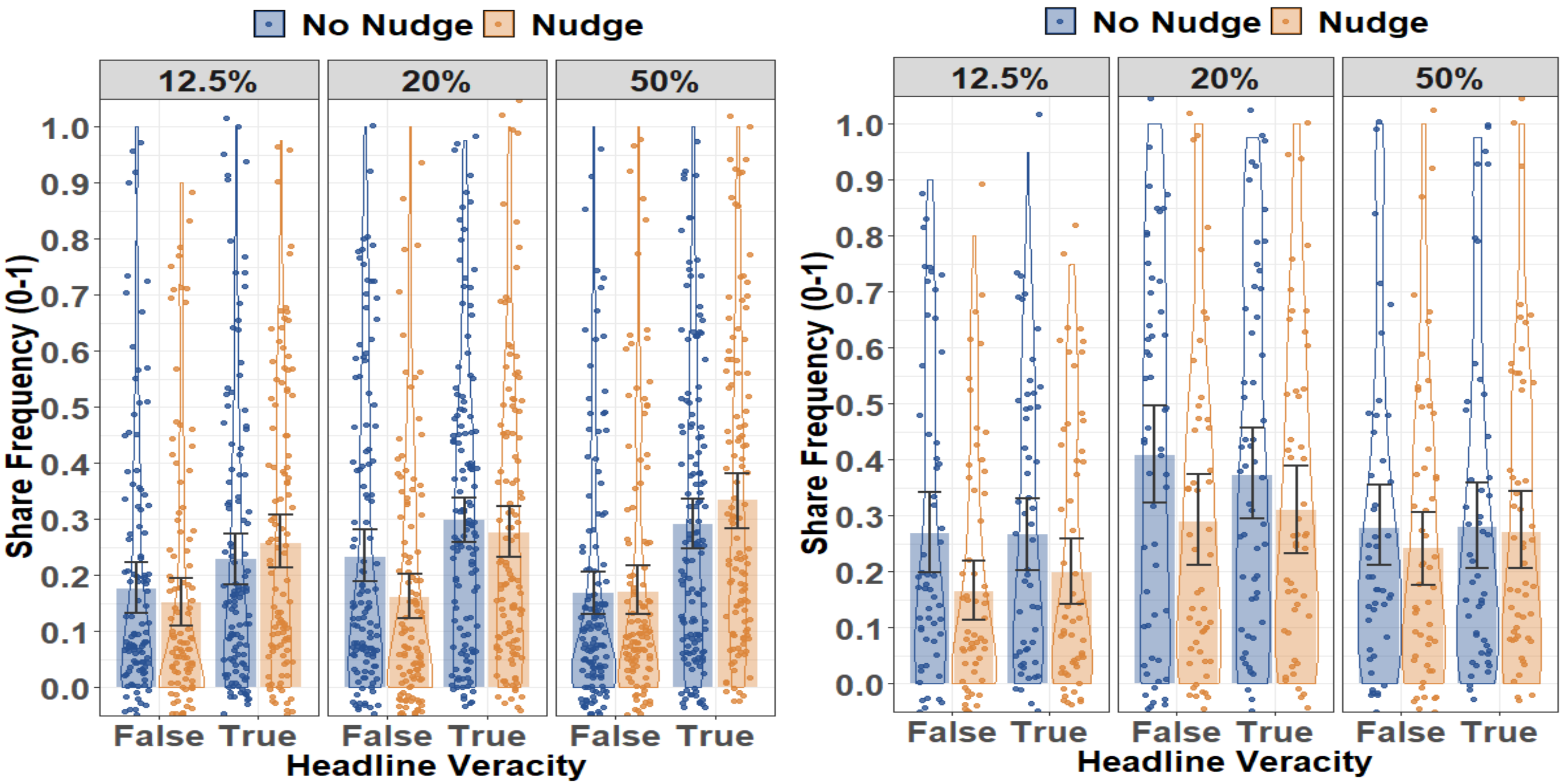

Note. Participants who responded 1 (strongly liberal) – 3 (leaning liberal) on the political orientation question were classed as liberal, and those who responded 5 (leaning conservative) – 7 (strongly conservative) were classed as conservative for graphical purposes. Participants who reported to be centralists are not presented in the graphs. Error bars represent 95% confidence intervals.

Figure C7

Liking of true and false headlines split by misinformation proportion and nudge conditions for liberal (left) and conservative (right) participants.

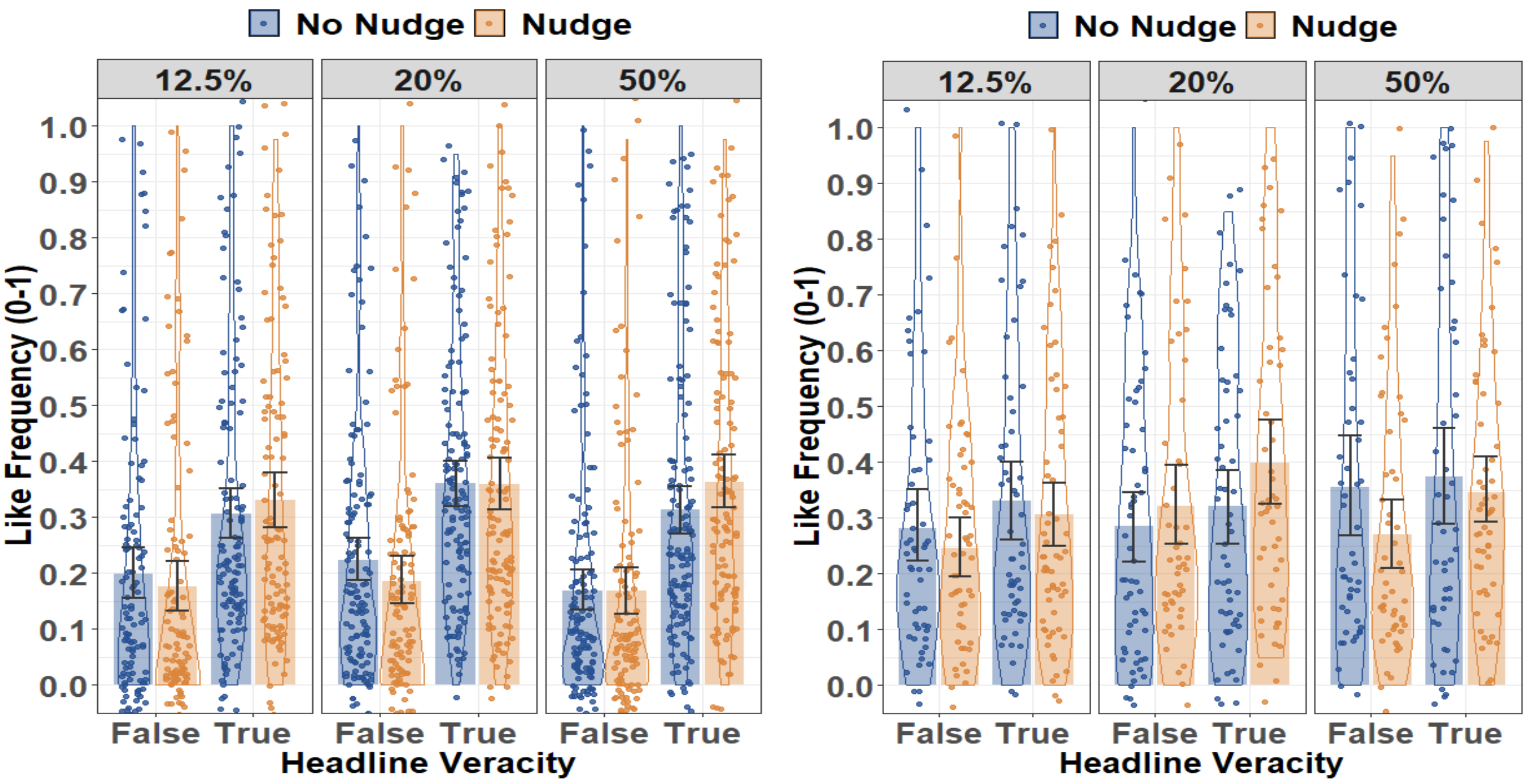

Note. Participants who responded 1 (strongly liberal) – 3 (leaning liberal) on the political orientation question were classed as liberal, and those who responded 5 (leaning conservative) – 7 (strongly conservative) were classed as conservative for graphical purposes. Participants who reported to be centralists are not presented in the graphs. Error bars represent 95% confidence intervals.

The effect of post order on engagement behavior

Due to prior research suggesting nudge-based interventions may only be effective on the first few posts a participant is exposed to (see Roozenbeek et al., 2021), we ran analyses assessing if the effect of the nudge decreased over the course of the study. Specifically, post display order (1-50, or 1-80 dependent on condition) was included in the statistical models as a continuous predictor, and analyses were run separately for each misinformation proportion condition given the complexity of the models. These analyses were not pre-registered and thus are entirely exploratory and should be interpreted as such. The impact of post order on sharing behavior is displayed in Tables C21 – C23, and liking behavior is displayed in Tables C24 – C26, descriptives are presented in Figure C8). Except for sharing behavior in the 20% misinformation conditions, there was a significant effect of post order, with participants liking and sharing more at the beginning of the task than the end. Note that the lack of a significant effect of post order on sharing behavior in the 20% misinformation condition was likely, at least in part, due to the reduced number of headlines presented in this condition. Furthermore, in the 20% misinformation condition there was a post order × headline veracity interaction for liking behavior, with participants significantly reducing level of liking of true, but not false, headlines over the course of the study.

By contrast, there were no significant post order × nudge, or 3-way post order × nudge × headline veracity interactions, suggesting the effect of the nudge intervention did not significantly change over the course of the study, though we are likely underpowered to detect such an effect (particularly a 3-way interaction). In fact, visual inspection of the graphs for the 12.5% suggests that the effect of the nudge on engagement was numerically more pronounced at the start of the task than at the end (moreover, at the end of the study there was minimal numeric difference in engagement with false headlines between the nudge and no nudge conditions). As such, numerically, the results suggest that the nudge intervention did

decay relatively quickly post exposure within this condition (however, the pattern of results was somewhat reversed in the 50% misinformation condition). However, given the results are non-significant, we do not make any specific claims about whether this decay would generalize across conditions or stimuli, or whether this pattern reflects a genuine reduction in the effectiveness of the nudge intervention or occurred by chance.

Table C21

ANODE (Type 3) Results for Sharing Behavior over the Course of the Experiment, Isolated to the 12.5% Misinformation Condition
Model: Share ~ Post Order × Nudge Condition × Headline Veracity + (1 + Headline Veracity | Participant) + (1 + Post Order × Nudge | Post)

Fixed Effects	χ^2	*df*	*p*
Post order	**49.91**	**1**	**<.001**
Nudge	1.60	1	.206
Headline veracity	**9.58**	**1**	**.002**
Post order × Nudge	1.42	1	.233
Post order × Headline veracity	0.13	1	.721
Nudge × Headline veracity	**6.69**	**1**	**.010**
Post order × Nudge × Headline veracity	0.25	1	.618

Table C22

ANODE Results for Sharing Behavior over the Course of the Experiment, Isolated to the 20% Misinformation Condition
Model: Share ~ Post Order × Nudge Condition × Headline Veracity + (1 + Headline Veracity | Participant) + (1 + Post Order × Nudge | Post)

Fixed Effects	χ^2	*df*	*p*
Post order	**4.11**	**1**	**.043**
Nudge	1.44	1	.230
Headline veracity	**10.42**	**1**	**.001**
Post order × Nudge	0.28	1	.599
Post order × Headline veracity	1.43	1	.232
Nudge × Headline veracity	**8.30**	**1**	**.004**
Post order × Nudge × Headline veracity	<.01	1	.932

Table C23

ANODE Results for Sharing Behavior over the Course of the Experiment, Isolated to the 50% Misinformation Condition
Model: Share ~ Post Order × Nudge Condition × Headline Veracity + (1 + Headline Veracity | Participant) + (1 + Post Order × Nudge | Post)

Fixed Effects	χ^2	*df*	*p*
Post order	**48.70**	**1**	**<.001**
Nudge	0.89	1	.347
Headline veracity	**63.89**	**1**	**<.001**
Post order × Nudge	0.56	1	.453
Post order × Headline veracity	0.04	1	.845
Nudge × Headline veracity	3.20	1	.074
Post order × Nudge × Headline veracity	2.20	1	.138

Table C24

ANODE (Type 3) Results for Liking Behavior over the Course of the Experiment, Isolated to the 12.5% Misinformation Condition
Model: Like ~ Post Order × Nudge Condition × Headline Veracity + (1 + Headline Veracity | Participant) + (1 + Post Order × Nudge | Post)

Fixed Effects	χ^2	*df*	*p*
Post order	**46.34**	**1**	**<.001**
Nudge	0.33	1	.568
Headline veracity	**7.83**	**1**	**.005**
Post order × Nudge	0.15	1	.695
Post order × Headline veracity	0.21	1	.646
Nudge × Headline veracity	2.91	1	.088
Post order × Nudge × Headline veracity	0.10	1	.751

Table C25

ANODE (Type 3) Results for Liking Behavior over the Course of the Experiment, Isolated to the 20% Misinformation Condition
Model: Like ~ Post Order × Nudge Condition × Headline Veracity + (1 + Headline Veracity | Participant) + (1 + Post Order × Nudge | Post)

Fixed Effects	χ^2	*df*	*p*
Post order	**36.55**	**1**	**<.001**
Nudge	<.01	1	.990
Headline veracity	**6.80**	**1**	**.009**
Post order × Nudge	0.44	1	.506
Post order × Headline veracity	**3.91**	**1**	**048**
Nudge × Headline veracity	2.93	1	.087
Post order × Nudge × Headline veracity	0.68	1	.408

Table C26

ANODE (Type 3) Results for Liking Behavior over the Course of the Experiment, Isolated to the 50% Misinformation Condition
Model: Like ~ Post Order × Nudge Condition × Headline Veracity + (1 + Headline Veracity | Participant) + (1 + Post Order × Nudge | Post)

Fixed Effects	χ^2	*df*	*p*
Post order	**136.48**	**1**	**<.001**
Nudge	0.52	1	.470
Headline veracity	**39.75**	**1**	**<.001**
Post order × Nudge	0.39	1	.533
Post order × Headline veracity	2.46	1	.117
Nudge × Headline veracity	3.43	1	.064
Post order × Nudge × Headline veracity	0.53	1	.467

Figure C8

Frequency of Sharing (a) and Liking (b) of False (Left Panel) and True (Right Panel) Headlines over the Course of the Experiment. Note, 12.5%, 20%, and 50% refer to the corresponding misinformation-proportion conditions. Shaded areas represent standard error. Jitters represents mean engagement in the no nudge (blue) and nudge (orange) conditions at each post position (as a proxy for timepoint).

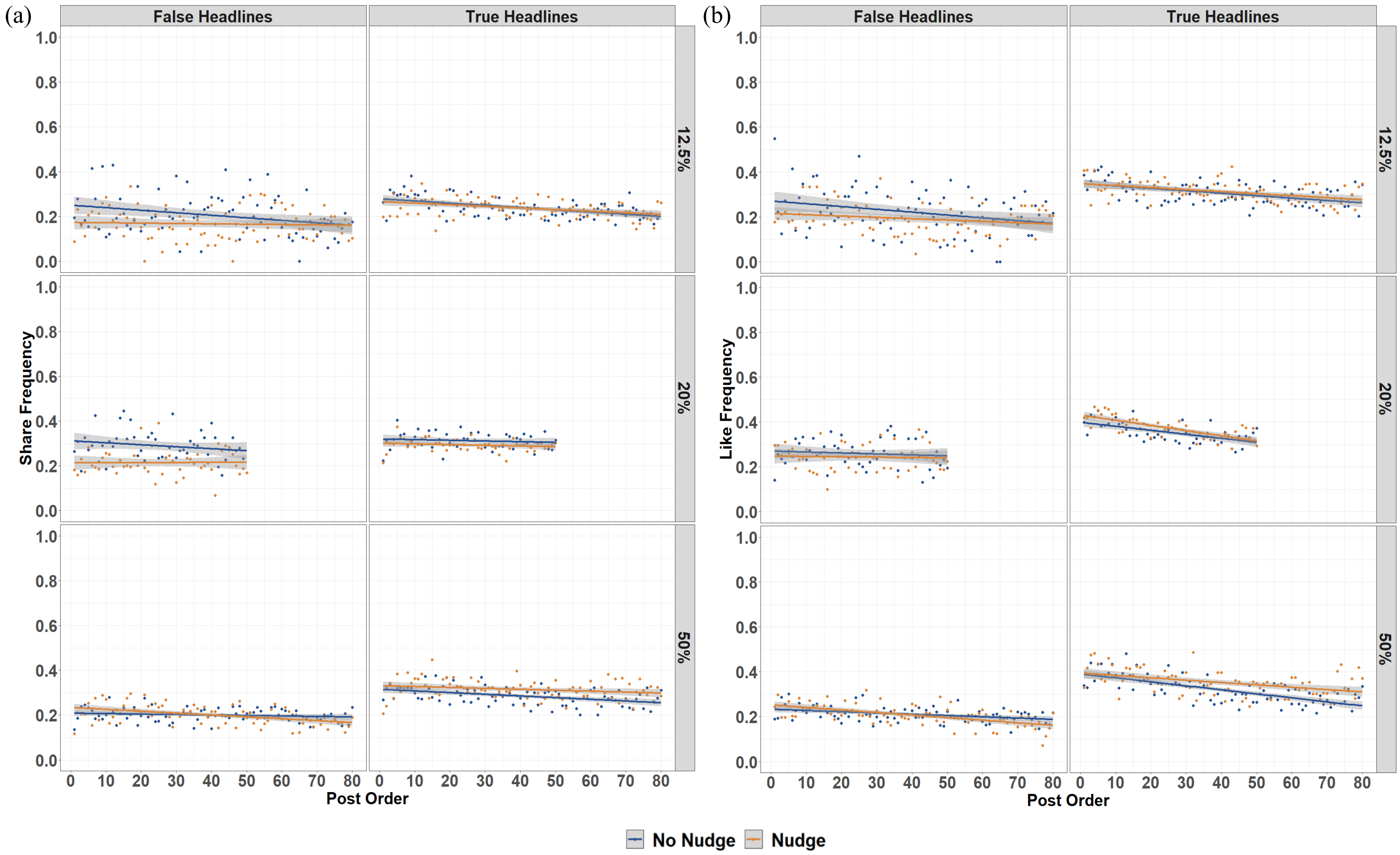

Interaction with social posts in the 12.5% misinformation condition

To assess how participants interacted with social compared to true and false posts, we ran additional analyses comparing engagement (sharing and liking) with posts across all three post types in the 12.5% misinformation condition (i.e., the only condition including social posts). Sharing of each post type across nudge conditions is shown in Figure C9, and liking of each post type across nudge conditions is displayed in Figure C10. For sharing behavior, there was a significant main effect of post type, qualified by a significant post type nudge condition interaction (see Table C27). Follow-up contrasts reveal that participants shared social posts significantly more than true headlines across both nudge and no-nudge conditions. There was no difference in sharing of false and social posts across either nudge condition; see Table C28.

There was additionally a significant effect of post type on liking behavior, however, no post type × nudge interaction (see Table C29). Deconstructing the significant main effect of post type, liking of social posts was significantly higher than liking of either true or false headlines (see Table C30).

Figure C9

Average Sharing of False, True and Social Posts Split Across Nudge Conditions in the 12.5% Misinformation Condition

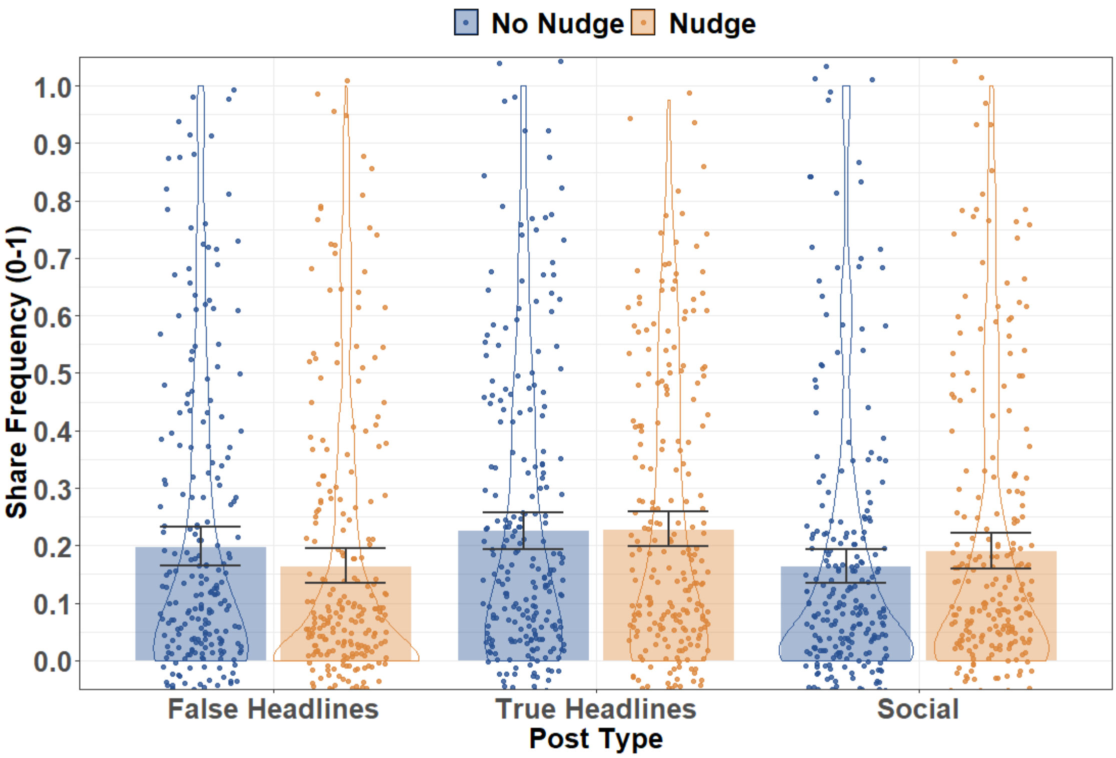

Note. Error bars represent 95% confidence intervals.

Table C27

ANODE (Type 3) Results for Sharing Behavior in 12.5% Condition, Comparing True, False, and Social Posts

Model: Share ~ Nudge Condition × Post Type (true, false, social) + (1 + Nudge | Post) + (1 + Post Type | Participant)

Fixed Effects	χ^2	*df*	*p*
Nudge	2.91	1	.089
Post Type	**19.93**	**2**	**<.001**
Nudge × Post Type	**9.12**	**2**	**.010**

Table C28

Pairwise Comparisons Comparing Sharing of Social Posts to Sharing of True and False Headlines in the 12.5% Misinformation Condition, Split by Nudge Conditions. Holm-Bonferroni adjustments made at 3 levels.

Nudge Condition	Contrast	β	*SE*	*z*	*p*
No Nudge	True – Social	**0.79**	**.18**	**4.43**	**<.001**
	False – Social	0.43	.25	1.70	.180
Nudge	True – Social	**0.54**	**.18**	**2.98**	**.006**
	False – Social	−0.20	.26	−0.77	.442

Figure C10

Average Liking of False, True and Social Posts Split Across Nudge Conditions in the 12.5% Misinformation Condition

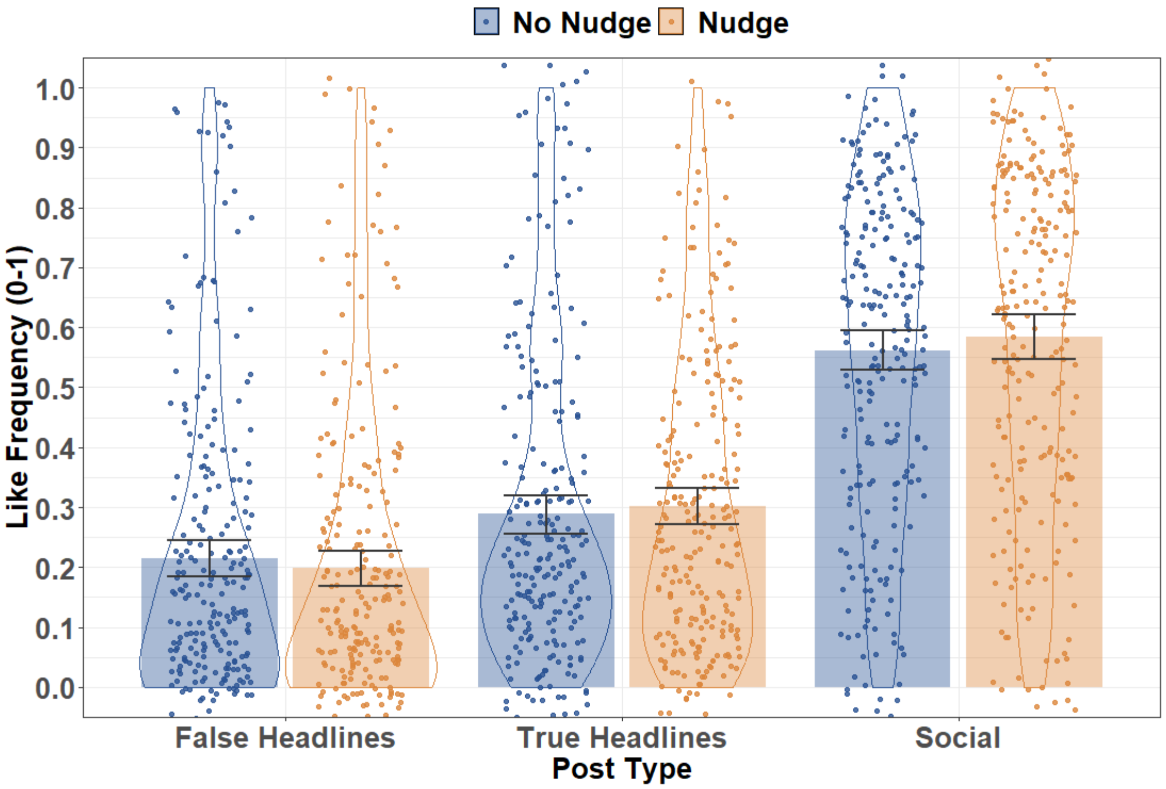

Note. Error bars represent 95% confidence intervals.

Table C29

ANODE Results for Liking Behavior in 12.5% Condition, Comparing True, False, and Social Posts

Model: Like ~ Nudge Condition × Post Type (true, false, social) + (1 + Nudge | Post) + (1 + Post Type | Participant)

Fixed Effects	χ^2	*df*	*p*
Nudge	0.66	1	.417
Post Type	**94.47**	**2**	**<.001**
Nudge × Post Type	3.90	2	.143

Table C30

Pairwise Comparisons Comparing Liking of Social Posts to Liking of True and False Headlines in the 12.5% Misinformation Condition, Collapsed across Nudge Conditions. Holm-Bonferroni adjustments made at 3 levels.

Contrast	β	*SE*	*z*	*p*
True – Social	**−2.64**	**.33**	**−8.05**	**<.001**
False – Social	**−1.81**	**.22**	**−8.28**	**<.001**

References

[1] Pennycook, G., McPhetres, J., Zhang, Y., Lu, J. G. & Rand, D. G. Fighting COVID-19 misinformation on social media: Experimental evidence for a scalable accuracy-nudge intervention. *Psychol Sci* **31**, 770–780 (2020)

[2] Roozenbeek, J., Freeman, A. L. J. & van der Linden, S. How accurate are accuracy-nudge interventions? A preregistered direct replication of Pennycook et al. (2020). *Psychol Sci* **32**, 1169–1178 (2021)
