# Supplementary material for: Nudge-based misinformation interventions are effective in information environments with low misinformation prevalence
Source: Sci Rep. 2024 May 20;14:11495. doi: 10.1038/s41598-024-62286-7 (PMC11106285; doi:10.1038/s41598-024-62286-7)
Supplement: Supplementary file 4 — Supplementary Information 4. [file 41598_2024_62286_MOESM4_ESM.pdf]

Supplementary materials for *"Nudge-Based Misinformation Interventions are Effective in Information Environments with Low Misinformation Prevalence"*

Supplement D – Complete results output for the impact of nudge and misinformation proportion conditions on both sharing and liking behavior (main analyses)

Table of Contents

Impact of misinformation proportion and nudge condition on sharing behavior 2

Assessing Sharing Behavior Prior to Exclusion Criteria being applied 4

Impact of misinformation proportion and nudge condition on liking behavior.........................5

Assessing Liking Behavior Prior to Exclusion Criteria being applied 7

Impact of misinformation proportion and nudge condition on engagement behavior..............7

Impact of misinformation proportion and nudge condition on sharing behavior

Average sharing behavior across conditions is presented in Figure D1, and results of analyses isolated to sharing behavior are presented in Tables D1 – D6.

Figure D1

Sharing Frequency for False and True Headlines Across Nudge and Misinformation Proportion Conditions

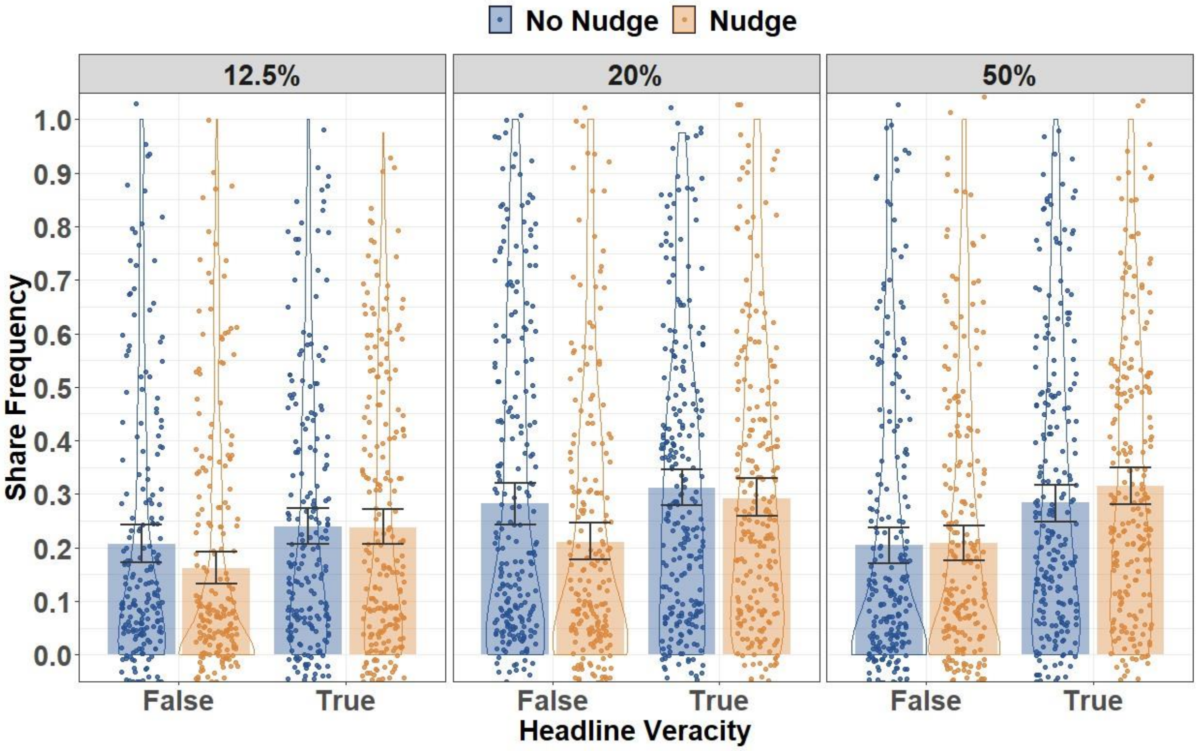

Note. Error bars represent 95% confidence intervals.

Table D1

ANODE Results for Sharing Behavior
Model: Share ~ Misinformation Proportion × Nudge Condition × Headline Veracity + (1 + Headline Veracity | Participant) + (1 +Misinformation Proprtion × Nudge Condition| Post), family = binomial , glmerControl(optimizer = "bobyqa")

Fixed Effects	χ^2	*df*	*p*
Misinformation proportion	**19.19**	**2**	**<.001**
Nudge	1.90	1	.168
Headline Veracity	**24.93**	**1**	**<.001**
Misinformation proportion × Nudge	3.50	2	.174
Misinformation proportion × Headline Veracity	**8.96**	**2**	**.011**
Nudge × Headline Veracity	**7.15**	**1**	**.007**
Misinformation proportion × Nudge × Headline Veracity	1.51	2	.470

Table D2

Pairwise Comparisons Assessing Impact of Misinformation Proportion Condition on Sharing Behaviour Across True and False Headlines, collapsed across Nudge Conditions

Condition	Levels of misinformation contrast	*Odds ratio*	*SE*	*z*	*p*
True	12.5% – 20%	**0.54**	**.08**	**−4.11**	**<.001**
	12.5% – 50%	**0.56**	**.08**	**−3.83**	**<.001**
	20% – 50%	1.04	.15	0.27	.785
False	12.5% – 20%	**0.48**	**.09**	**−3.92**	**<.001**
	12.5% – 50%	0.71	.13	−1.83	.068
	20% – 50%	1.48	.27	2.12	.068

Note. *p* Values are Holm-Bonferroni Adjusted for Three Tests

Table D3

Pairwise Comparisons Assessing Impact of Nudge (No Nudge vs. Nudge) on Sharing Behaviour Across Misinformation Proportion and True and False News Headlines

Headline Veracity	Misinformation proportion	*Odds ratio*	*SE*	*z*	*p*
True	Overall	0.95	.12	−0.43	.669
	12.5%	1.10	.25	0.43	.671
	20%	1.07	.21	0.33	.742
	50%	0.73	.15	−1.53	.127
False	Overall	**1.44**	**.22**	**2.42**	**.016**
	12.5%	**1.76**	**.49**	**2.05**	**.040**
	20%	**1.70**	**.41**	**2.22**	**.026**
	50%	0.96	.24	−0.15	.883

Note. *p* values are unadjusted.

Table D4

ANODE Results for Sharing Behavior in 12.5% misinformation condition. Model: Share ~ Nudge Condition × Headline Veracity + (1 + Headline Veracity | Participant) + (1 + Nudge | Post), family = binomial, glmerControl(optimizer = "bobyqa")

Fixed Effects	χ^2	*df*	*p*
Nudge	1.62	1	.203
Headline Veracity	**9.67**	**1**	**.002**
Nudge × Headline Veracity	**7.04**	**1**	**.008**

Table D5

ANODE Results for Sharing Behavior in 20% misinformation condition. Model: Share ~ Nudge Condition × Headline Veracity + (1 + Headline Veracity | Participant) + (1 + Nudge | Post), family = binomial , glmerControl(optimizer = "bobyqa")

Fixed Effects	χ^2	*df*	*p*
Nudge	1.67	1	.196
Headline Veracity	**8.48**	**1**	**.004**
Nudge × Headline Veracity	**10.92**	**1**	**<.001**

Table D6

ANODE Results for Sharing Behavior in 50% misinformation condition. Model: Share ~ Nudge Condition × Headline Veracity + (1 + Headline Veracity | Participant) + (1 + Nudge | Post), family = binomial , glmerControl(optimizer = "bobyqa")

Fixed Effects	χ^2	*df*	*p*
Nudge	0.86	1	.352
Headline Veracity	**64.23**	**1**	**<.001**
Nudge × Headline Veracity	3.49	1	.062

Assessing Sharing Behavior Prior to Exclusion Criteria being applied

The results presented in Table D7 assess the impact of misinformation proportion and nudge condition on sharing behavior prior to the application of exclusions criteria ($N = 1495$). The pattern of results is consistent with analyses with exclusion criteria applied.

Table D7

ANODE Results for Sharing Behavior
Model: Share ~ Misinformation Proportion × Nudge Condition × Headline Veracity + (1 + Headline Veracity | Participant) + (1 +Misinformation Proprtion × Nudge Condition| Post), family = binomial , glmerControl(optimizer = "bobyqa")

Fixed Effects	χ^2	*df*	*p*
Misinformation proportion	**18.28**	**2**	**<.001**
Nudge	0.96	1	.326
Headline Veracity	**22.14**	**1**	**<.001**
Misinformation proportion × Nudge	2.48	2	.290
Misinformation proportion × Headline Veracity	**8.97**	**2**	**.011**
Nudge × Headline Veracity	**5.78**	**1**	**.016**
Misinformation proportion × Nudge × Headline Veracity	0.48	2	.787

Impact of misinformation proportion and nudge condition on liking behavior

We then assessed the impact of the nudge intervention and misinformation proportion condition on liking behavior. Average liking of true and false posts across conditions is presented in Figure D2, and results of analyses isolated to liking behavior are presented in Tables D8 – D12.

Figure D2

Liking Frequency for False and True Headlines Across Nudge and Misinformation Proportion Conditions

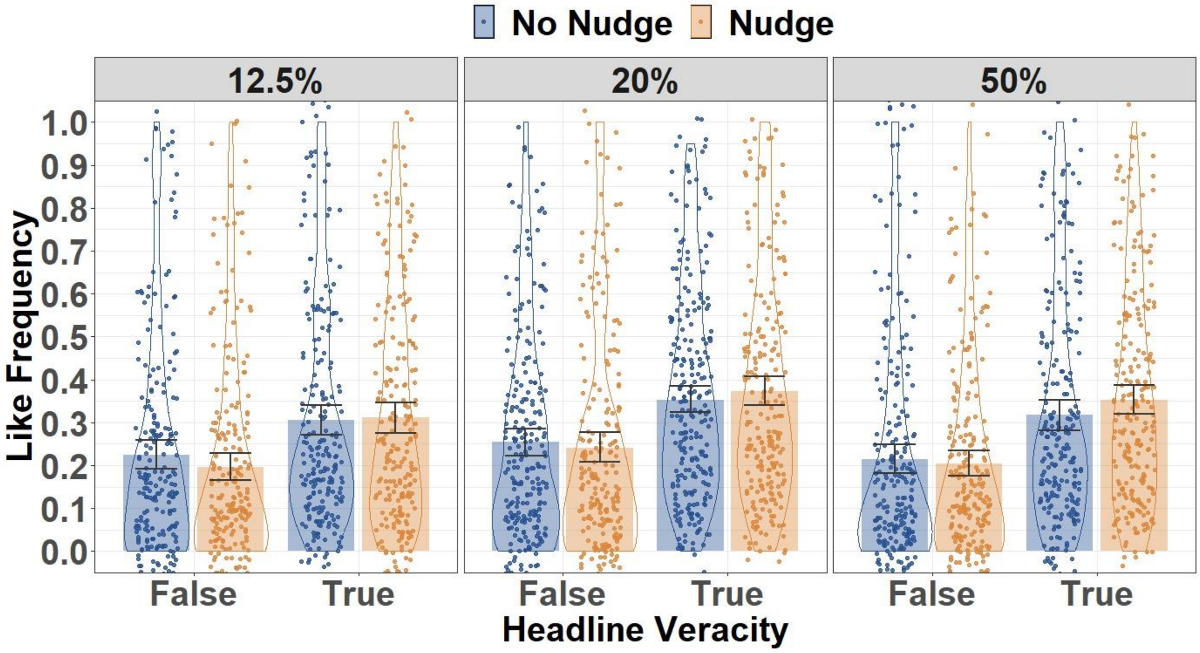

Note. Error bars represent 95% confidence intervals.

Table D8

ANODE Results for Liking Behavior
Model: Like ~ Misinformation Proportion × Nudge Condition × Headline Veracity + (1 + Headline Veracity | Participant) + (1 +Misinformation Proprtion + Nudge Condition| Post), family = binomial , glmerControl(optimizer = "bobyqa")

Fixed Effects	χ^2	*df*	*p*
Misinformation proportion	**12.60**	**2**	**.002**
Nudge	0.46	1	.500
Headline Veracity	**22.15**	**1**	**<.001**
Misinformation proportion × Nudge	1.05	2	.592
Misinformation proportion × Headline Veracity	3.32	2	.190
Nudge × Headline Veracity	3.50	1	.061
Misinformation proportion × Nudge × Headline Veracity	0.07	2	.968

Table D9

Pairwise Comparisons Assessing Impact of Misinformation Proportion Condition on Liking Behaviour, Collapsed Across Nudge Conditions and True and False Headlines

Levels of misinformation contrast	*Odds ratio*	*SE*	*z*	*p*
12.5% – 20%	**0.65**	**.08**	**−3.43**	**.002**
12.5% – 50%	0.88	.11	−1.05	.292
20% – 50%	**1.34**	**.17**	**2.37**	**.036**

Table D10

ANODE Results for Liking Behavior in 12.5% misinformation condition. Model: Like ~ Nudge Condition × Headline Veracity + (1 + Headline Veracity | Participant) + (1 + Nudge | Post), family = binomial , glmerControl(optimizer = "bobyqa")

Fixed Effects	χ^2	*df*	*p*
Nudge	0.41	1	.522
Headline Veracity	**8.23**	**1**	**.004**
Nudge × Headline Veracity	**4.29**	**1**	**.038**

Table D11

ANODE Results for Liking Behavior in 20% misinformation condition. Model: Like ~ Nudge Condition × Headline Veracity + (1 + Headline Veracity | Participant) + (1 + Nudge | Post), family = binomial , glmerControl(optimizer = "bobyqa")

Fixed Effects	χ^2	*df*	*p*
Nudge	0.03	1	.863
Headline Veracity	**7.90**	**1**	**.005**
Nudge × Headline Veracity	**6.30**	**1**	**.012**

Table D12

ANODE Results for Liking Behavior in 50% misinformation condition. Model: Like ~ Nudge Condition × Headline Veracity + (1 + Headline Veracity | Participant) + (1 + Nudge | Post), family = binomial , glmerControl(optimizer = "bobyqa")

Fixed Effects	χ^2	*df*	*p*
Nudge	0.53	1	.466
Headline Veracity	**40.53**	**1**	**<.001**
Nudge × Headline Veracity	3.56	1	.059

Assessing Liking Behavior Prior to Exclusion Criteria being applied

The results presented in Table D13 assess the impact of misinformation proportion and nudge condition on liking behavior prior to the application of exclusions criteria (N = 1495). The pattern of results is consistent with analyses with exclusion criteria applied.

Table D13

ANODE Results for Liking Behavior
Model: Like ~ Misinformation Proportion × Nudge Condition × Headline Veracity + (1 + Headline Veracity | Participant) + (1 +Misinformation Proprtion + Nudge Condition| Post), family = binomial , glmerControl(optimizer = "bobyqa")

Fixed Effects	χ^2	*df*	*p*
Misinformation proportion	**12.12**	**2**	**.002**
Nudge	<.01	1	.995
Headline Veracity	**21.06**	**1**	**<.001**
Misinformation proportion × Nudge	<.01	2	.999
Misinformation proportion × Headline Veracity	3.66	2	.160
Nudge × Headline Veracity	2.76	1	.097
Misinformation proportion × Nudge × Headline Veracity	0.10	2	.951

Impact of misinformation proportion and nudge condition on engagement behavior

The following results analyse the impact of misinformation proportion and nudge condition on engagement likelihood, and thus engagement here is a binary outcome whereby 0 = did not engage and 1 = did engage (i.e., like, share, or like and share). This analysis is entirely exploratory and results should be treated as such, though we note that the pattern of results closely mirrors those of (1) engagement treated as an ordinal factor, and (2) sharing behaviour. Average engagement with true and false posts across conditions is presented in

Figure D3, and results of analyses assessing engagement behavior are presented in Tables D14 – D18. Deconstructing the significant nudge × headline veracity interaction, there was a significant effect of the nudge on engagement with false headlines, *OR* = 1.37, *SE* = 0.19, z = 2.31, p = .021, with engagement significantly lower in the nudge than the no nudge condition. By contrast, there was no significant effect of the nudge intervention on engagement with true headlines, *OR* = 0.94, *SE* = 0.10, $z = -0.57$, p = .566. The primary point where the binary engagement results deviate from sharing behavior is when results are isolated to only the 50% misinformation condition: For engagement behavior there is a significant nudge × headline veracity interaction (i.e., the nudge lead to a significant improvement in engagement discernment, p = .024), whereas this interaction was non-significant for sharing behavior (p = .062).

Figure D3

Engagement Frequency for False and True Headlines Across Nudge and Misinformation Proportion Conditions

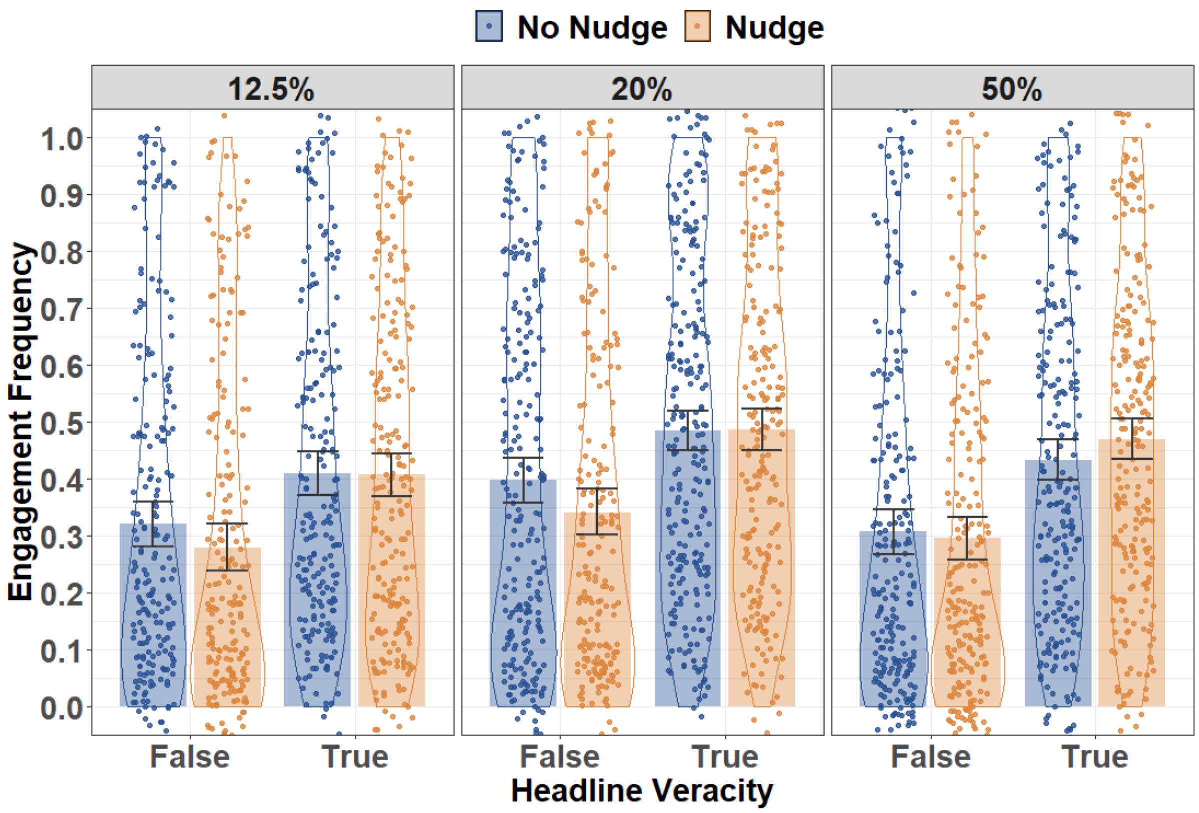

Table D14

ANODE (Type 3) Results for Engagement Behavior
Model: Engage ~ Misinformation Proportion × Nudge Condition × Headline Veracity + (1 + Headline Veracity | Participant) + (1 +Misinformation Proprtion × Nudge Condition| Post), family = binomial , glmerControl(optimizer = "bobyqa")

Fixed Effects	χ^2	*df*	*p*
Misinformation proportion	**17.66**	**2**	**<.001**
Nudge	1.33	1	.249
Headline Veracity	**29.35**	**1**	**<.001**
Misinformation proportion × Nudge	1.08	2	.583
Misinformation proportion × Headline Veracity	2.89	2	.236
Nudge × Headline Veracity	**4.50**	**1**	**.034**
Misinformation proportion × Nudge × Headline Veracity	0.24	2	.885

Table D15

Pairwise Comparisons Assessing Impact of Misinformation Proportion Condition on Engagement Behaviour Across True and False Headlines, collapsed across Nudge Conditions

Levels of misinformation contrast	*Odds ratio*	*SE*	*z*	*p*
12.5% – 20%	**0.56**	**.08**	**−4.16**	**<.001**
12.5% – 50%	0.77	.11	−1.85	.064
20% – 50%	**1.38**	**.19**	**2.31**	**.042**

Note. *p* Values are Holm-Bonferroni Adjusted for Three Tests

Table D16

ANODE Results for Engagement Behavior in 12.5% misinformation condition. Model: Like ~ Nudge Condition × Headline Veracity + (1 + Headline Veracity | Participant) + (1 + Nudge | Post), family = binomial, glmerControl(optimizer = "bobyqa")

Fixed Effects	χ^2	*df*	*p*
Nudge	1.21	1	.271
Headline Veracity	**9.90**	**1**	**.002**
Nudge × Headline Veracity	**5.10**	**1**	**.024**

Table D17

ANODE Results for Engagement Behavior in 20% misinformation condition. Model: Like ~ Nudge Condition × Headline Veracity + (1 + Headline Veracity | Participant) + (1 + Nudge | Post), family = binomial, glmerControl(optimizer = "bobyqa")

Fixed Effects	χ^2	*df*	*p*
Nudge	0.41	1	.522
Headline Veracity	**9.77**	**1**	**.002**
Nudge × Headline Veracity	**8.98**	**1**	**.003**

Table D18

ANODE Results for Engagement Behavior in 50% misinformation condition. Model: Like ~ Nudge Condition × Headline Veracity + (1 + Headline Veracity | Participant) + (1 + Nudge | Post), family = binomial, glmerControl(optimizer = "bobyqa")

Fixed Effects	χ^2	*df*	*p*
Nudge	0.06	1	.801
Headline Veracity	**51.99**	**1**	**<.001**
Nudge × Headline Veracity	**5.08**	**1**	**.024**
