# Supplementary material for: Nudge-based misinformation interventions are effective in information environments with low misinformation prevalence
Source: Sci Rep. 2024 May 20;14:11495. doi: 10.1038/s41598-024-62286-7 (PMC11106285; doi:10.1038/s41598-024-62286-7)
Supplement: Supplementary file 5 — Supplementary Information 5. [file 41598_2024_62286_MOESM5_ESM.pdf]

Supplementary materials for *"Nudge-Based Misinformation Interventions are Effective in Information Environments with Low Misinformation Prevalence"*

Supplement E – Supplementary Results focusing on Engagement Behavior (Outdated as of April 2024)

Table of Contents

Preamble 2

Assumption check 2

Belief 3

Political Orientation 4

Isolating results to first 50 posts 7

The effect of post order on engagement behavior 8

Interaction with social posts in the 12.5% misinformation condition 12

Cross-validating results using cumulative link models with cluster robust standard errors 13

References 17

Preamble

The below results focus on *engagement* behavior, whereby engagement behavior is an ordinal variable measured on an ordinal scale (0 = no engagement, 1 = like, 2 = share, 3 = like and share). These analyses are in line with the initial preregistered plan, however, due to convergence errors resulting in a reliance on suboptimal statistical models (as outlined in the main text) the decision was made to deviate from using engagement behavior as the primary outcome variable, and thus the below results are now outdated (as of April 2024). The original supplementary results are presented below for transparency, main analyses focusing on engagement behavior are presented in Supplement B.

Assumption check

For participants in the 50% misinformation conditions, we initially checked to ensure engagement with the 10 target false headlines was not significantly different from engagement with the 30 filler headlines (see Figure C11 for mean level of engagement across filler [left] and target [right] false posts). To do so, we ran an ANODE on a CLMM model including post type (target, filler), nudge condition, and their interaction. Prior to statistical analysis, post type was dummy coded and centred. There was no significant difference in level of engagement with the target and filler false posts (see Table E1). Although we initially pre-registered including all 40 claims presented in the 50% misinformation condition in the main analysis, due to missingness, adding all false headlines presented in the 50% condition to the model makes the findings uninterpretable when compared to the other conditions. Thus, to allow for all misinformation proportion conditions to be directly comparable, we have slightly deviated from the pre-registered analysis plan and removed the 30 filler false posts from all main analyses.

Table E1

ANODE Results for Engagement Behavior in 50% Misinformation Condition, Comparing Target and Filler False Posts
Model: Engagement ~ Nudge Condition × Headline Type (target vs filler) + (1 | Post) + (1| Participant)

Fixed Effects	χ^2	*df*	*p*
Nudge	1.00	1	.316
Headline Type	0.10	1	.757
Nudge × Headline Veracity	0.17	1	.682

Figure E1

Level of Engagement with Filler (Left) and Target (Right) False Posts Across the No Nudge and Nudge Conditions in the 50% Misinformation Condition

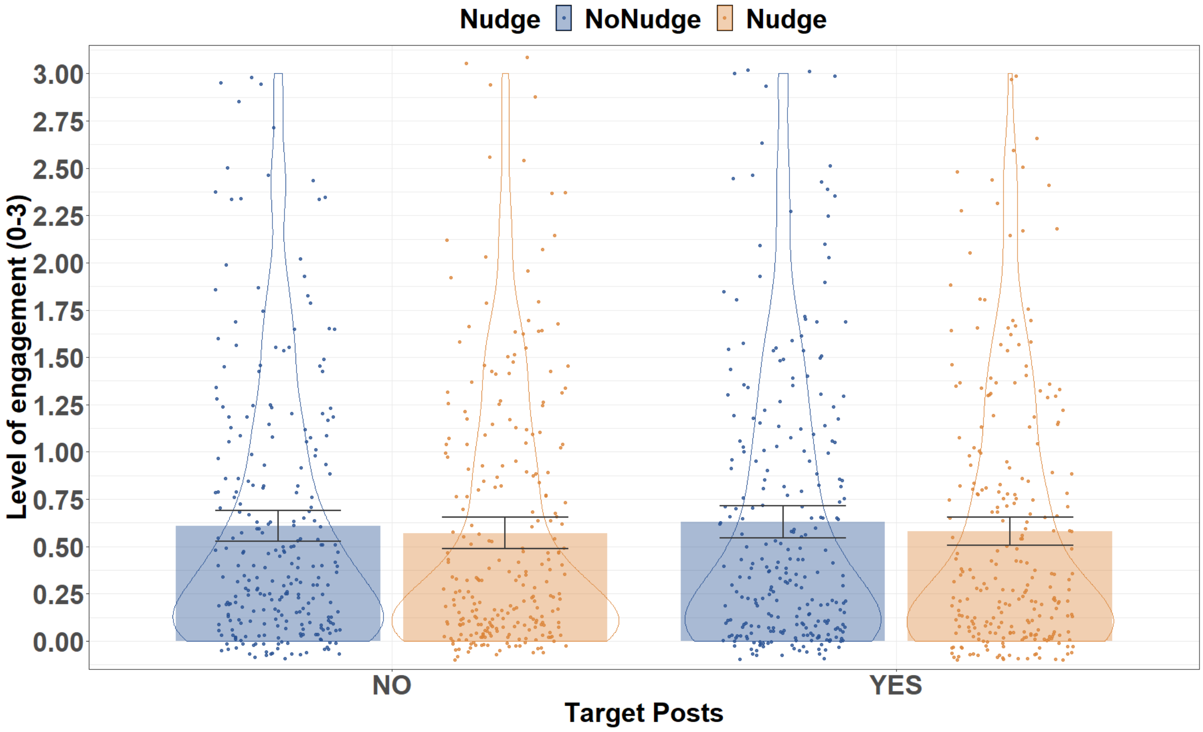

Note. Error bars represent 95% confidence intervals.

Belief

As with sharing and liking behavior, we correlated belief with the impact of the nudge on engagement behavior (i.e., a difference score created by calculating $M_{\text{Nudge}} - M_{\text{No Nudge}}$ for each post) across the three misinformation proportion conditions (see Figure E2). As with prior research (e.g., Pennycook et al., 2020; Roozenbeek et al., 2021), belief was significantly correlated with nudge impact across all three misinformation proportion conditions (12.5%

misinformation; $r = .61$, $p < .001$, 20% misinformation; $r = .56$, $p < .001$, 50% misinformation; $r = .62$, $p < .001$).

Figure E2

Scatterplot (With Best-Fitting Regression Line) Depicting Association Between Average Belief (Averaged Across Nudge Conditions) and Average Difference in Engagement ($M_{nudge} - M_{no\ Nudge}$) With Each False Headline (Blue) and True Headline (Orange) in the Nudge Compared to the No Nudge Condition

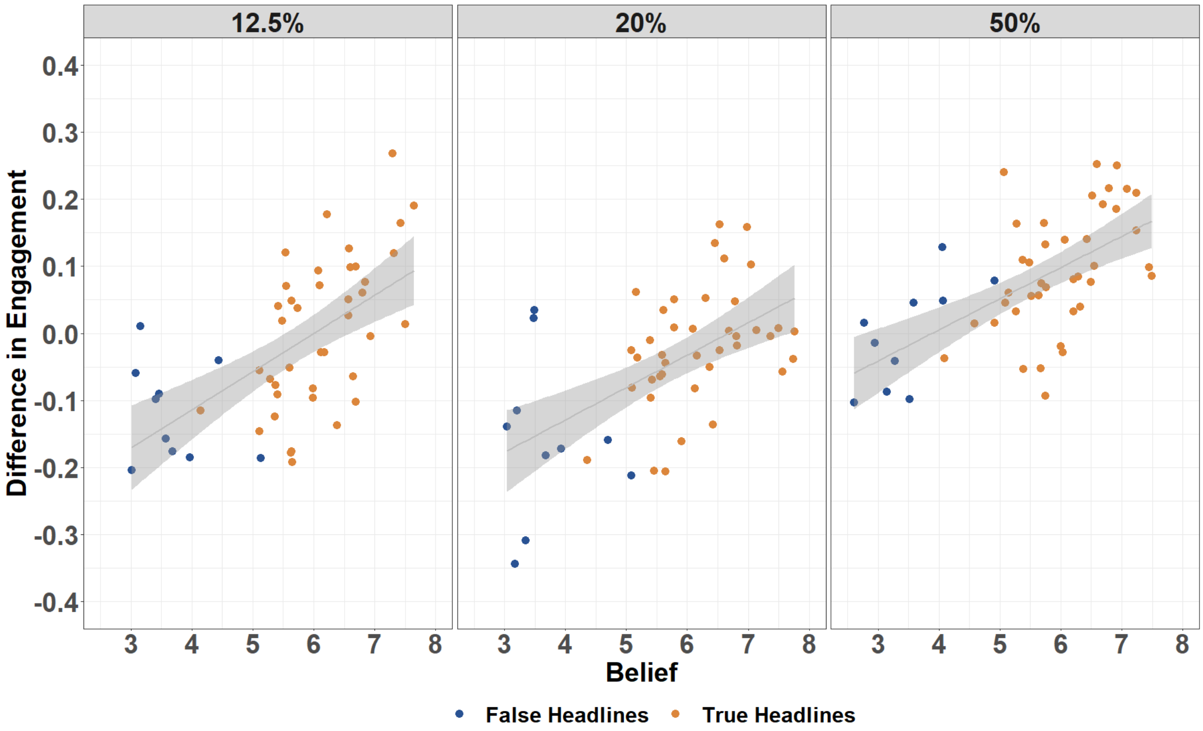

Note. For difference in engagement, any value below 0 indicates that average engagement with the headline was lower in the nudge than in the no nudge condition, and any value above 0 indicates average engagement with the headline was higher in the nudge than the no nudge condition. Error bars represent standard error.

Political Orientation

We pre-registered to assess whether the effect of the nudge intervention significantly differed over the political spectrum. To do so, political orientation (1-7) was treated as a continuous predictor in the model, and analyses were run separately for each misinformation proportion condition (see Tables E2 – E4). Across all three misinformation proportion conditions there were no significant political orientation × nudge interactions, or three-way political orientation × nudge × headline veracity interactions. Results split by political orientation (liberal, conservative) are displayed in Figure E3. We do note that there were

main effects of political orientation and political orientation × headline veracity interactions, with more conservative participants seemingly engaging more than more liberal participants with both false and true headlines, however, this difference was greater for false headlines.

Table E2

ANODE Results for Engagement Behavior including political orientation (1-7) as a continuous factor, isolated to the 12.5% misinformation condition. Model: Engagement ~ Post Order × Nudge Condition × Headline Veracity + (1 | Participant)

Fixed Effects	χ^2	*df*	*p*
Political orientation	**1263.91**	**1**	**<.001**
Nudge	0.10	1	.753
Headline veracity	**248.31**	**1**	**<.001**
Political orientation × Nudge	0.98	1	.322
Political orientation × Headline veracity	**78.63**	**1**	**<.001**
Nudge × Headline veracity	**15.51**	**1**	**<.001**
Political orientation × Nudge × Headline veracity	1.18	1	.277

Table E3

ANODE Results for Engagement Behavior including political orientation (1-7) as a continuous factor, isolated to the 20% misinformation condition. Model: Engagement ~ Post Order × Nudge Condition × Headline Veracity + (1 | Participant)

Fixed Effects	χ^2	*df*	*p*
Political orientation	**1962.32**	**1**	**<.001**
Nudge	0.03	1	.863
Headline veracity	**252.37**	**1**	**<.001**
Political orientation × Nudge	0.35	1	.554
Political orientation × Headline veracity	**114.40**	**1**	**<.001**
Nudge × Headline veracity	**22.20**	**1**	**<.001**
Political orientation × Nudge × Headline veracity	0.75	1	.386

Table E4

ANODE Results for Engagement Behavior including political orientation (1-7) as a continuous factor, to the 50% misinformation condition. Model: Engagement ~ Post Order × Nudge Condition × Headline Veracity + (1 | Participant)

Fixed Effects	χ^2	*df*	*p*
Political orientation	**1990.09**	**1**	**<.001**
Nudge	1.69	1	.194
Headline veracity	**469.72**	**1**	**<.001**
Political orientation × Nudge	0.66	1	.416
Political orientation × Headline veracity	**115.34**	**1**	**<.001**
Nudge × Headline veracity	**17.69**	**1**	**<.001**
Political orientation × Nudge × Headline veracity	0.78	1	.377

Figure E3

Engagement with true and false headlines split by misinformation proportion and nudge conditions for liberal (left) and conservative (right) participants.

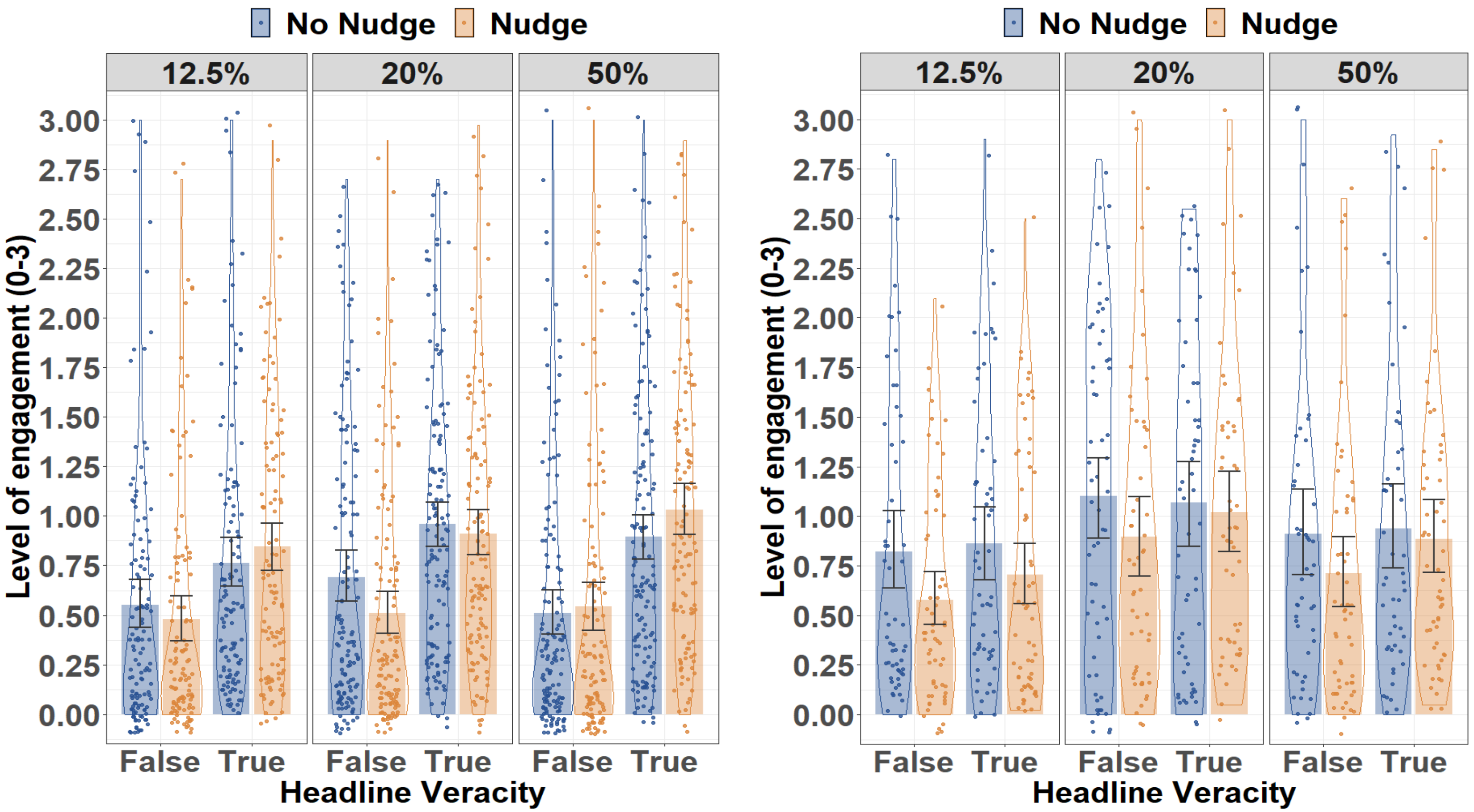

Note. Participants who responded 1 (strongly liberal) – 3 (leaning liberal) on the political orientation question were classed as liberal, and those who responded 5 (leaning conservative) – 7 (strongly conservative) were classed as conservative for graphical purposes. Participants who reported to be centralists are not presented in the graphs. Error bars represent 95% confidence intervals.

Isolating results to first 50 posts

To assess whether the higher average level of engagement with true and false posts in the 20% misinformation condition was driven by the reduced number of headlines presented in the condition (50 compared to 80) we reran the main analysis isolated to the first 50 posts participants were presented (see Table E5, average engagement across conditions and headline type for the first 50 posts displayed in each condition is displayed in Figure E4). This analysis was not pre-registered and thus is entirely exploratory. The pattern of results remains consistent with the full dataset. As such, it appears that the different level of engagement with true and false headlines across misinformation proportion conditions was not primarily driven by the reduced number of posts in the 20% misinformation condition.

Table E5

ANODE Results for Engagement Behavior, Isolated to the First 50 Posts Participants Saw
Model: Engagement ~ Misinformation Proportion × Nudge Condition × Headline Veracity + (1 | Participant)

Fixed Effects	χ^2	*df*	*p*
Misinformation proportion	**12.09**	**2**	**.002**
Nudge	0.09	1	.760
Headline Veracity	**699.41**	**1**	**<.001**
Misinformation proportion × Nudge	2.24	2	.326
Misinformation proportion × Headline Veracity	**24.58**	**2**	**<.001**
Nudge × Headline Veracity	**46.05**	**1**	**<.001**
Misinformation proportion × Nudge × Headline Veracity	1.01	2	.603

Figure E4

Engagement With True and False Headlines Across Nudge and Misinformation Proportion Conditions, Isolated to the First 50 Posts Participants were Exposed To

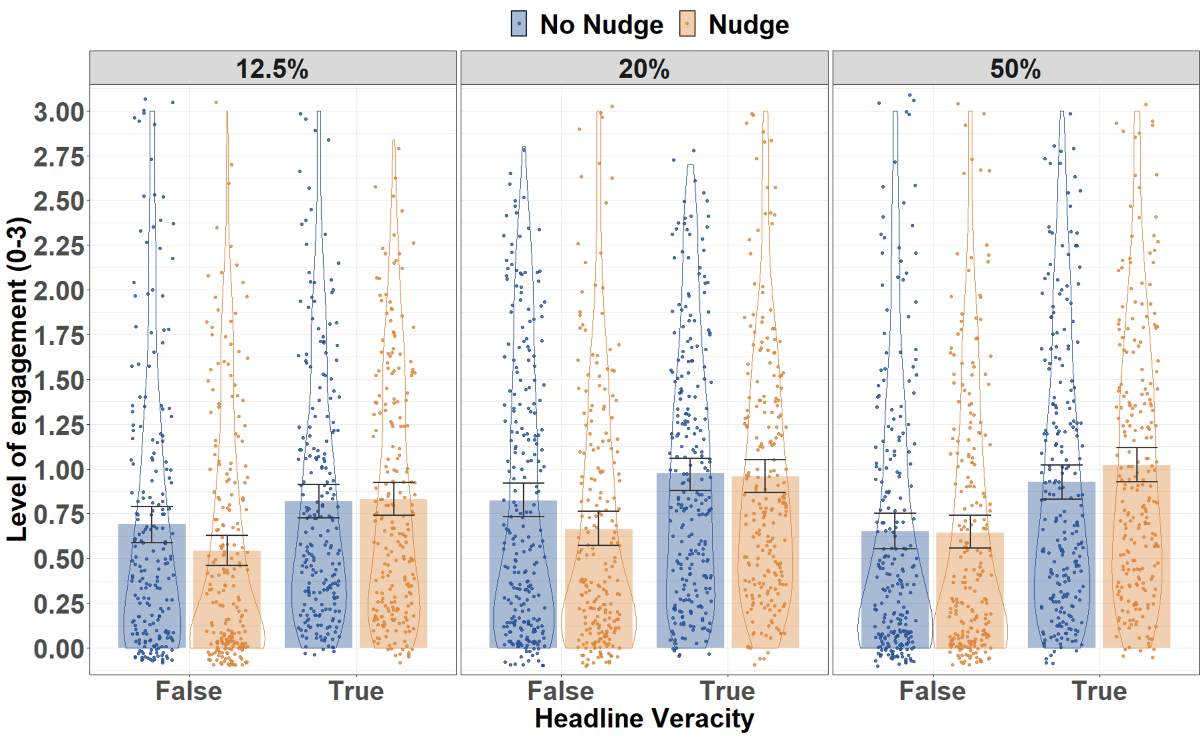

Note. Error bars represent 95% confidence intervals.

The effect of post order on engagement behavior

Due to prior research suggesting nudge-based interventions may only be effective on the first few posts a participant is exposed to (see Roozenbeek et al., 2021), we ran analyses assessing if the effect of the nudge decreased over the course of the study. Specifically, post display order (1-50, or 1-80 dependent on condition) was included in the statistical models as a continuous predictor, and analyses were run separately for each misinformation proportion condition. These analyses were not pre-registered and thus are entirely exploratory. Engagement with false and true headlines over the course of the experiment is presented in Figure E5, and ANODE results split by misinformation proportion (12.5%, 20%, and 50%) condition are displayed in Tables E6-E8. Although there was a significant effect of post order across all conditions, with people engaging less with posts at the end of the experiment than

the beginning, there were no significant post order × nudge interactions, or three-way post order × nudge × headline veracity interactions across any of the misinformation proportion conditions (all *p*s > .073). This suggests that the effect of the nudge intervention did not significantly reduce over the course of the study, however, it is likely that the current study was underpowered to detect this effect. In fact, we note that visual inspection of the graphs for the 12.5% suggests that the effect of the nudge on engagement was numerically more pronounced at the start of the task than at the end (moreover, at the end of the study there was minimal numeric difference in engagement with false headlines between the nudge and no nudge conditions). As such, numerically, the results suggest that the nudge intervention did decay relatively quickly post exposure within this condition. However, we cannot make any specific claims about whether this decay would generalize across conditions or stimuli, or whether this reflects a genuine reduction in the effectiveness of the nudge intervention or is a pattern that occurred by chance.

Table E6

ANODE Results for Engagement Behavior over the Course of the Experiment, Isolated to the 12.5% Misinformation Condition
Model: Engagement ~ Post Order × Nudge Condition × Headline Veracity + (1 | Participant)

Fixed Effects	χ^2	*df*	*p*
Post order	**146.21**	**1**	**<.001**
Nudge	0.24	1	.628
Headline veracity	**239.05**	**1**	**<.001**
Post order × Nudge	3.21	1	.073
Post order × Headline veracity	0.03	1	.874
Nudge × Headline veracity	**14.04**	**1**	**<.001**
Post order × Nudge × Headline veracity	0.08	1	.779

Table E7

ANODE Results for Engagement Behavior over the Course of the Experiment, Isolated to the 20% Misinformation Condition
Model: Engagement ~ Post Order × Nudge Condition × Headline Veracity + (1 | Participant)

Fixed Effects	χ^2	*df*	*p*
Post order	**58.56**	**1**	**<.001**
Nudge	0.07	1	.789
Headline veracity	**249.99**	**1**	**<.001**
Post order × Nudge	< 0.01	1	.929
Post order × Headline veracity	0.12	1	.726
Nudge × Headline veracity	**25.41**	**1**	**<.001**
Post order × Nudge × Headline veracity	0.50	1	.478

Table E8

ANODE Results for Engagement Behavior over the Course of the Experiment, Isolated to the 50% Misinformation Condition
Model: Engagement ~ Post Order × Nudge Condition × Headline Veracity + (1 | Participant)

Fixed Effects	χ^2	*df*	*p*
Post order	**126.68**	**1**	**<.001**
Nudge	1.88	1	.171
Headline veracity	**472.14**	**1**	**<.001**
Post order × Nudge	0.59	1	.444
Post order × Headline veracity	1.45	1	.229
Nudge × Headline veracity	**9.86**	**1**	**.002**
Post order × Nudge × Headline veracity	0.44	1	.509

Figure E5

Engagement with false (left panel) and true (right panel) headlines over the course of the experiment. The blue line shows participant engagement in the no nudge condition, and the orange line shows participant engagement in the nudge condition.

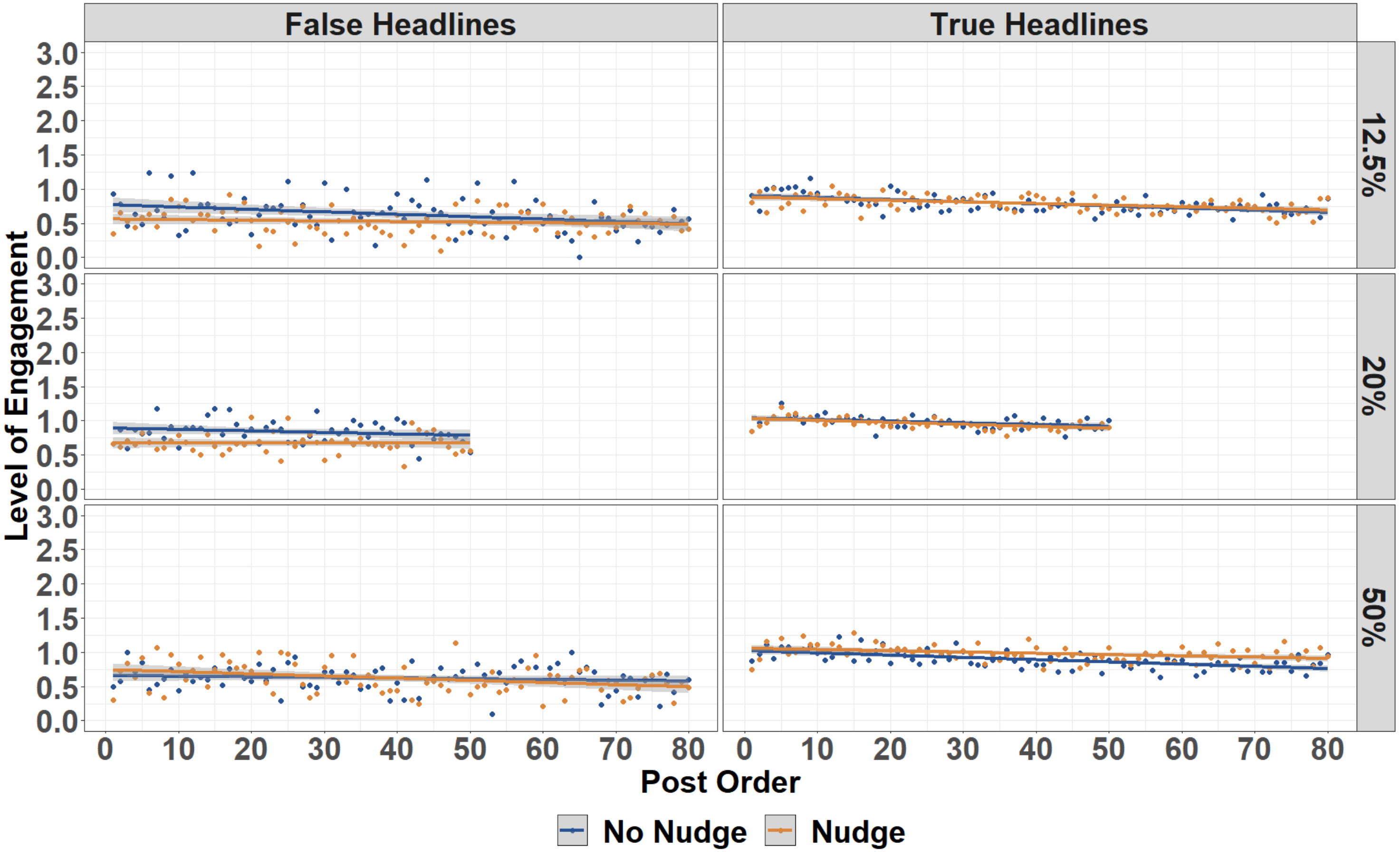

Note. Error bars represent standard error.

Interaction with social posts in the 12.5% misinformation condition

We additionally assessed how people interacted with social posts compared to true and false news in the 12.5% misinformation condition (i.e., the only condition including social posts). Engagement with each post type across nudge conditions is shown in Figure E6. There was a significant main effect of post type, qualified by a significant post type nudge condition interaction (see Table E9). Follow-up contrasts reveal that participants engaged with social posts significantly more than either true or false headlines across both nudge conditions; see Table E10.

Figure E6

Average Engagement with False, True and Social Posts Split Across Nudge Conditions in the 12.5% Misinformation Condition

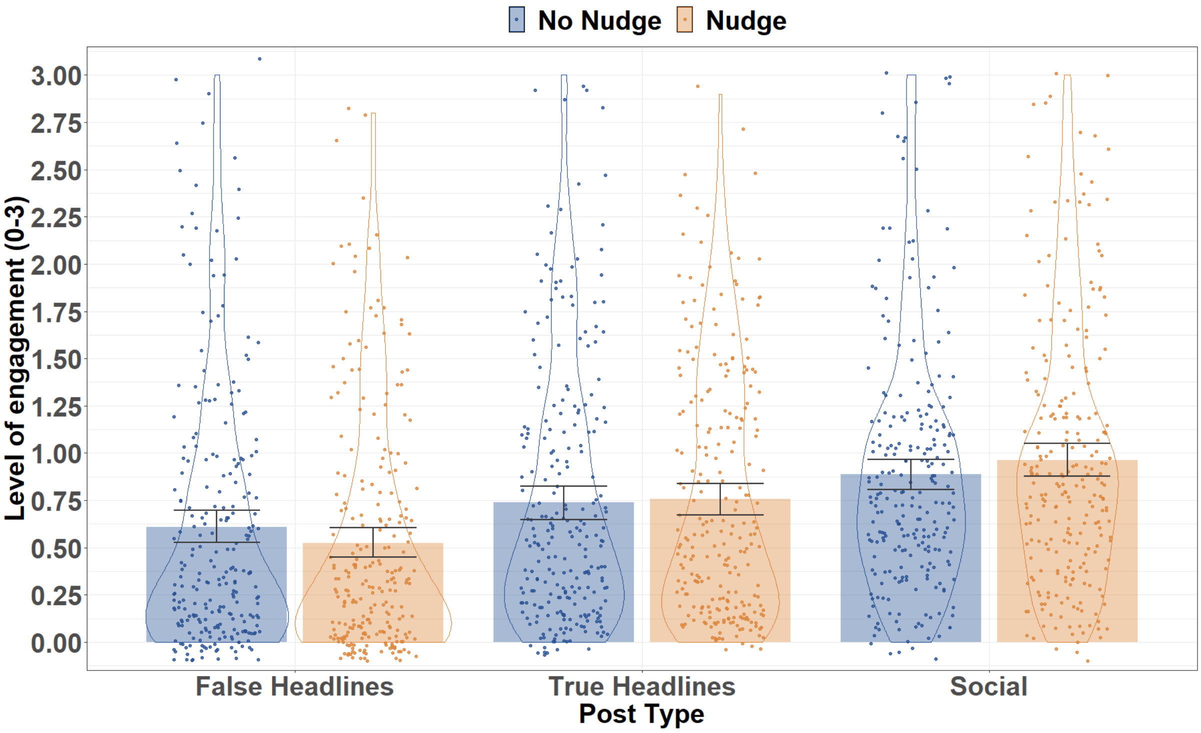

Note. Error bars represent 95% confidence intervals.

Table E9

ANODE Results for Engagement Behavior in 12.5% Condition, Comparing True, False, and Social Posts
Model: Engagement ~ Nudge Condition × Post Type (true, false, social) + (1 | Post) + (1| Participant)

Fixed Effects	χ^2	*df*	*p*
Nudge	0.08	1	.782
Post Type	**36.64**	**2**	**<.001**
Nudge × Post Type	**29.63**	**2**	**<.001**

Table E10

Pairwise Comparisons Comparing Engagement with Social Posts to Engagement with True and False Headlines in the 12.5% Misinformation Condition

Nudge Condition	Contrast	β	*SE*	*z*	*p*
No Nudge	Social – True	**0.65**	**.14**	**4.62**	**<.001**
	Social – False	**1.08**	**.21**	**5.07**	**<.001**
Nudge	Social – True	**0.75**	**.14**	**5.34**	**<.001**
	Social – False	**1.50**	**.21**	**7.02**	**<.001**

Note. Holm-Bonferroni corrections are made on three levels with contrasts for the nudge and no nudge conditions conducted separately.

Cross-validating results using cumulative link models with cluster robust standard errors

Due to the convergence failures in the cumulative link mixed effects models, we additionally ran exploratory analyses using cumulative-link models (clm) with cluster-robust standard errors, clustered on participant and headline, calculated using the *vcorCL* function of the *Sandwich* R package [2]. We note that to the best of our knowledge there are no packages to conduct ANODEs on cumulative link models with cluster robust standard errors in R, and thus the results cannot always be directly compared to the main analyses. As such, for clarity we have deconstructed the results and conducted analyses at each misinformation proportion. However, this limits our ability to compare the relative effect of the nudge across misinformation proportion conditions. Nonetheless, the pattern of results was comparable to those of the clmm models including participant random intercepts only. However, in the 12.5% misinformation condition the impact of the nudge intervention on engagement with

false headlines changed from marginally significant (p = .039) to non-significant (p = .063).

Results for these supplementary analyses are presented in Tables E11 – E17.

Table E11

Engagement Behavior Predicted by Misinformation Proportion, Nudge Condition, Headline Type, and Their Interactions. 12.5% Misinformation Condition Used as Reference Group in Model.

Condition	β	*SE*	*t*	*p*
Misinformation proportion – 20%	**0.32**	**.08**	**4.03**	**<.001**
Misinformation proportion – 50%	0.16	.08	1.91	.056
Nudge	−0.10	.11	−0.87	.384
Headline veracity	**−0.45**	**.18**	**−2.54**	**.011**
Misinformation proportion – 20% × Nudge	−0.03	.16	−0.18	.857
Misinformation proportion – 50% × Nudge	0.18	.16	1.10	.272
Misinformation proportion – 20% × Headline veracity	0.02	.05	0.35	.728
Misinformation proportion – 50% × Headline veracity	**−0.15**	**.07**	**−2.30**	**.021**
Nudge × Headline veracity	**−0.23**	**.06**	**−3.70**	**<.001**
Misinformation proportion – 20% × Nudge × Headline veracity	−0.03	.09	−0.28	.783
Misinformation proportion – 50% × Nudge × Headline veracity	0.04	.11	0.37	.715

Table E12

Engagement Behavior Predicted by Nudge Condition, Headline Type, and Their Interactions in the 12.5% Misinformation Condition

Condition	β	*SE*	*t*	*p*
Nudge	−0.10	.12	−0.87	.385
Headline veracity	**−0.45**	**.18**	**−2.54**	**.011**
Nudge × Headline veracity	**−0.23**	**.06**	**−3.70**	**<.001**

Table E13

Engagement Behavior Predicted by Nudge Condition, Headline Type, and Their Interactions in the 20% Misinformation Condition

Condition	β	*SE*	*t*	*p*
Nudge	−0.13	.11	−1.20	.230
Headline veracity	**−0.43**	**.16**	**−2.72**	**.006**
Nudge × Headline veracity	**−0.26**	**.08**	**−3.19**	**.001**

Table E14

Engagement Behavior Predicted by Nudge Condition, Headline Type, and Their Interactions in the 50% Misinformation Condition

Condition	β	*SE*	*t*	*p*
Nudge	0.07	.11	0.68	.495
Headline veracity	**−0.59**	**.14**	**−4.24**	**<.001**
Nudge × Headline veracity	**−0.19**	**.09**	**−2.17**	**.030**

Table E15

Engagement Predicted by Nudge Condition, Split by Headline Veracity and Misinformation Proportion Condition

Headline veracity	Misinformation proportion condition	β	*SE*	*t*	*p*
True	12.5%	<−0.01	.12	<−0.01	.995
	20%	−0.02	.10	−0.18	.860
	50%	0.15	.11	1.45	.146
False	12.5%	−0.23	.12	−1.86	.063
	20%	**−0.27**	**.13**	**−2.11**	**.035**
	50%	−0.03	.13	−0.25	.801

Table E16

Sharing Behavior Predicted by Misinformation Proportion, Nudge Condition, Headline Type, and Their Interactions. 12.5% Misinformation Condition Used as Reference Group in Model

Condition	β	*SE*	*z*	*p*
Intercept	−1.30	.09	−14.92	<.001
Misinformation proportion – 20%	0.34	.09	3.83	<.001
Misinformation proportion – 50%	0.24	.09	2.61	.009
Nudge	−0.13	.14	−0.99	.321
Headline veracity	−0.33	.13	−2.56	.010
Misinformation proportion – 20% × Nudge	−0.09	.18	−0.48	.632
Misinformation proportion – 50% × Nudge	0.23	.18	1.26	.206
Misinformation proportion – 20% × Headline veracity	0.05	.06	0.81	.418
Misinformation proportion – 50% × Headline veracity	−0.16	.07	−2.34	.019
Nudge × Headline veracity	−0.29	.09	−3.24	.001
Misinformation proportion – 20% × Nudge × Headline veracity	<−0.01	.12	−0.04	.968
Misinformation proportion – 50% × Nudge × Headline veracity	0.17	.13	1.35	.177

Table E17

Liking Behavior Predicted by Misinformation Proportion, Nudge Condition, Headline Type, and Their Interactions. 12.5% Misinformation Condition Used as Reference Group in Model

Condition	β	*SE*	*z*	*p*
Intercept	−1.03	.12	−8.52	<.001
Misinformation proportion – 20%	0.23	.08	2.93	.003
Misinformation proportion – 50%	0.07	.08	0.80	.422
Nudge	−0.05	.12	−0.46	.644
Headline veracity	−0.52	.24	−2.20	.028
Misinformation proportion – 20% × Nudge	0.07	.16	0.47	.642
Misinformation proportion – 50% × Nudge	0.11	.16	0.70	.485
Misinformation proportion – 20% × Headline veracity	−0.03	.05	−0.54	.588
Misinformation proportion – 50% × Headline veracity	−0.13	.07	−1.92	.055
Nudge × Headline veracity	−0.20	.05	−3.78	<.001
Misinformation proportion – 20% × Nudge × Headline veracity	0.05	.08	0.62	.537
Misinformation proportion – 50% × Nudge × Headline veracity	−0.02	.11	−0.15	.877

References

[1] Roozenbeek, J., Freeman, A. L. J. & van der Linden, S. How accurate are accuracy-nudge interventions? A preregistered direct replication of Pennycook et al. (2020). *Psychol Sci* **32**, 1169–1178 (2021)

[2] Zeileis, A., Köll, S. & Graham, N. Various versatile variances: An object-oriented implementation of clustered covariances in R. *J Stat Softw* **95**, (2020)
